# Pilates and AR/VR-based and other technology-assisted exercise for balance and postural control in older-adult and aging-related rehabilitation contexts: a systematic review and exploratory network meta-analysis

**DOI:** 10.3389/fpubh.2026.1899309

**Published:** 2026-08-07

**Authors:** Yongheng Zhao, Tae-Jin Son, Minhye Shin

**Affiliations:** 1 Department of Sports Coaching, Kyungil University, Gyeongsan-si, Republic of Korea; 2 Wushu College, Henan University, Kaifeng, China; 3 Department of Taekwondo, Kyungil University, Gyeongsan-si, Republic of Korea

**Keywords:** balance, digital health, fall risk, network meta-analysis, older adults, pilates, postural control, technology-assisted exercise

## Abstract

**Background:**

Population aging has increased demand for scalable strategies supporting balance-oriented rehabilitation, fall-risk management, and functional independence in older adults. Pilates and technology-assisted exercise, including AR/VR, exergaming, motion-sensing exercise, telerehabilitation, and technology-delivered Pilates, may be relevant, but comparative evidence and older-adult applicability remain uncertain.

**Objective:**

To compare Pilates, technology-assisted exercise, technology-delivered Pilates, conventional exercise, and control for balance- and postural-control-related outcomes using exploratory network meta-analysis and aging-public-health applicability mapping.

**Methods:**

We searched eight sources through 2 May 2026. The primary network included source-verified randomized trials with eligible balance/postural-control endpoints and extractable post-intervention arm-level data. Hedges’ g used exact small-sample correction, and random-effects frequentist network meta-analysis used restricted maximum likelihood. Risk of bias and certainty were assessed using RoB2 and a conservative GRADE/CINeMA-informed framework.

**Results:**

The primary network included 12 studies, 25 analytic arms, and 491 participants; the broader sensitivity network included 14 studies, 29 arms, and 606 participants. Estimates versus control were 0.33 (95% CI −0.04 to 0.69) for conventional exercise, 0.64 (0.18 to 1.09) for Pilates, 0.69 (0.38 to 1.01) for technology-assisted exercise, and 0.69 (0.14 to 1.25) for technology-delivered Pilates. Applicability mapping identified direct older-adult community evidence (4 studies, 187 participants), aging-related clinical rehabilitation evidence (6 studies, 216 participants), and indirect non-geriatric evidence (2 studies, 88 participants). Certainty was low for technology-assisted exercise and very low for other active-versus-control comparisons.

**Conclusion:**

Active interventions generally favoured the intervention over control, but sparse evidence, heterogeneity, control variability, outcome-domain sensitivity, clinically fragile transitivity, and low/very low certainty preclude a definitive hierarchy. Findings may inform risk-stratified community programmes, home-based support, digital-equity planning, and implementation trials rather than justify a single preferred intervention, platform, or automated decision-support model.

**Systematic review registration:**

https://www.crd.york.ac.uk/PROSPERO/view/CRD420261383909, PROSPERO, CRD420261383909.

## 1 Introduction

Population aging has created an urgent public health need for scalable strategies that preserve mobility, support balance-oriented rehabilitation, and maintain functional independence among older adults and aging-related rehabilitation populations (1–3). Balance and postural-control impairments are closely related to mobility limitation, fall-risk vulnerability, rehabilitation demand, and loss of functional autonomy, but balance and postural-control outcomes should be distinguished from actual fall incidence or long-term functional-decline outcomes (1, 4). In older adults, balance impairment is rarely an isolated motor-performance problem. It often occurs within broader aging-related trajectories involving reduced neuromuscular reserve, sensory decline, multimorbidity, medication-related fall vulnerability, fear of falling, physical inactivity, reduced confidence, social participation restriction, and increasing care dependence (1, 4). These interacting vulnerabilities make balance-oriented exercise not only a rehabilitation issue but also a public health concern for community screening, risk stratification, preventive care planning, and maintenance of independence in later life. Because postural stability depends on the integration of neuromuscular strength, sensory feedback, trunk control, anticipatory postural adjustment, and motor coordination, exercise-based interventions remain central to balance-oriented rehabilitation and fall-risk-related care planning (5, 6). However, the growing diversity of exercise formats has made it increasingly difficult to determine how different intervention approaches compare across balance-, postural-control-, and fall-risk-related outcome domains.

Pilates has been increasingly investigated as a structured exercise approach for improving balance and postural-control performance (7–9). Pilates is distinguished by controlled trunk-centred movement, core stabilization, breathing coordination, postural alignment, movement precision, and body awareness, all of which provide a plausible neuromuscular rationale for improving postural stability (5, 10–12). Its low-impact and progressively controlled format may be particularly relevant for individuals requiring safe, structured, and motor-control-oriented training, including older adults and people with neurological or rehabilitation-related conditions (7, 10–12). From an aging-public-health perspective, these features may also be relevant to community-delivered or group-based programmes when instruction, progression, safety screening, and adherence support are feasible. Nevertheless, the Pilates evidence base remains heterogeneous in terms of populations, intervention protocols, comparator conditions, outcome measures, supervision, and intervention duration, limiting clear interpretation of its comparative value relative to other exercise-based approaches (7–9).

In parallel, emerging technology-assisted exercise—including immersive or non-immersive AR/VR, motion-sensing or exergaming formats, and remotely delivered digital exercise—has increasingly been used to provide task-specific, feedback-enriched balance and postural-control training (13–15). These approaches may extend conventional balance training through visual or auditory feedback, interactive task practice, sensorimotor engagement, remote or home-compatible delivery, and potentially improved motivation or adherence (13, 16, 17). For older adults, such formats may be particularly relevant when transport barriers, limited rehabilitation access, home-based care needs, or motivation challenges restrict participation in facility-based programmes. However, technology-assisted exercise is an umbrella category rather than a standardized intervention, and its effects may vary according to platform, task design, supervision level, comparator condition, outcome domain, target population, and implementation context (13–15). Its public health value also depends on implementation conditions such as device access, internet connectivity, digital literacy, usability, privacy, therapist or caregiver support, and cost (16, 17). Digital or sensor-supported delivery should not be equated with artificial intelligence; the presence of VR, AR, motion sensing, exergaming, or remote delivery does not by itself indicate automated decision support, adaptive personalization, or machine-learning-guided rehabilitation (18). In the included literature, technology-delivered Pilates primarily comprised Pilates-based exercise delivered through synchronous video, telerehabilitation, or other technology-mediated supervision rather than through algorithmically adaptive systems.

Existing reviews have generally focused on single intervention categories, specific populations, or conventional pairwise comparisons (7–9, 13–15). Although such evidence is useful for evaluating whether Pilates or technology-assisted exercise may improve balance-related outcomes, it does not fully clarify how Pilates, technology-assisted exercise, technology-delivered Pilates, conventional exercise, and control conditions relate to one another within a shared comparative framework (7–9, 13–15). Moreover, prior evidence syntheses have not sufficiently clarified which findings are directly applicable to community-dwelling older adults, which derive from aging-related clinical rehabilitation populations, and which remain indirect for older-adult public health planning. This distinction is important because intervention choice, supervision intensity, home-based feasibility, digital access, adverse-event monitoring, and referral needs may differ substantially between healthy older adults, frail or high-fall-risk older adults, and people with neurological or functional vulnerability. Direct head-to-head evidence among these intervention types remains limited, and conventional pairwise meta-analysis is not sufficient to characterize relative effects when the evidence base includes multiple partially connected intervention nodes (7–9, 13–15, 19, 20). Network meta-analysis can integrate direct and indirect evidence across such nodes, but its findings require cautious interpretation when comparisons are sparse, populations are clinically heterogeneous, intervention nodes are broad, control conditions are heterogeneous, and certainty of evidence is limited (19–21).

Based on prior pairwise evidence and the rehabilitation rationale for structured balance-oriented exercise, the review was guided by the directional expectation that active exercise-based interventions would generally yield favourable estimates versus control (2, 7, 13, 14). No superiority hierarchy among active intervention nodes was specified because the review was exploratory and substantial clinical, intervention, comparator, and outcome heterogeneity was anticipated (21, 22). Accordingly, any public health interpretation should be evidence-graded and implementation-oriented rather than ranking-driven.

Therefore, this systematic review and exploratory network meta-analysis aimed to compare Pilates, technology-assisted exercise, technology-delivered Pilates, conventional exercise, and control conditions for balance- and postural-control-related outcomes, with fall-related proxy evidence retained only in a broader randomized-trial sensitivity layer. The primary inferential layer was restricted to source-verified randomized trials with eligible balance or postural-control endpoints and compatible post-intervention arm-level data. The broader randomized-trial layer was used to examine directional sensitivity after adding broader-layer fall-related proxy or pilot evidence defined in the final analytical amendment before model rerunning and was not treated as confirmatory evidence or as a replacement for the primary analysis. We evaluated comparative estimates versus control, active-intervention comparisons, network structure, baseline comparability, control-node composition, concurrent physical-activity and co-intervention reporting, transitivity, heterogeneity and direct–indirect evidence, risk of bias, certainty of evidence, and reproducibility of the analytical workflow. In addition, we descriptively mapped technology modality, delivery characteristics, implementation reporting, and explicitly reported algorithmic features to clarify how the current evidence base relates to emerging technology-supported rehabilitation. To address the Aging and Public Health relevance of the review, we further mapped the aging-public-health applicability of the evidence base by distinguishing direct older-adult community evidence, aging-related clinical rehabilitation evidence, and indirect non-geriatric evidence, and by considering how supervision, delivery setting, technology access, safety monitoring, digital equity, and implementation context affect translation into community-based and targeted health-management strategies for older adults. Given the anticipated sparsity and clinical heterogeneity of the evidence base, ranking-related outputs were retained only as exploratory supplementary summaries and were not interpreted as confirmatory evidence of intervention superiority, clinical recommendation, public health policy recommendation, or a definitive treatment hierarchy (21, 22).

## 2 Methods

### 2.1 Review design, registration, and analytical framework

This systematic review and exploratory network meta-analysis was reported according to the PRISMA 2020 statement and the PRISMA extension for network meta-analyses (19, 23). The protocol was registered in PROSPERO before quantitative synthesis under the original title Pilates and AR/VR-Based Technology-Assisted Exercise for Improving Balance and Postural Control: A Systematic Review and Exploratory Network Meta-Analysis; PROSPERO CRD420261383909.

The registered title emphasized AR/VR-based technology-assisted exercise. During evidence mapping and source verification, the technology-assisted exercise domain was operationalized as a broader digitally supported exercise node that included AR/VR, exergaming, Kinect or motion-sensing systems, active video-game exercise, telerehabilitation, and other structured technology-supported exercise formats (13–17). This broadening was treated as a protocol-relevant scope deviation and operational broadening, rather than as evidence that these modalities were clinically interchangeable. It did not introduce a new population, outcome domain, comparator class, or *post hoc* efficacy-driven intervention node.

An AR/VR-only five-node network was not feasible because the retrieved AR/VR evidence did not connect all planned intervention nodes. We therefore retained the broader technology-assisted exercise category captured by the original search strategy, treated this decision as a post-registration scope deviation, and evaluated an AR/VR-only reduced network as a feasibility analysis. This decision was made to preserve an interpretable connected evidence structure and was not based on the magnitude or direction of observed effect estimates. Technology-delivered Pilates was coded separately as Pilates-based exercise delivered through remote, digital, telecommunication, video, or other technology-mediated platforms.

The review compared five intervention or comparator nodes: control, conventional exercise, Pilates, technology-assisted exercise, and technology-delivered Pilates. Pilates referred to Pilates-based exercise delivered without digital, remote, or technology-mediated delivery. Conventional exercise referred to structured non-technology-assisted exercise, physical therapy, balance training, core-stability exercise, yoga/Otago-type training, or conventional rehabilitation without a Pilates-specific or digital-delivery component. Control referred to no active exercise, waitlist/no intervention, usual activity or usual care without structured exercise, education-only or minimal-contact control, or other non-exercise comparator conditions. Comparator content was source-verified, and arms containing structured exercise, physical therapy, balance training, or active rehabilitation were classified as conventional exercise rather than control.

Following source verification of allocation procedures, the main inferential network was restricted to source-verified randomized controlled trials. One controlled study previously considered for quantitative synthesis was identified as using first-come, first-served allocation and was therefore retained only as contextual nonrandomized controlled evidence outside the randomized-trial networks. Where more than one randomized arm within the same trial represented the same network node, same-node arms were combined before contrast construction. In Lee and Yoo 2017, the Yoga and Self-Otago arms were both classified as conventional exercise and were combined into one conventional-exercise analytic arm before pairwise effect-size construction.

Two randomized-trial evidence layers were defined. The primary randomized-trial network was the main inferential layer and included source-verified randomized trials reporting eligible balance or postural-control endpoints with extractable or derivable post-intervention arm-level data. The broader randomized-trial network additionally retained fall-related proxy or pilot/broader evidence defined in the final analytical amendment before model rerunning and was used only to examine directional sensitivity after adding broader randomized evidence. It did not replace the primary analysis or serve as confirmatory evidence. Detailed PICOS eligibility criteria, evidence-layer allocation rules, intervention-node definitions, and outcome hierarchy are provided in Supplementary Table S2. Study-design verification and risk-of-bias-tool compatibility are reported in Supplementary Table S5, intervention-node coding in Supplementary Table S8, technology-modality mapping in Supplementary Table S9, sensitivity and analytical-fragility analyses in Supplementary Table S17, and protocol concordance and deviations in Supplementary Table S22. To strengthen aging-public-health interpretation, we additionally conducted a descriptive applicability and implementation mapping that distinguished direct older-adult community evidence, aging-related clinical rehabilitation evidence, and indirect non-geriatric evidence.

### 2.2 Search strategy, record management, study selection, and missing-data handling

Searches were conducted in PubMed/MEDLINE, Embase, Web of Science Core Collection, Cochrane Library/CENTRAL, ClinicalTrials.gov, CINAHL Complete, APA PsycInfo, and Scopus using structured database- and platform-specific strategies consistent with systematic-review search guidance (24). All database and register searches were completed on 2 May 2026. No restrictions on language, publication year, publication status, document type, peer-review status, full-text availability, or access status were applied at the search stage. The complete search strategies, search dates, information sources, record counts, and source-level audit information are provided in Supplementary Table S1.

Retrieved records were exported, deduplicated, and imported into the screening library. Title/abstract screening and full-text retrieval were managed through Rayyan and a closed PRISMA audit trail to preserve traceability from record identification to final evidence allocation (25). One reviewer conducted the initial title/abstract screening and full-text eligibility assessment, and a second reviewer verified eligibility decisions against the locked eligibility criteria. This was an author/verifier workflow rather than independent duplicate screening. Uncertain or discrepant cases were resolved through consensus discussion before final study allocation. This workflow and its deviation from fully independent duplicate screening were treated as a transparent reporting issue rather than as independent duplicate review.

Full-text exclusions were assigned using hierarchical exclusion reasons to avoid double counting. Study allocation across the primary randomized-trial NMA, broader randomized-trial NMA, contextual evidence, nonrandomized controlled evidence, and missing-data exclusions was finalized before network estimation. Reports were not entered into effect-size estimation when compatible post-intervention arm-level n, mean, and SD, or numerical information sufficient to derive compatible values without unverifiable assumptions, were unavailable for the prioritized eligible outcome.

Reports without compatible quantitative input were rechecked using the full text, tables, figures, supplementary materials, and reported statistical summaries where available. Missing post-intervention means or standard deviations were not imputed using unverifiable assumptions (26). Study authors were not contacted for missing numerical data, and no unpublished arm-level numerical data were incorporated into the reported analyses. Reports that were relevant to the review question but lacked compatible quantitative input were retained in the full-text audit trail and, where appropriate, used only for qualitative or contextual synthesis. The PRISMA allocation audit and full-text decision log are provided in Supplementary Tables S3, S4. Missing-data derivation attempts, author-contact status, and final synthesis handling are documented in Supplementary Table S4B.

### 2.3 Data extraction, outcome harmonization, baseline and concurrent-activity audits, and Hedges’ g effect-size construction

Arm-level data were extracted for study identifiers, population characteristics, intervention and comparator nodes, intervention dose and supervision, outcome domains, selected endpoint, sample size, post-intervention means, post-intervention standard deviations, measurement direction, assessment time point, source table or figure, and analysis-layer allocation (26–28). Where available, baseline values for the selected outcome were also extracted for baseline-comparability auditing. One reviewer conducted the initial data extraction, and a second reviewer verified extracted values, intervention-node coding, outcome-domain coding, endpoint prioritization, direction-of-effect harmonization, baseline-audit entries, same-node arm combination, and analysis-layer allocation before dataset locking. Study-level characteristics and the locked arm-level extraction dataset are provided in Supplementary Tables S6, S7.

Because included studies used different balance and postural-control measures, outcome-direction harmonization was performed before effect-size construction (29). Positive Hedges’ g values were defined to represent better balance-, postural-control-, or fall-related performance. For lower-is-better outcomes, means were sign-reversed before effect-size construction, while standard deviations were unchanged. The outcome hierarchy prioritized clinical or field-based balance-performance outcomes, followed by instrumented or laboratory-based postural-control outcomes. Fall-related proxy outcomes were retained only in the broader randomized-trial sensitivity layer and did not replace the primary balance or postural-control inference. Outcome selection, hierarchy, and direction-harmonization decisions are provided in Supplementary Table S12.

When multiple post-intervention time points were available, the immediate post-intervention assessment was used. Pairwise standardized effects were calculated as Hedges’ g using harmonized post-intervention arm-level n, mean, and SD, pooled post-intervention within-arm standard deviations, and exact small-sample correction implemented in meta:metacont with method.smd = “Hedges” and exact.smd = TRUE. Standard errors were derived consistently for all direct contrasts before network estimation. Where more than one arm within the same randomized trial represented the same network node, same-node arms were combined before contrast construction using standard arm-combination rules (30).

The post-intervention arm-level approach was used because adjusted between-group estimates and change-score standard deviations were not consistently available across studies (31). This approach may be sensitive to chance baseline imbalance in small trials. Therefore, baseline values for the selected outcome were summarized using descriptive standardized baseline differences where available, and potentially consequential selected-outcome baseline imbalance was examined in a baseline-imbalance exclusion sensitivity analysis where network connectivity permitted. This baseline audit was not interpreted as a baseline-adjusted NMA.

To address potential confounding from non-intervention activity and co-interventions, we additionally extracted whether participants were instructed to maintain habitual physical activity, whether additional structured exercise or rehabilitation was restricted, whether non-intervention activity was monitored, and whether co-interventions or contamination were reported. These variables were summarized in the control-node and concurrent-activity audit and were considered in the structured transitivity assessment, RoB2 interpretation, and certainty assessment. Pairwise Hedges’ g construction is reported in Supplementary Table S13.

### 2.4 Technology modality, delivery characteristics, and explicitly reported algorithmic features

Technology mapping was descriptive and was not used to define efficacy nodes, reclassify comparators, modify eligibility, estimate treatment effects, or infer treatment rankings (32). For each eligible intervention arm, we extracted technology modality, delivery setting, supervision mode, feedback or cueing functions, motion-tracking or interaction features, remote-delivery format, home-based feasibility, adherence reporting, adverse-event reporting, usability or acceptability reporting, technology access requirements, digital literacy considerations, cost reporting, therapist involvement, follow-up durability, and equity-relevant considerations.

We also recorded whether each intervention explicitly reported automated decision support, machine-learning prediction, automated risk stratification, algorithm-guided progression, adaptive personalization, or comparable algorithmic decision-making. Sensor-derived measures, motion-tracking outputs, and remote-monitoring dashboards were recorded separately as digital monitoring or implementation features and were not treated as algorithmic decision-support features unless the source report explicitly described automated decision-making, prediction, adaptation, or personalized progression. Standard VR, AR, Kinect, motion sensing, exergaming, active video-game exercise, telerehabilitation, or remote video delivery was not classified as algorithmically supported care unless such features were explicitly described in the source report (18). Where these features were absent or unclear, the intervention was classified as digitally supported or technology-assisted exercise rather than as automated or algorithm-guided rehabilitation.

These variables were summarized descriptively to clarify technology modality, implementation relevance, and evidence gaps for emerging technology-supported rehabilitation. They were not used to support platform-specific efficacy claims, automated decision-support efficacy, adaptive-personalization effects, intervention procurement recommendations, or treatment-ranking interpretations. Study-arm-level technology modality and explicitly reported algorithmic-feature mapping are provided in Supplementary Table S9.

### 2.5 Aging-public-health applicability and implementation mapping

To strengthen aging-public-health interpretation and clarify the relevance of the evidence base for older-adult care, fall-risk management, and digitally supported rehabilitation pathways, we conducted a descriptive aging-public-health applicability and implementation mapping in parallel with the network meta-analysis (1, 16, 17). This mapping was not used to define efficacy nodes, modify intervention coding, change effect-size estimation, alter network geometry, or generate treatment rankings. Instead, it was used to interpret applicability, implementation relevance, and evidence boundaries for older-adult care and aging-related rehabilitation contexts.

Included studies were classified into three applicability categories. Direct older-adult community evidence referred to studies enrolling community-dwelling older adults, older women, healthy older adults, or comparable older-adult populations. Aging-related clinical rehabilitation evidence referred to studies enrolling participants with neurological or functional-vulnerability conditions relevant to older-adult rehabilitation and fall-risk management, including Parkinson disease, chronic stroke, multiple sclerosis, or comparable rehabilitation populations. Indirect non-geriatric evidence referred to studies in younger, athletic, healthy sedentary, or otherwise non-older-adult populations whose findings may inform mechanisms, intervention feasibility, or indirect comparative evidence but should not be generalized directly to older-adult public health practice.

For each study and intervention node, implementation-relevant characteristics were extracted or coded where reported, including delivery setting, supervision mode, home-based or remote-delivery feasibility, technology access requirements, digital-literacy considerations, therapist or caregiver involvement, adherence reporting, safety or adverse-event reporting, usability or acceptability reporting, cost reporting, privacy considerations, and equity-relevant barriers (16, 17, 32). These variables were summarized descriptively using the locked extraction tables, technology-modality mapping, control-node audit, and structured transitivity assessment.

The aging-public-health applicability mapping was used to guide interpretation of public health practice, community-based intervention design, targeted health management, digital-equity considerations, and referral boundaries for older adults. It was not interpreted as a separate efficacy analysis, subgroup meta-analysis, or basis for policy, procurement, or programme adoption of a specific exercise format, digital platform, AR/VR device, or automated decision-support model (18, 32).

### 2.6 Network meta-analysis, transitivity assessment, and sensitivity analyses

Network meta-analysis was conducted in R version 4.6.0 using a frequentist framework implemented with the netmeta package (20, 33). Hedges’ g values with 95% confidence intervals were used as the effect metric. The random-effects model was used as the primary model because clinical and methodological heterogeneity was expected across populations, intervention formats, comparators, technology modalities, supervision settings, and outcome measures. Restricted maximum likelihood was used to estimate between-study variance. A common-effect model was retained only as a modelling sensitivity analysis. Multi-arm trial correlations were handled within the network model rather than by treating correlated contrasts as independent.

The control node was used as the reference for the main comparative estimates. The primary randomized-trial NMA was treated as the main inferential synthesis layer. The broader randomized-trial network was used only to examine directional sensitivity after adding broader-layer randomized evidence and did not replace the primary inference. Comparative effects were interpreted primarily versus control, while active-intervention comparisons were interpreted cautiously according to effect estimates, confidence intervals, network geometry, direct-evidence structure, transitivity considerations, and certainty of evidence. League tables and P-score summaries were retained only as exploratory supplementary outputs displayed in fixed conceptual order and were not interpreted as evidence of superiority, certainty, clinical recommendation, technology procurement guidance, or a definitive treatment hierarchy (22).

Transitivity was evaluated structurally rather than assumed from statistical heterogeneity tests. Candidate effect modifiers included population and pathology, age or life-stage context, mechanism of balance impairment, baseline balance or fall-risk status, intervention modality, technology or delivery format, intervention dose and supervision, setting, comparator intensity, control subtype, concurrent physical activity or co-intervention reporting, outcome domain, and assessment timing. Control conditions were audited because waitlist/no intervention, usual activity or usual care, education-only or minimal-contact control, and active non-exercise comparator content may not be clinically equivalent. Conditions containing structured exercise, physical therapy, balance training, or active rehabilitation were classified as conventional exercise rather than control. Because the network was small and clinically heterogeneous, formal network meta-regression was not considered reliable. Transitivity was considered plausible only at a broad balance-oriented exercise-category level and clinically fragile. The structured transitivity assessment is provided in Supplementary Table S11.

Sensitivity and analytical-fragility analyses examined the broader randomized-trial network, exclusion of the study with potentially consequential selected-outcome baseline imbalance, non-exercise/minimal-intervention control sensitivity, exclusion of the active non-exercise Pilates-control study, exclusion of the athlete/low-back-pain study, restriction to clinical or older-rehabilitation populations, restriction to clinical or field-based balance-performance outcomes, merging of Pilates-related nodes, common-effect modelling, and an AR/VR-only feasibility analysis where network connectivity allowed. These analyses were interpreted as structural, applicability, and analytical-fragility checks rather than as proof of robustness. The AR/VR-only analysis was treated as a reduced-network feasibility check and was not used to replace the primary five-node technology-assisted exercise framework. Sensitivity analyses were interpreted according to point estimates, confidence intervals, network geometry, direct-evidence structure, and certainty implications rather than binary statistical significance. Sensitivity analyses are summarized in Supplementary Table S17.

Global heterogeneity and design-level inconsistency were assessed where estimable using network heterogeneity statistics, Q decomposition, and design-by-treatment diagnostics. Direct, indirect, network, and direct–indirect estimates were extracted for estimable comparisons. The random-effects contribution matrix was used to identify which direct comparisons contributed to key network estimates and to support risk-of-bias, indirectness, incoherence, and certainty judgments. Absence of a statistically clear heterogeneity or inconsistency signal was not interpreted as evidence of clinical homogeneity or consistency because the network was small and sparse. Direct–indirect evidence, inconsistency diagnostics, and contribution-matrix summaries are reported in Supplementary Table S18. Comparison-adjusted funnel plots were used only as descriptive visual assessments of potential small-study or reporting-bias signals because the evidence network contained few direct contrasts and sparse comparisons.

### 2.7 Risk of bias, certainty of evidence, and reproducibility

Risk of bias was assessed using the revised Cochrane risk-of-bias tool for randomized trials (RoB2) for all 14 source-verified randomized trials contributing to either randomized-trial evidence layer. Twelve trials contributed to the primary randomized-trial network, and two contributed only to the broader randomized-trial sensitivity network. Judgments were made across the standard RoB2 domains: bias arising from the randomization process, bias due to deviations from intended interventions, bias due to missing outcome data, bias in measurement of the outcome, and bias in selection of the reported result. One reviewer conducted the initial RoB2 assessment, and a second reviewer verified all domain-level and overall judgments against the source reports and the locked extraction dataset. Disagreements or unclear judgments were resolved through consensus discussion before the final certainty assessment.

**Figure 1:**
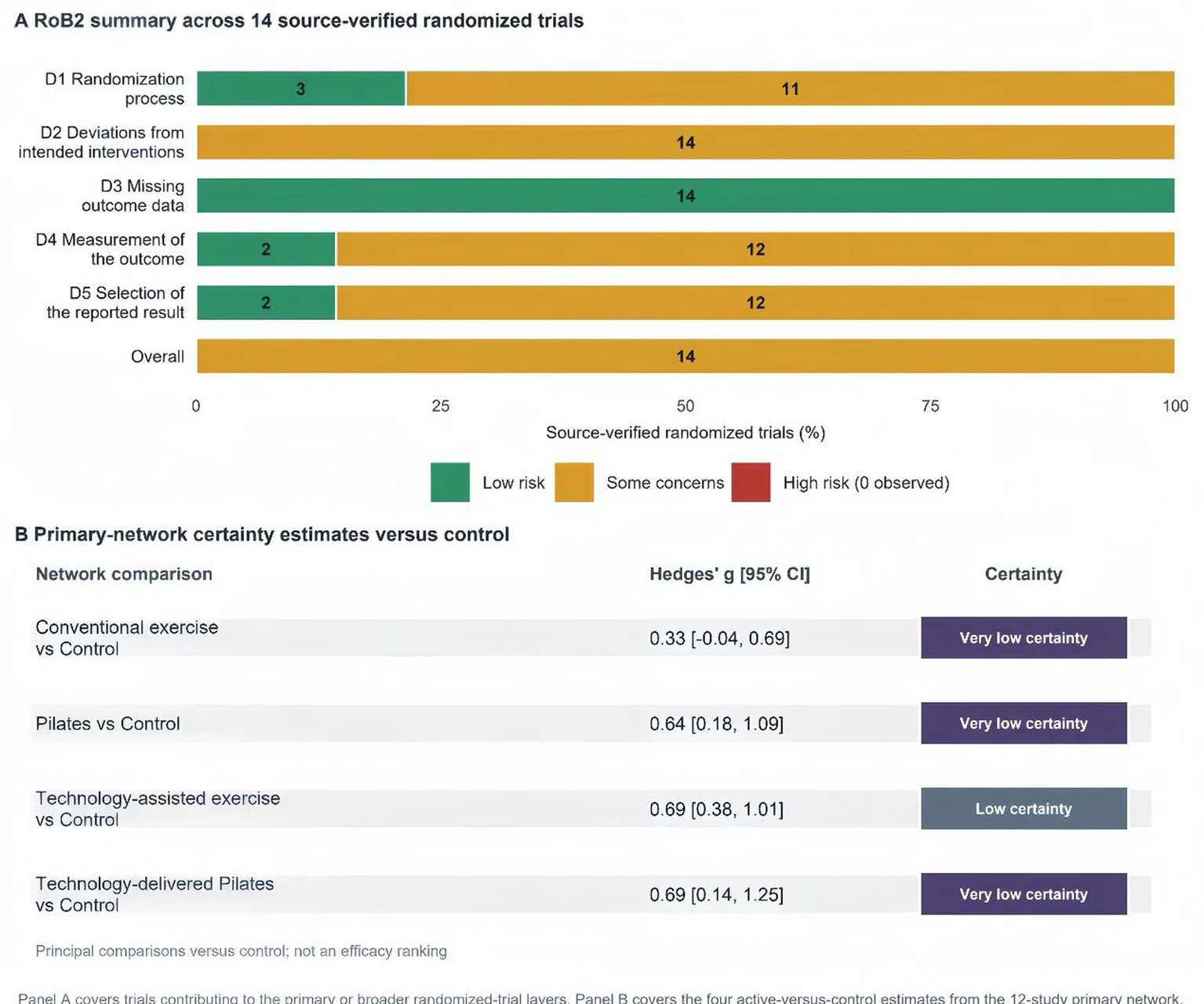
Risk of bias across all source-verified randomized trials and certainty of evidence for the primary randomized-trial network meta-analysis. **(A)** summarizes RoB2 domain-level and overall judgments across all 14 source-verified randomized trials contributing to either randomized-trial evidence layer, comprising 12 trials in the primary network and 2 trials included only in the broader randomized-trial sensitivity network. Numbers within the bars indicate trial counts, whereas the horizontal axis presents percentages. **(B)** Presents the four principal active-versus-control comparisons from the 12-study primary randomized-trial network, including Hedges’ g estimates, 95% confidence intervals, and comparison-level certainty ratings. The two broader-only randomized trials did not contribute to the primary-network certainty ratings shown in **(B)**. Certainty ratings were used to contextualize the primary estimates and should not be interpreted as an efficacy ranking.

The controlled study identified during source verification as using nonrandom first-come, first-served allocation was retained outside both randomized-trial networks. It was not assessed using RoB2 for randomized-trial inference and did not contribute to the randomized-trial certainty assessments. Study-design verification and risk-of-bias-tool compatibility are reported in Supplementary Table S5. Study-level RoB2 judgments for all 14 source-verified randomized trials are reported in Supplementary Table S19 and Supplementary Figure S5, and their domain-level distribution is summarized in Figure 1A.

Certainty of evidence was assessed at the comparison level using a conservative GRADE/CINeMA-informed framework (21, 34). Randomized-trial evidence started at high certainty. For each comparison, individual domains were assigned zero, one, or two levels of downgrading for no serious concern, serious concern, or very serious concern, respectively. The assessment considered within-study risk of bias, reporting-bias and data-availability concerns, indirectness, imprecision, heterogeneity, incoherence, and the contribution of direct evidence to each network estimate.

To avoid double counting, differences in population and pathology, intervention-node breadth, technology-platform heterogeneity, control-node variability, outcome-domain sensitivity, and clinically fragile transitivity were considered collectively under indirectness. Sparse direct evidence, small sample sizes, confidence-interval width, and overlap with the analytical reference range were considered under imprecision. Direct–indirect disagreement was considered under incoherence. Clinical heterogeneity was not additionally downgraded under statistical heterogeneity when the same concern had already been captured under indirectness. Review-wide data-availability concerns resulted in an additional comparison-specific downgrade only when they could be linked directly to the comparison being assessed.

An analytical reference range of −0.30 to +0.30 Hedges’ g was used solely to support the assessment of imprecision across heterogeneous standardized outcomes. This range was not treated as a validated minimal clinically important difference, a clinical-equivalence margin, or a threshold for clinical importance. Certainty ratings were not used to validate P-score summaries, infer treatment rankings, or identify a best intervention. For comparisons involving technology-assisted exercise or technology-delivered Pilates, platform heterogeneity, delivery-mode variability, sparse evidence, and the absence of consistently reported automated decision-support or adaptive-personalization features were considered when judging indirectness and applicability.

Because the network was small and clinically heterogeneous, certainty was downgraded conservatively. Favourable point estimates were not considered sufficient evidence of high certainty when direct evidence was sparse, confidence intervals were wide or overlapping, control conditions varied, outcome-domain sensitivity was present, or local direct–indirect disagreement was identified. Domain-specific downgrade levels, rules used to avoid double counting, and final certainty ratings are reported in Supplementary Table S20. Figure 1B summarizes the four principal active-versus-control certainty assessments from the 12-study primary randomized-trial network.

**Figure 2:**
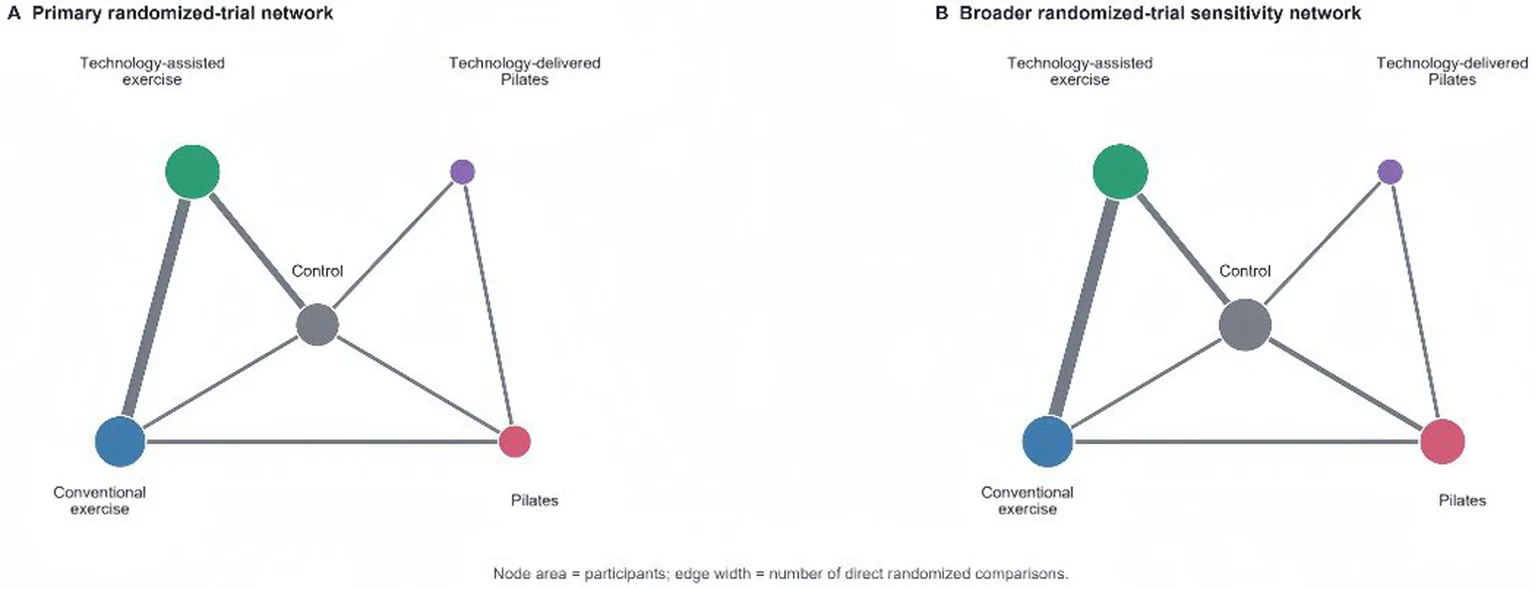
Network geometry of the primary and broader randomized-trial network meta-analyses. **(A)** Shows the primary randomized-trial network, which included 12 studies, 26 randomized source arms represented as 25 analytic arms after same-node arm combination, and 491 participants. **(B)** Shows the broader randomized-trial sensitivity network, which included 14 randomized studies, 30 randomized source arms represented as 29 analytic arms, and 606 participants. Nodes represent intervention or comparator categories; node area is proportional to the number of participants contributing to each node, and edge width is proportional to the number of direct randomized comparisons between nodes. Node size and edge width should not be interpreted as effect size, certainty of evidence, or intervention ranking.

**Figure 3:**
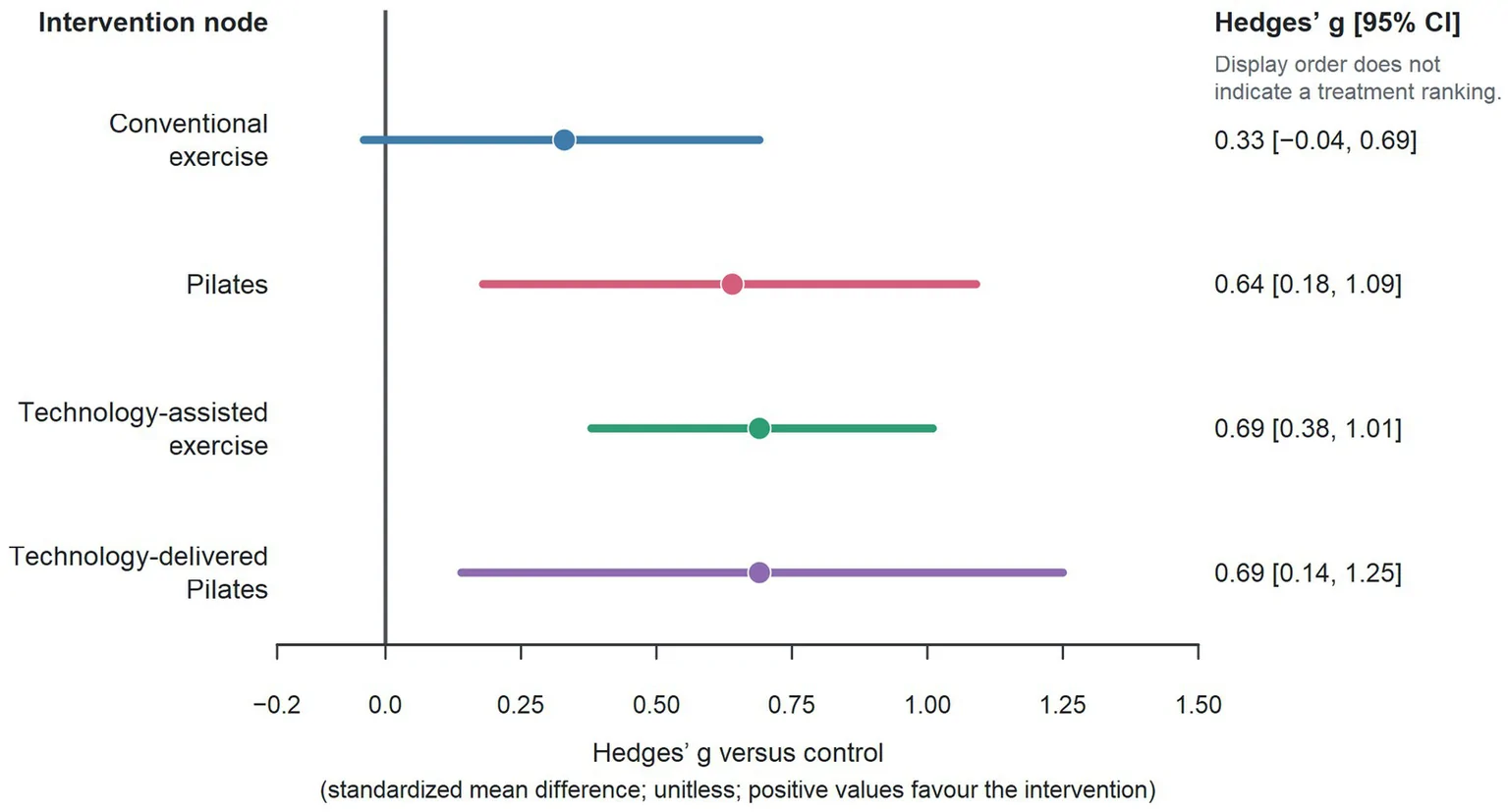
Primary randomized-trial NMA estimates versus control. Hedges’ *g* values are shown for active intervention nodes compared with control in the primary randomized-trial network. Positive values favour the intervention after outcome-direction harmonization. Error bars represent 95% confidence intervals. Display order is not a treatment ranking. Certainty ratings are reported separately in Figure 1B and Supplementary Table S20. The primary randomized-trial network included 12 studies, 25 analytic arms, and 491 participants.

**Figure 4:**
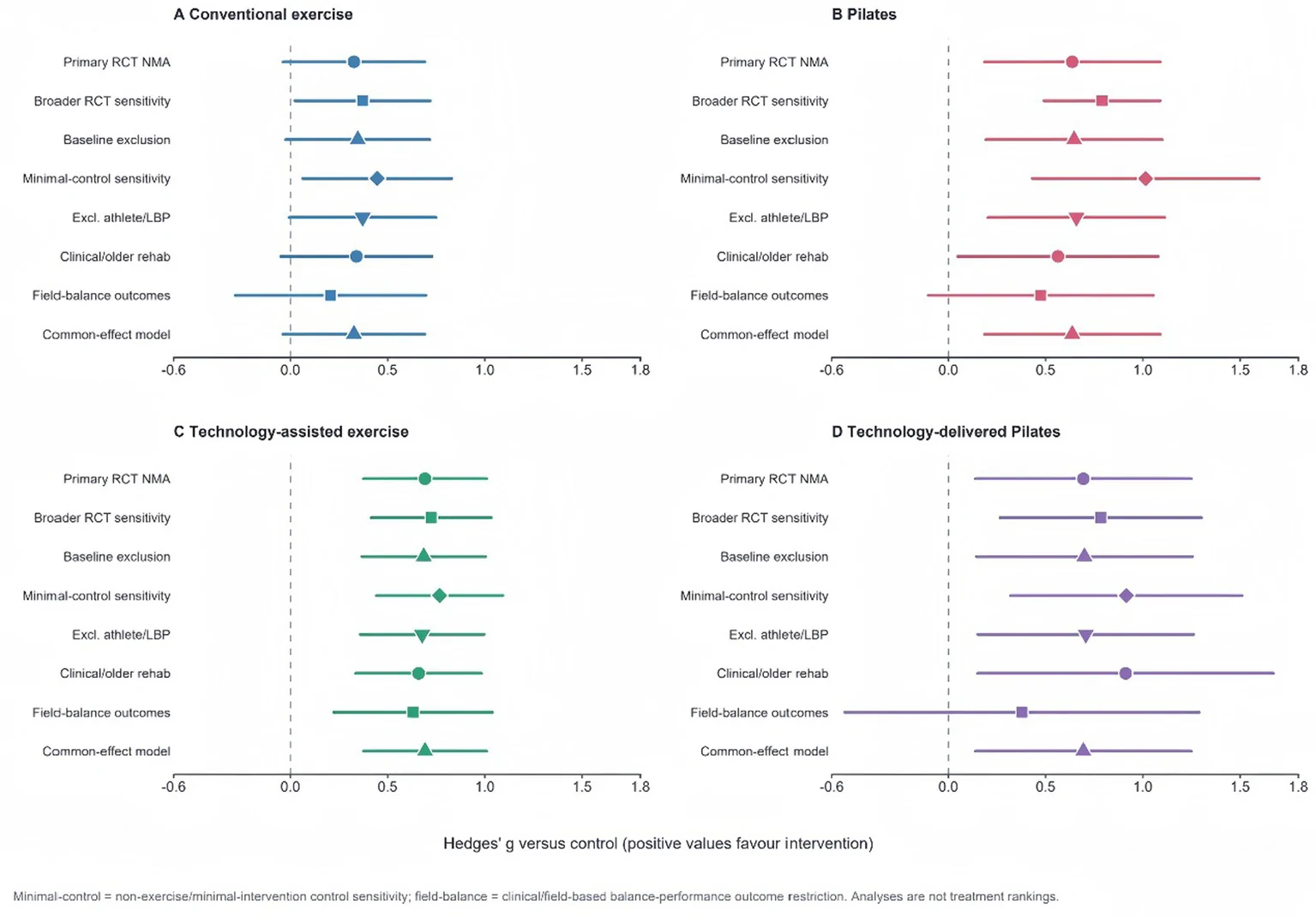
Sensitivity and analytical-fragility analyses for estimates versus control: **(A)** conventional exercise; **(B)** Pilates; **(C)** technology-assisted exercise; and **(D)** technology-delivered Pilates. Hedges’ *g* values are shown for each active intervention node compared with control across the primary randomized-trial NMA and sensitivity analyses. Positive values favour the intervention after outcome-direction harmonization. Error bars represent 95% confidence intervals. Sensitivity analyses were interpreted according to effect estimates, confidence intervals, network geometry, direct-evidence structure, and certainty implications rather than binary statistical significance. The broader randomized-trial network was a sensitivity evidence layer and did not replace the primary inference. Full sensitivity estimates, node-merging analysis, AR/VR-only feasibility analysis, direct–indirect diagnostics, and contribution-matrix summaries are provided in Supplementary Tables S17, S18.

**Figure 5:**
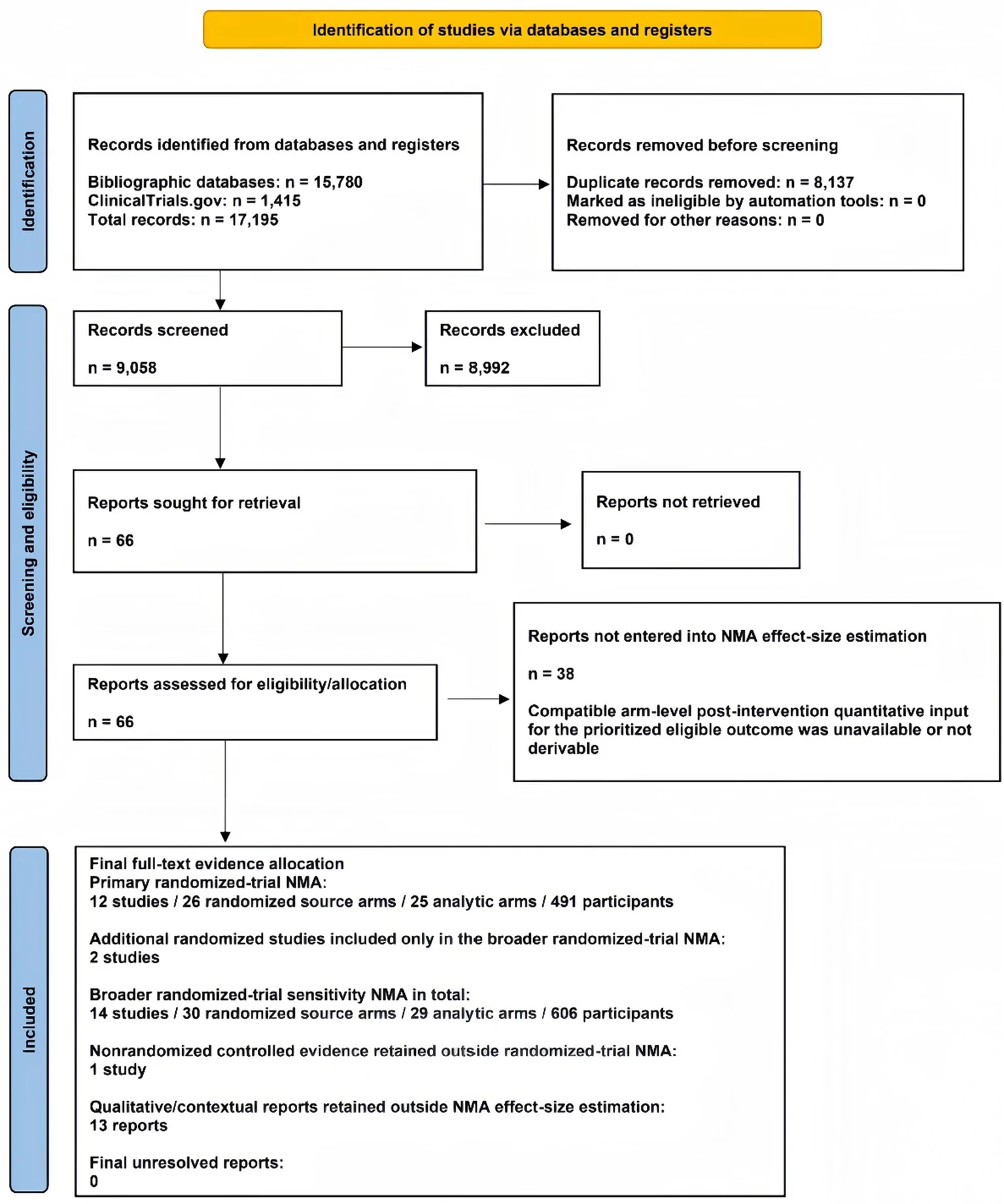
PRISMA flow diagram of study selection and final evidence allocation. Database and register searches identified 17,195 records, including 15,780 records from bibliographic databases and 1,415 records from ClinicalTrials.gov. After removal of 8,137 duplicate records, 9,058 records were screened by title and abstract, and 8,992 records were excluded. Sixty-six full-text reports were sought and retrieved; no report was unavailable. All 66 full-text reports were assessed and assigned a final eligibility or allocation decision. Thirty-eight reports were not entered into NMA effect-size estimation because compatible arm-level post-intervention quantitative input for the prioritized eligible outcome was unavailable or not derivable. After source verification, the primary randomized-trial NMA included 12 studies, 26 randomized source arms represented as 25 analytic arms after same-node arm combination, and 491 participants. The broader randomized-trial sensitivity network included 14 randomized studies, 30 randomized source arms represented as 29 analytic arms, and 606 participants. One nonrandomized controlled study was retained outside the randomized-trial networks as contextual evidence, and 13 additional reports were retained for qualitative or contextual synthesis. No report remained unresolved after final full-text assessment.

A self-contained reproducibility package was submitted as supporting material. It contains the de-identified analysis-ready input data, the exact final statistical analysis script, final R scripts for Figures 1–4 and Supplementary Figures S1–S5, the editable Figure 5 Word/PDF/JPG source files, figure-source datasets, final output tables, an execution log, and software-session information. The analytical workflow uses relative paths and generates the reported outputs directly from the supplied input data. The package was tested from a newly created directory in a fresh software session and reproduced the primary and broader network estimates, sensitivity analyses, heterogeneity and inconsistency diagnostics, direct–indirect results, contribution outputs, tables, regenerated Figures 1–4 and Supplementary Figures S1–S5 as PDFs from the supplied R scripts, and assembled Figure 5 from the supplied final Figure 5 source files. Copyright-restricted full-text source-audit materials were not included in the reproducibility package and were retained only for confidential editorial verification.

## 3 Results

### 3.1 Study selection and evidence allocation

The study-selection process is summarized in Figure 5 and Supplementary Tables S1–S4B. Database and register searches identified 17,195 records, including 15,780 bibliographic database records and 1,415 ClinicalTrials.gov records. After removal of 8,137 duplicate records, 9,058 records were screened by title and abstract, of which 8,992 were excluded. Sixty-six full-text reports were sought and retrieved; no report was unavailable.

All 66 full-text reports were assessed and assigned a final eligibility or allocation decision. Thirty-eight reports were not entered into NMA effect-size estimation because compatible arm-level post-intervention quantitative input for the prioritized eligible outcome was unavailable or could not be derived without unverifiable assumptions. These reports were retained in the full-text audit trail. Study authors were not contacted for missing numerical data, and no unpublished arm-level numerical data were incorporated.

After source verification, 14 randomized studies contributed to at least one randomized-trial NMA layer. Twelve randomized studies entered the primary randomized-trial network, and two additional randomized studies entered only the broader randomized-trial sensitivity network. One controlled study using nonrandom first-come, first-served allocation was retained outside the randomized-trial networks as contextual nonrandomized controlled evidence. Thirteen additional reports were retained for qualitative or contextual synthesis. No report remained unresolved after final full-text assessment.

### 3.2 Study characteristics, node coding, outcome harmonization, and network geometry

Table 1 summarizes the source-verified randomized studies contributing quantitative data and their allocation to the primary and broader randomized-trial network layers, with contextual nonrandomized controlled evidence shown separately. The primary randomized-trial NMA included 12 studies, 26 randomized source arms represented as 25 analytic arms after same-node arm combination, and 491 participants. The broader randomized-trial sensitivity network included these 12 studies plus two additional broader-layer randomized studies, resulting in 14 studies, 30 randomized source arms represented as 29 analytic arms, and 606 participants.

**Table 1:** Characteristics and evidence-layer allocation of source-verified randomized studies contributing quantitative data, with contextual nonrandomized controlled evidence shown separately.

Study	Population / condition	Source-verified design	Participants, *n*	Source / analytic arms	Intervention–comparator node(s)	Outcome (domain)	Evidence-layer allocation
Abdelraouf et al. (44)	Athletes with nonspecific low back pain	Randomized clinical trial	50	2/2	Technology-assisted exercise vs. conventional exercise	SEBT posteromedial reach (balance performance)	Primary and broader randomized-trial NMA
Bacha et al. (45)	Community-dwelling older adults	Randomized clinical trial	46	2/2	Technology-assisted exercise vs. conventional exercise	Mini-BESTest (balance performance)	Primary and broader randomized-trial NMA
Duff et al. (46)	People with multiple sclerosis	Randomized controlled trial	30	2/2	Pilates vs. control	FABS (balance performance)	Primary and broader randomized-trial NMA
Eldemir et al. (47)	People with multiple sclerosis	Randomized controlled trial	30	2/2	Technology-delivered Pilates vs. control	LOS (instrumented/laboratory postural control)	Primary and broader randomized-trial NMA
Lazarowitz Zanzuri et al. (48)	Healthy sedentary women	Randomized controlled intervention	38	2/2	Technology-delivered Pilates vs. Pilates	Stork test (balance performance)	Primary and broader randomized-trial NMA
Junata et al. (49)	Chronic stroke survivors	Assessor-blinded randomized controlled trial	30	2/2	Technology-assisted exercise vs. conventional exercise	BBS (balance performance)	Primary and broader randomized-trial NMA
Kalron et al. (50)	People with multiple sclerosis	Assessor-blinded randomized controlled trial	45	2/2	Pilates vs. conventional exercise	BBS (balance performance)	Primary and broader randomized-trial NMA
Lee and Yoo (51)	Older women	Three-arm randomized controlled trial	30	3/2	Technology-assisted exercise vs. conventional exercise	EO CoP-x (instrumented/laboratory postural control)	Primary and broader randomized-trial NMA
Lee et al. (52)	Community-dwelling older adults	Single-blind randomized controlled trial	57	2/2	Technology-assisted exercise vs. control	BBS (balance performance)	Primary and broader randomized-trial NMA
Molhemi et al. (53)	People with multiple sclerosis	Single-blind randomized controlled trial	39	2/2	Technology-assisted exercise vs. conventional exercise	BBS (balance performance)	Primary and broader randomized-trial NMA
Sato et al. (54)	Healthy older adults	Randomized controlled trial	54	2/2	Technology-assisted exercise vs. control	FRT (balance performance)	Primary and broader randomized-trial NMA
Yen et al. (55)	People with Parkinson disease	Single-blinded randomized controlled trial	42	3/3	Technology-assisted exercise, conventional exercise, and control	SOT-6 single-task equilibrium score (instrumented/laboratory postural control)	Primary and broader randomized-trial NMA
Aibar-Almazan et al. (56)	Community-dwelling older women	Randomized controlled trial	107	2/2	Pilates vs. control	FES-I (fall-related proxy)	Broader randomized-trial NMA only
Prasertsakul et al. (57)	Healthy adults	Randomized pilot/preliminary study	8	2/2	Technology-assisted exercise vs. conventional exercise	CoP sway area, eyes closed (instrumented/laboratory postural control)	Broader randomized-trial NMA only
Sapi et al. (58)	Older adults	Nonrandomized controlled intervention; first-come, first-served allocation	53	NA	Technology-assisted exercise vs. conventional exercise	LOS movement velocity (postural control)	Contextual nonrandomized controlled evidence; outside both randomized-trial NMA layers

BBS, Berg Balance Scale; CoP, center of pressure; EO CoP-x, eyes-open center-of-pressure displacement in the x-axis; FABS, Fullerton Advanced Balance Scale; FES-I, Falls Efficacy Scale-International; FRT, Functional Reach Test; LOS, limits of stability; Mini-BESTest, Mini Balance Evaluation Systems Test; NA, not applicable; NMA, network meta-analysis; SEBT, Star Excursion Balance Test; SOT, Sensory Organization Test. Source/analytic arms are reported as original randomized source arms/analytic arms after same-node arm combination. The primary randomized-trial NMA included 12 studies, 26 source arms represented as 25 analytic arms, and 491 participants. The broader randomized-trial NMA was a sensitivity evidence layer and included all 12 primary studies plus Aibar-Almazan et al. (56) and Prasertsakul et al. (57), for 14 studies, 30 source arms represented as 29 analytic arms, and 606 participants. In Lee and Yoo (51), the Yoga and Self-Otago arms were combined as one conventional-exercise analytic arm before contrast construction. Sapi et al. (2019) used first-come, first-served allocation and was retained only as contextual nonrandomized controlled evidence; its 53 participants were not included in the randomized-trial NMA totals.

Across the randomized-trial evidence base, study populations included community-dwelling older adults, healthy or sedentary adults, athletes with nonspecific low back pain, and clinical groups such as people with multiple sclerosis, chronic stroke, and Parkinson disease. The same five intervention or comparator nodes were used across the primary and broader randomized-trial networks: control, conventional exercise, Pilates, technology-assisted exercise, and technology-delivered Pilates. One study contributed two same-node conventional-exercise source arms, which were combined into one conventional-exercise analytic arm before contrast construction.

Outcomes included clinical or field-based balance-performance tests and instrumented or laboratory-based postural-control outcomes in the primary randomized-trial network. Fall-related proxy evidence was retained only in the broader randomized-trial sensitivity layer. After direction harmonization, positive Hedges’ g values consistently indicated better balance-, postural-control-, or fall-related performance. Study characteristics, arm-level extraction, intervention-node coding, control-node audit, transitivity assessment, outcome-source decisions, direction harmonization, and pairwise Hedges’ g construction are provided in Supplementary Tables S6–S13.

Structured transitivity assessment identified important imbalances across populations, mechanisms of balance impairment, intervention platforms, comparator intensity, control subtypes, outcome domains, and reporting of concurrent physical activity or co-interventions. Transitivity was therefore considered plausible only at a broad balance-oriented exercise-category level and clinically fragile.

Figure 2 shows the geometry of the primary and broader randomized-trial networks. Node area represents the number of participants contributing to each node, and edge width represents the number of direct randomized comparisons between nodes. These graphical features describe evidence structure and should not be interpreted as effect size, evidence certainty, or intervention ranking.

### 3.3 Aging-public-health applicability of the evidence base

The primary randomized-trial network was aging-relevant but not exclusively geriatric. To clarify public health applicability, the 12 primary-network studies were descriptively mapped into three evidence-applicability categories: direct older-adult community evidence, aging-related clinical rehabilitation evidence, and indirect non-geriatric evidence.

Direct older-adult community evidence comprised four studies and 187 participants. These studies enrolled community-dwelling older adults, older women, or healthy older adults and therefore provided the most direct evidence for older-adult community balance-oriented intervention planning. Aging-related clinical rehabilitation evidence comprised six studies and 216 participants. These studies enrolled participants with neurological or functional-vulnerability conditions relevant to older-adult rehabilitation and fall-risk management, including multiple sclerosis, chronic stroke, and Parkinson disease. Indirect non-geriatric evidence comprised two studies and 88 participants, including athletes with nonspecific low back pain and healthy sedentary women. These studies contributed to network connectivity and comparative estimation but were treated as indirect evidence for older-adult public health interpretation.

In the broader randomized-trial sensitivity network, one additional study in community-dwelling older women contributed fall-related proxy evidence, whereas one additional healthy-adult pilot study contributed broader postural-control evidence. These studies were retained only in the broader randomized-trial sensitivity layer and did not replace the primary balance- and postural-control inference.

This mapping indicates that the available evidence can inform older-adult community health planning and aging-related rehabilitation interpretation, but it should not be treated as a purely geriatric trial synthesis. The distinction between direct older-adult community evidence, aging-related clinical rehabilitation evidence, and indirect non-geriatric evidence was therefore used to frame the public health and implementation interpretation in the Discussion.

### 3.4 Technology modality, delivery characteristics, and explicitly reported algorithmic features

The technology-assisted exercise node represented a heterogeneous group of digitally supported exercise formats, including virtual-reality exercise, augmented-reality exercise, Kinect or motion-sensing exercise, exergaming, active video-game exercise, interactive digital exercise, and home-based or remotely supported exercise formats. The technology-delivered Pilates node represented Pilates-based exercise delivered through telerehabilitation, synchronous video, or other digital communication platforms.

These interventions were interpreted as digitally supported or technology-assisted exercise formats, not as uniformly AI-driven interventions. No consistent explicit AI-driven decision support, machine-learning prediction, automated risk stratification, algorithm-guided progression, or automated adaptive personalization was reported across the included randomized studies. Sensor-derived measures and remote-monitoring functions, where present, were treated as digital monitoring or implementation features unless algorithmic decision-making or adaptive personalization was explicitly described.

Table 2 summarizes technology modality, delivery characteristics, explicitly reported AI or algorithmic features, and implementation gaps by intervention node. Technology-assisted exercise was the broadest digital category, but it should not be interpreted as evidence for a specific platform, device, AR/VR modality, or AI-enabled rehabilitation model. Technology-delivered Pilates represented a smaller hybrid category combining Pilates-based motor-control training with digital or remote supervision, but the evidence base remained sparse.

**Table 2:** Node-level interpretation of technology modality, delivery characteristics, and algorithmic-feature reporting in the final network.

Network node	Content represented in the node	Technology or delivery feature	Explicit AI or algorithmic feature reported	Interpretation boundary for this NMA
Control	Waitlist, no intervention, usual activity or usual care without structured exercise, education-only or minimal-contact control, no formal balance training or physical therapy, or other non-exercise reference conditions	No structured exercise, Pilates-specific training, or digital intervention	Not applicable	The control node was not clinically uniform and should not be interpreted as a pure placebo, no-contact condition, or strictly inactive comparator. Control subtype was considered in transitivity, sensitivity, and certainty interpretation.
Conventional exercise	Non-digital structured exercise, physical therapy, balance training, core-stability exercise, yoga/Otago-type exercise, or conventional rehabilitation without Pilates-specific or technology-mediated delivery	Active non-digital exercise or rehabilitation	Not applicable	This node combined heterogeneous active exercise and rehabilitation approaches. Differences in exercise content, dose, supervision, setting, and comparator intensity were treated as potential effect modifiers rather than as evidence of a single standardized intervention.
Pilates	Pilates-based exercise delivered without digital, remote, or technology-mediated delivery	Face-to-face or otherwise non-digital Pilates-specific motor-control training	No explicit AI or algorithmic feature reported	This node represents Pilates-specific exercise and should not be generalized to generic core training unless the source study explicitly defined the intervention as Pilates-based. Evidence for this node remained sparse and was interpreted cautiously.
Technology-assisted exercise	Non-Pilates exercise supported or augmented by digital technology, including virtual reality, augmented reality, Kinect or motion-sensing exercise, exergaming, active video-game exercise, interactive digital exercise, and related digitally supported balance or postural-control training	Digital feedback, cueing, motion tracking, interactive task practice, engagement support, or technology-mediated exercise delivery	No consistent explicit AI-driven decision support, machine-learning prediction, automated risk stratification, algorithm-guided progression, or adaptive personalization was reported	This was a broad digitally supported exercise node. The estimate should not be interpreted as platform-specific efficacy for virtual reality, augmented reality, Kinect, exergaming, any single device, or AI-enabled rehabilitation.
Technology-delivered Pilates	Pilates-based exercise delivered through telerehabilitation, synchronous video, digital communication, remote supervision, or other technology-mediated platforms	Remote or digitally mediated delivery of Pilates-specific exercise	No explicit AI or algorithmic feature reported	This sparse hybrid node retained Pilates-specific exercise content but differed from face-to-face Pilates in delivery mode, supervision, access requirements, and implementation context. It should be interpreted as preliminary evidence only, not as proof that digital Pilates delivery is superior to face-to-face Pilates.

AI, artificial intelligence; NMA, network meta-analysis. This table summarizes node-level technology and delivery characteristics for interpretation. Technology mapping was descriptive and was not used to define treatment rankings, establish platform-specific efficacy, or infer AI-enabled rehabilitation effects. Standard virtual reality, augmented reality, motion sensing, exergaming, remote video delivery, or telerehabilitation was not classified as AI-driven unless the source report explicitly described automated decision support, machine-learning prediction, automated risk stratification, algorithm-guided progression, or adaptive personalization. Detailed study-arm-level node coding and technology-feature mapping are provided in Supplementary Tables S8, S9.

### 3.5 Primary randomized-trial network meta-analysis

Primary randomized-trial network estimates versus control are shown in Table 3 and Figure 3. In the random-effects primary randomized-trial NMA, active intervention nodes generally showed favourable point estimates compared with control, but the strength and certainty of evidence varied across nodes.

**Table 3:** Primary randomized-trial network meta-analysis estimates versus control.

Intervention node versus control	Node-level evidence in the primary randomized-trial network	Participants in node	Hedges’ g versus control	95% CI	Certainty of evidence	Primary interpretation
Conventional exercise	7 analytic arms	139	0.33	−0.04 to 0.69	Very low	Favourable point estimate, but the confidence interval included the null; evidence was limited by sparse direct evidence, indirectness, and imprecision
Pilates	3 analytic arms	56	0.64	0.18 to 1.09	Very low	Favourable estimate versus control, but evidence was sparse and certainty was limited by indirectness, imprecision, and potential local direct–indirect disagreement
Technology-assisted exercise	8 analytic arms	163	0.69	0.38 to 1.01	Low	Favourable estimate versus control, but the node combined heterogeneous digitally supported exercise formats and should not be interpreted as platform-specific or AI-enabled efficacy
Technology-delivered Pilates	2 analytic arms	34	0.69	0.14 to 1.25	Very low	Favourable estimate versus control, but evidence was sparse and limited by delivery-mode indirectness and imprecision

CI, confidence interval; NMA, network meta-analysis. Estimates were obtained from the random-effects primary randomized-trial network meta-analysis. The primary randomized-trial network included 12 source-verified randomized studies, 26 randomized source arms represented as 25 analytic arms after same-node arm combination, and 491 participants. Hedges’ g values were calculated from harmonized post-intervention arm-level data using exact small-sample correction. Positive values indicate better balance or postural-control performance compared with control after outcome-direction harmonization. Node-level evidence refers to the number of analytic arms and participants contributing to each intervention node in the primary randomized-trial network, not the number of direct head-to-head comparisons with control. The control node included 5 analytic arms and 99 participants and was retained as the reference node. *p* values are not shown because interpretation was based on effect estimates, confidence intervals, evidence structure, and certainty of evidence rather than binary statistical significance. Certainty ratings are reported in detail in Supplementary Table S20. These estimates do not establish a definitive treatment hierarchy.

Conventional exercise showed a favourable point estimate versus control, but the confidence interval included the null value (Hedges’ g 0.33, 95% CI −0.04 to 0.69). Pilates showed a favourable estimate versus control (0.64, 0.18 to 1.09). Technology-assisted exercise also showed a favourable estimate versus control (0.69, 0.38 to 1.01). Technology-delivered Pilates showed a favourable estimate versus control (0.69, 0.14 to 1.25), but this node was supported by sparse evidence and should be interpreted cautiously.

Node-level evidence differed across active interventions. Conventional exercise was represented by 7 analytic arms and 139 participants, Pilates by 3 arms and 56 participants, technology-assisted exercise by 8 arms and 163 participants, and technology-delivered Pilates by 2 arms and 34 participants. The control node included 5 analytic arms and 99 participants. Confidence intervals overlapped across active interventions; therefore, these estimates were not interpreted as a definitive treatment hierarchy.

Among active-intervention comparisons, technology-assisted exercise showed a favourable network estimate compared with conventional exercise (Hedges’ g 0.37, 95% CI 0.11 to 0.62). However, this comparison combined heterogeneous populations, technology platforms, comparator intensity, and outcome domains, and certainty remained limited. Other major active-intervention comparisons were imprecise, sparse, or indirect-dependent and did not establish superiority among active interventions. Because outcomes were standardized across different balance and postural-control instruments, Hedges’ g values should be interpreted as relative standardized effects rather than direct clinical-unit changes.

### 3.6 Sensitivity and analytical-fragility analyses

Sensitivity and analytical-fragility analyses are summarized in Table 4 and Figure 4, with full estimates and evidence-structure diagnostics provided in Supplementary Tables S17, S18. The broader randomized-trial sensitivity network produced estimates of 0.37 (95% CI 0.02 to 0.72) for conventional exercise, 0.79 (0.49 to 1.09) for Pilates, 0.72 (0.42 to 1.03) for technology-assisted exercise, and 0.78 (0.27 to 1.30) for technology-delivered Pilates versus control. These results represented a sensitivity evidence layer and did not replace the primary inference.

**Table 4:** Sensitivity and analytical-fragility analyses of estimates versus control.

Analysis	Evidence base	Conventional exercise vs control	Pilates or Pilates-related node vs control	Technology-assisted exercise vs control	Technology-delivered Pilates vs control	Main interpretation
Primary randomized-trial NMA	12 studies; 25 analytic arms; 491 participants	0.33 [−0.04, 0.69]	0.64 [0.18, 1.09]	0.69 [0.38, 1.01]	0.69 [0.14, 1.25]	Main inferential analysis. Active nodes generally showed favourable point estimates, but conventional exercise included the null and certainty was low or very low
Broader randomized-trial sensitivity network	14 studies; 29 analytic arms; 606 participants	0.37 [0.02, 0.72]	0.79 [0.49, 1.09]	0.72 [0.42, 1.03]	0.78 [0.27, 1.30]	Direction remained favourable after adding broader randomized evidence, but this sensitivity layer did not replace the primary inference
Excluding potentially consequential baseline imbalance	11 studies; 23 analytic arms; 461 participants	0.35 [−0.02, 0.72]	0.65 [0.19, 1.10]	0.69 [0.37, 1.00]	0.70 [0.14, 1.25]	Estimates were similar to the primary analysis, but this was not a baseline-adjusted NMA
Non-exercise/minimal-intervention control sensitivity	11 studies; 23 analytic arms; 461 participants	0.45 [0.06, 0.83]	1.01 [0.43, 1.60]	0.77 [0.44, 1.09]	0.92 [0.32, 1.51]	Estimates increased after removing active non-exercise control evidence; interpreted as control-node structural sensitivity, not proof of robustness
Excluding active non-exercise Pilates-control study	11 studies; 23 analytic arms; 461 participants	0.45 [0.06, 0.83]	1.01 [0.43, 1.60]	0.77 [0.44, 1.09]	0.92 [0.32, 1.51]	Same numerical result as the non-exercise/minimal-intervention control sensitivity; removal changed the direct Pilates-control evidence structure
Excluding athlete/low-back-pain study	11 studies; 23 analytic arms; 441 participants	0.37 [−0.01, 0.75]	0.66 [0.20, 1.11]	0.68 [0.36, 1.00]	0.71 [0.15, 1.26]	Point-estimate direction was not dominated by the athletic low-back-pain study, but residual clinical heterogeneity remained
Clinical/older-rehabilitation population restriction	10 studies; 21 analytic arms; 403 participants	0.34 [−0.05, 0.73]	0.56 [0.05, 1.08]	0.66 [0.34, 0.98]	0.91 [0.15, 1.67]	Estimates remained generally favourable, but the restricted network remained sparse and clinically heterogeneous
Clinical/field-based balance-performance outcome restriction	9 studies; 18 analytic arms; 389 participants	0.21 [−0.28, 0.70]	0.48 [−0.10, 1.05]	0.63 [0.22, 1.04]	0.38 [−0.53, 1.29]	Estimates attenuated for conventional exercise, Pilates, and technology-delivered Pilates; this indicated outcome-domain sensitivity
Pilates-related node merging	11 studies; 23 analytic arms; 453 participants	0.33 [−0.03, 0.69]	0.65 [0.23, 1.08]	0.70 [0.38, 1.01]	Merged with Pilates-related node	Similar estimates after merging Pilates and technology-delivered Pilates did not establish equivalence between delivery formats
AR/VR-only feasibility analysis	4 studies; 9 analytic arms; 161 participants	0.39 [−0.29, 1.08]	Not estimable	0.86 [0.17, 1.54]	Not estimable	Feasibility check only. The restricted network did not support the full five-node comparative framework
Common-effect model	12 studies; 25 analytic arms; 491 participants	0.33 [−0.04, 0.69]	0.64 [0.18, 1.09]	0.69 [0.38, 1.01]	0.69 [0.14, 1.25]	Estimates were similar to the random-effects model in this sparse network; this was not interpreted as proof of clinical homogeneity
Direct–indirect evidence diagnostic	Primary randomized-trial network	Not the focus of the detected signal	Pilates vs. control: direct 0.07 [−0.65, 0.78]; indirect 1.01 [0.43, 1.60]; difference −0.95 [−1.87, −0.02]; P = 0.045	No statistically clear disagreement detected	No statistically clear disagreement detected	Potential local direct–indirect disagreement was identified for Pilates versus control and informed certainty downgrading

Values are Hedges’ g [95% CI] for each intervention node versus control unless otherwise specified. Positive values favour the intervention after outcome-direction harmonization. NMA, network meta-analysis. Sensitivity analyses were interpreted according to effect estimates, confidence intervals, network geometry, direct-evidence structure, and certainty implications rather than binary statistical significance. The broader randomized-trial network was a sensitivity evidence layer and did not replace the primary randomized-trial inference. The non-exercise/minimal-intervention control sensitivity and the exclusion of the active non-exercise Pilates-control study produced the same numerical result because the same study was removed. Common-effect and random-effects estimates being similar was not interpreted as evidence of true clinical homogeneity. Full sensitivity estimates, direct–indirect diagnostics, and contribution-matrix summaries are provided in Supplementary Tables S17, S18.

Exclusion of the study with potentially consequential selected-outcome baseline imbalance produced only small changes in active-versus-control estimates: 0.35 (95% CI −0.02 to 0.72) for conventional exercise, 0.65 (0.19 to 1.10) for Pilates, 0.69 (0.37 to 1.00) for technology-assisted exercise, and 0.70 (0.14 to 1.25) for technology-delivered Pilates. This sensitivity analysis did not constitute a baseline-adjusted NMA.

The non-exercise/minimal-intervention control sensitivity, which removed the active non-exercise Pilates-control study, yielded larger estimates: 0.45 (95% CI 0.06 to 0.83) for conventional exercise, 1.01 (0.43 to 1.60) for Pilates, 0.77 (0.44 to 1.09) for technology-assisted exercise, and 0.92 (0.32 to 1.51) for technology-delivered Pilates. This analysis changed the direct-evidence structure and was interpreted as control-node structural sensitivity rather than proof of robustness.

Exclusion of the athlete/low-back-pain study did not materially change point-estimate direction. Estimates were 0.37 (95% CI −0.01 to 0.75) for conventional exercise, 0.66 (0.20 to 1.11) for Pilates, 0.68 (0.36 to 1.00) for technology-assisted exercise, and 0.71 (0.15 to 1.26) for technology-delivered Pilates. Restriction to clinical or older-rehabilitation populations also retained generally favourable point estimates but did not eliminate clinical heterogeneity.

Outcome-domain restriction to clinical or field-based balance-performance outcomes attenuated several estimates. Conventional exercise was 0.21 (95% CI −0.28 to 0.70), Pilates 0.48 (−0.10 to 1.05), technology-assisted exercise 0.63 (0.22 to 1.04), and technology-delivered Pilates 0.38 (−0.53 to 1.29). This finding indicated sensitivity to outcome-domain selection and informed certainty assessment. The AR/VR-only feasibility analysis retained only a reduced three-node structure and did not support the full five-node comparative framework. Common-effect estimates were similar to random-effects estimates in the sparse primary network, but this was not interpreted as evidence of clinical homogeneity.

### 3.7 Exploratory P-score summaries, heterogeneity, and direct–indirect evidence

P-score summaries are reported in Supplementary Table S16 and Supplementary Figure S2. These values were retained only as exploratory supplementary summaries and were displayed in fixed conceptual order. They were not interpreted as evidence of statistically significant superiority, certainty of evidence, risk-of-bias status, clinical recommendation, or a definitive treatment hierarchy.

Network diagnostics are summarized in Supplementary Table S18 and Supplementary Figure S3. In the primary randomized-trial network, τ^2^ was <0.001, I^2^ was 0.0%, and Q was 8.65 (df = 9; *p* = 0.470). The design-by-treatment inconsistency test yielded *p* = 0.281. In the broader randomized-trial network, τ^2^ was <0.001, I^2^ was 0.0%, and Q was 9.51 (df = 11; *p* = 0.575), with design-by-treatment *p* = 0.779. Because the networks were small and sparse, the absence of statistically clear global heterogeneity or inconsistency signals was not interpreted as proof of clinical homogeneity or consistency.

Direct evidence was available for several comparisons, but multiple active-intervention comparisons remained supported by limited direct evidence or by indirect evidence only. Node-splitting identified a potential local direct–indirect disagreement for Pilates versus control in the primary network. The network estimate was 0.64 (95% CI 0.18 to 1.09), the direct estimate was 0.07 (−0.65 to 0.78), the indirect estimate was 1.01 (0.43 to 1.60), and the direct–indirect difference was −0.95 (−1.87 to −0.02; *p* = 0.045). This signal was not statistically clear in the broader randomized-trial network (*p* = 0.388). It was interpreted as a potential local incoherence concern rather than definitive proof of inconsistency. The comparison-adjusted funnel plot is shown in Supplementary Figure S4 and was considered exploratory only because the network contained few direct effects and sparse comparisons.

### 3.8 Risk of bias and certainty of evidence

Risk-of-bias judgments are summarized in Figure 1A and Supplementary Table S19, with study-level RoB2 judgments displayed in Supplementary Figure S5. Figure 1A includes all 14 source-verified randomized trials contributing to either randomized-trial evidence layer: 12 trials in the primary randomized-trial network and 2 trials included only in the broader randomized-trial sensitivity network. All 14 trials were judged as having some concerns overall under RoB2, and no trial was judged as high risk overall. The controlled study identified as using nonrandom first-come, first-served allocation was retained outside both randomized-trial networks and did not contribute to the RoB2 summaries or randomized-trial certainty assessments.

Comparison-level certainty ratings for the principal primary-network comparisons are summarized in Figure 1B and Supplementary Table S20. These ratings were based on the 12-study primary randomized-trial network; the two broader-only randomized trials did not contribute to the primary-network certainty ratings. Certainty was low for technology-assisted exercise versus control and very low for conventional exercise, Pilates, and technology-delivered Pilates versus control. Domain-specific downgrading reflected comparison-specific concerns relating to within-study bias, indirectness, imprecision, heterogeneity, and incoherence; data-availability concerns were additionally considered only when they could be attributed directly to the comparison being assessed.

Technology-assisted exercise versus control was rated as low certainty despite a favourable estimate because the intervention node combined heterogeneous digital platforms, populations, control subtypes, and outcome domains. Conventional exercise versus control was rated as very low certainty because the confidence interval included the null and direct evidence versus control was sparse. Pilates versus control was rated as very low certainty because direct evidence was sparse and a potential local direct–indirect disagreement was identified in the primary network. Technology-delivered Pilates versus control was rated as very low certainty because the node was supported by sparse evidence, the confidence interval was wide, and applicability was limited by delivery-mode differences. Overall, the primary analysis indicated a generally favourable direction of active-intervention point estimates versus control but did not establish a definitive treatment hierarchy, platform-specific superiority, automated decision-support efficacy, or adaptive-personalization effects.

## 4 Discussion

### 4.1 Principal findings

This systematic review and exploratory network meta-analysis compared Pilates, technology-assisted exercise, technology-delivered Pilates, conventional exercise, and control conditions for balance- and postural-control-related outcomes. Fall-related proxy evidence and pilot or broader randomized evidence were retained only in a broader randomized-trial sensitivity layer. After source verification, the main inferential evidence was restricted to source-verified randomized trials. The principal finding was a generally favourable direction of active-intervention point estimates versus control, not evidence that any intervention format was definitively superior.

In the primary randomized-trial network, conventional exercise showed a favourable point estimate versus control, but the confidence interval included the null. Pilates, technology-assisted exercise, and technology-delivered Pilates showed favourable estimates versus control, but all required cautious interpretation because of sparse evidence, clinical and intervention heterogeneity, indirectness, imprecision, or local evidence-structure concerns. The broader randomized-trial sensitivity network showed a similar overall direction but did not replace the primary randomized-trial inference.

The findings should not be interpreted as establishing a definitive hierarchy among active interventions. Certainty was low for technology-assisted exercise versus control and very low for conventional exercise, Pilates, and technology-delivered Pilates versus control. Confidence intervals overlapped across active interventions, several comparisons were sparse or indirect-dependent, and Pilates versus control showed a potential local direct–indirect disagreement in the primary network. Therefore, the most defensible interpretation is that the available randomized-trial evidence suggests a generally favourable direction for structured active exercise-based approaches versus control, while remaining insufficient for treatment ranking, platform-specific recommendations, or claims about specific digital decision-support efficacy or automated-personalization effects. Overall, the results are best understood as exploratory and hypothesis-generating comparative evidence rather than as a basis for selecting a single optimal intervention.

### 4.2 Interpretation within balance and postural-control rehabilitation

The generally favourable direction observed across active intervention nodes is consistent with the broader rehabilitation principle that structured exercise may improve components of postural stability through neuromuscular, sensory, mechanical, and motor-control pathways. Balance and postural control depend on lower-limb strength and mechanical function, trunk stability, sensory integration, anticipatory postural adjustment, dynamic coordination, and task-specific movement control (1, 5). Conventional exercise programmes may influence these domains through strengthening, gait training, flexibility training, balance tasks, and repeated functional practice (1, 2). However, in the primary randomized-trial network, the confidence interval for conventional exercise versus control included the null. Conventional exercise should therefore be interpreted as a clinically plausible, accessible, and adaptable comparator rather than as a definitively effective or superior node.

Pilates may provide a motor-control-oriented training stimulus, although this network meta-analysis was not designed to test mechanisms directly. Its emphasis on controlled trunk-centred movement, core stabilization, postural alignment, breathing coordination, movement precision, and body awareness provides a plausible rationale for improving postural-control performance (7, 10, 35). Rather than acting only through general physical conditioning, Pilates may influence movement organization, trunk-limb coordination, postural alignment, and anticipatory control. Nevertheless, the Pilates evidence base remained sparse, and Pilates versus control showed a potential local direct–indirect disagreement. These findings support Pilates as a plausible balance-oriented exercise approach, but they do not establish Pilates superiority or high-certainty efficacy.

Technology-assisted exercise may support balance and postural-control training through feedback-enriched and task-specific practice (13–15). Virtual-reality exercise, augmented-reality exercise, exergaming, Kinect-based or motion-sensing training, interactive gaming, telerehabilitation, and other digitally supported formats can provide visual or auditory feedback, external cues, interactive movement tasks, and enriched sensorimotor engagement (36, 37). These characteristics may support movement repetition, motivation, adherence, and real-time correction of postural errors. However, the present synthesis could not isolate which specific technology feature, platform, feedback mode, supervision model, immersion level, progression rule, or delivery setting contributed to the observed estimates. The favourable estimate for technology-assisted exercise should therefore be interpreted as an average estimate across a broad and heterogeneous digitally supported exercise category.

Recent meta-analytic evidence in community-dwelling adults aged 60 years or older suggests that digital physical-activity interventions may produce small improvements in muscle mechanical function, with larger effects reported for leg-strength-related outcomes in interactive formats, although the certainty of evidence was limited (38). This evidence provides a plausible, but not demonstrated, pathway through which digitally supported exercise could contribute to postural performance. The present network meta-analysis did not test this mechanism directly and cannot determine whether the observed estimates were driven by muscle-strength adaptation, task-specific balance practice, sensory feedback, motivation, supervision, or other intervention features.

### 4.3 Interpreting technology-assisted exercise and technology-delivered Pilates

Technology-assisted exercise and technology-delivered Pilates require particularly careful interpretation because both are clinically appealing but methodologically heterogeneous. Technology-assisted exercise was treated as an umbrella node to maintain network connectivity and reflect the available randomized evidence. This approach allowed synthesis across related digitally supported exercise interventions, but it also combined platforms and delivery modes that may not have identical mechanisms, training intensity, supervision requirements, accessibility barriers, or implementation requirements (39). The registered title emphasized AR/VR-based technology-assisted exercise, whereas the final technology-assisted node was broader and included non-immersive and digitally supported exercise formats. This protocol-relevant scope broadening preserved network connectivity but increased intervention indirectness and prevents AR/VR-specific or platform-specific conclusions. The technology-assisted node should therefore be interpreted as a broad digitally supported exercise category rather than as evidence for any single device, platform, or delivery model (13, 14).

Technology-delivered Pilates is conceptually important but currently under-established. A hybrid format that combines Pilates-based motor-control training with digital delivery or technology-mediated supervision may be useful for remote rehabilitation, home-based exercise support, and individuals requiring structured but adaptable training. However, the current evidence is too sparse to determine whether technology-delivered Pilates provides additional benefit beyond Pilates alone, technology-assisted exercise alone, or conventional exercise. The favourable point estimate for technology-delivered Pilates versus control should therefore be interpreted as preliminary and hypothesis-generating rather than as evidence that remote or digital Pilates delivery is superior to face-to-face Pilates.

The descriptive technology-modality audit further shows that technology-assisted exercise combined VR-based, AR-based, Kinect or motion-sensing, exergaming, active video-game, telerehabilitation, and other digitally supported exercise formats, whereas technology-delivered Pilates mainly represented telerehabilitation or synchronous digital Pilates delivery. These interventions were technology-supported, but the included trials did not consistently report automated decision support, machine-learning prediction, automated risk stratification, algorithm-guided progression, or adaptive personalization (18). Therefore, the present findings should not be interpreted as evidence for a specific digital decision-support model or automated-personalization effect. The exploratory P-score summaries reinforce this caution. In sparse networks, treatment rankings may appear precise even when the underlying comparative evidence is limited, indirect, or uncertain (40, 41). P-scores should therefore be viewed only as exploratory supplementary summaries and not as evidence of statistically significant superiority, certainty, risk-of-bias status, clinical recommendation, or a reliable treatment hierarchy (21, 22).

### 4.4 Transitivity, sensitivity, incoherence, and certainty of evidence

The sensitivity analyses should be interpreted as assessments of analytical fragility and evidence structure rather than as confirmation of robustness. The broader randomized-trial sensitivity network retained a generally favourable direction after adding broader randomized evidence, but it incorporated fall-related proxy or pilot/broader evidence and therefore served only as supporting sensitivity evidence; it did not replace the primary randomized-trial inference. Exclusion of the study with potentially consequential baseline imbalance produced similar estimates, but this was not a baseline-adjusted network meta-analysis. The non-exercise or minimal-intervention control sensitivity produced larger estimates after removal of active non-exercise control evidence, indicating that control-node composition materially affected the evidence structure. This should be interpreted as control-node structural sensitivity, not as confirmation of robustness.

The outcome-domain sensitivity analysis was particularly important. When the analysis was restricted to clinical or field-based balance-performance outcomes, estimates attenuated for conventional exercise, Pilates, and technology-delivered Pilates, whereas technology-assisted exercise remained favourable. However, this did not remove uncertainty because the technology-assisted node remained clinically and technologically heterogeneous. This suggests that the conclusions were partly sensitive to whether instrumented or laboratory-based postural-control outcomes were pooled with clinical or field-based balance-performance outcomes. Population-restricted analyses also did not eliminate clinical heterogeneity because the network continued to include heterogeneous neurological, older-adult, healthy or sedentary, and rehabilitation-related contexts. Together, these findings support cautious interpretation and justify downgrading for indirectness and imprecision. To avoid double counting, population and pathology differences, intervention-node breadth, technology-platform heterogeneity, control-node variability, outcome-domain sensitivity, and clinically fragile transitivity were considered collectively under indirectness rather than as separate independent downgrades. Applicability limitations arising from these factors were therefore captured within the indirectness assessment.

No statistically clear global heterogeneity or design-level inconsistency signal was detected in either the primary or broader randomized-trial network. However, the absence of a statistically detectable signal should not be interpreted as proof of clinical homogeneity or consistency because the networks were small and several pairwise comparisons were supported by limited direct evidence or indirect evidence only. Statistical inconsistency tests and comparison-adjusted funnel plots have limited interpretability in sparse evidence networks (19, 20, 33). Local direct–indirect evidence was more informative for specific comparisons. In the primary network, Pilates versus control showed a potential local direct–indirect disagreement. This signal was not statistically clear in the broader network but was treated as an incoherence concern in the certainty assessment. The potential direct–indirect disagreement was considered specifically under the incoherence domain and was not counted again under indirectness or heterogeneity.

Across all 14 source-verified randomized trials contributing to either randomized-trial evidence layer, the overall RoB2 judgment was some concerns. The comparison-level certainty ratings discussed here, however, were based on the 12-study primary randomized-trial network; the two broader-only randomized trials did not contribute to the primary-network certainty ratings. Certainty was low for technology-assisted exercise versus control and very low for conventional exercise, Pilates, and technology-delivered Pilates versus control.

The number of certainty levels downgraded within each domain is reported explicitly in Supplementary Table S20. Sparse direct evidence, small sample sizes, confidence-interval width, and overlapping confidence intervals were considered under imprecision. Clinical heterogeneity was not additionally downgraded under statistical heterogeneity when the same concern had already been captured under indirectness. Review-wide data-availability concerns resulted in an additional comparison-specific downgrade only when they could be linked directly to the comparison being assessed. The prespecified range of −0.30 to +0.30 Hedges’ g was used solely as an analytical reference for imprecision assessment and was not treated as a validated minimal clinically important difference, a clinical-equivalence margin, or a threshold for clinical importance.

These low and very low certainty ratings indicate that the observed comparative estimates may change as additional evidence becomes available. Accordingly, the findings should be interpreted as exploratory and hypothesis-generating and should not be used to support definitive superiority claims, treatment rankings, platform-specific recommendations, or claims of automated decision-support or adaptive-personalization efficacy (21, 42).

### 4.5 Aging public health, community intervention design, and targeted management implications

The findings have cautious but important implications for aging public health, community-based balance promotion, and targeted health management. The overall pattern suggests that structured active exercise-based interventions may be reasonable candidates for balance-oriented care compared with inactive, usual-activity, education-only, waitlist, or other non-exercise control conditions. However, the current evidence does not demonstrate fall-incidence reduction, long-term functional preservation, or durability of intervention effects. Fall-related proxy evidence was retained only in the broader sensitivity layer and did not replace the primary balance- or postural-control inference.

For older-adult public health practice, the results should therefore be interpreted as supporting a risk-stratified exercise-planning framework rather than a ranking-driven choice of a single best intervention. In community settings, older adults should first be screened for fall history, balance limitation, gait impairment, frailty, multimorbidity, medication-related fall risk, cognitive or sensory impairment, fear of falling, home-environment hazards, and access barriers. This initial screening can help distinguish older adults who may safely participate in community-based exercise from those requiring clinician-supervised rehabilitation or referral.

Intervention selection should be matched to functional status, clinical vulnerability, supervision needs, and local resources. Community-dwelling older adults with mild balance limitation and low-to-moderate fall risk may be appropriate candidates for supervised group-based conventional exercise, Pilates, or structured balance-oriented exercise. Pilates may be considered when the rehabilitation goal emphasizes trunk control, postural alignment, breathing coordination, movement precision, body awareness, and low-impact progressive training. Conventional exercise remains clinically relevant because it is accessible, adaptable, and feasible in community, outpatient, and rehabilitation settings. Technology-assisted exercise may be relevant when feedback, interactive engagement, task-specific practice, home-based delivery, remote access, or technology-mediated supervision is desired. Technology-delivered Pilates may represent a promising hybrid option for remote or home-based support, but the present evidence is too limited to determine whether it provides additional benefit beyond Pilates alone, technology-assisted exercise alone, or conventional exercise.

For older adults with neurological disease, recurrent falls, marked gait instability, severe frailty, or substantial multimorbidity, digitally supported or home-based exercise should not be treated as an unsupervised substitute for professional care. In these higher-risk groups, technology-assisted exercise may be better positioned as an adjunct to clinician-supervised rehabilitation, with individualized progression, fall-risk monitoring, adverse-event surveillance, and clear referral criteria. This distinction is important because the evidence base was aging-relevant but not exclusively geriatric, and the applicability mapping distinguished direct older-adult community evidence from aging-related clinical rehabilitation evidence and indirect non-geriatric evidence.

From an implementation perspective, community programmes should not focus only on short-term balance or postural-control gains. They should also monitor adherence, adverse events, falls, near falls, functional decline, care use, discontinuation reasons, usability, and participant confidence. For digitally supported exercise, implementation should account for device access, internet connectivity, digital literacy, language accessibility, privacy, data security, usability, technical support, caregiver involvement, therapist availability, and cost. These factors may determine whether digital exercise reduces access barriers or widens disparities among older adults (1, 16, 17).

Table 5 translates these findings into an aging-public-health framework. Because certainty was low or very low and the evidence base was clinically heterogeneous, this table should not be interpreted as a treatment-ranking, platform-procurement recommendation, or policy endorsement of a single exercise format. Instead, it summarizes how different balance-oriented exercise options may be considered within risk-stratified community health management for older adults.

**Table 5:** Aging-public-health translation framework for balance-oriented exercise interventions.

Aging-related public health need	Target older-adult subgroup	Candidate intervention option	Community or care setting	Safety and equity requirements	Evidence boundary
Mild balance decline or early postural-control limitation	Community-dwelling older adults with low-to-moderate fall risk	Conventional exercise, Pilates, or structured balance-oriented group exercise	Senior centres, community health stations, primary-care-linked exercise programmes	Instructor supervision, progressive dosing, screening for pain, dizziness, unstable disease, and fall history	Evidence supports a generally favourable direction of active exercise versus control, but does not establish a definitive hierarchy
Reduced access to facility-based rehabilitation	Older adults with transport barriers, limited rehabilitation access, or preference for home-based exercise	Technology-assisted exercise or technology-delivered Pilates as supervised or hybrid options	Home-based, remote, hybrid community-care, or telerehabilitation-supported delivery	Device access, internet connectivity, digital literacy, usability support, privacy protection, caregiver or therapist support	Current evidence does not support platform-specific superiority, device-procurement decisions, or AI-enabled rehabilitation claims
Neurological or complex functional vulnerability	Older adults or aging-related rehabilitation patients with Parkinson disease, stroke, multiple sclerosis, recurrent falls, or marked gait instability	Clinician-supervised conventional rehabilitation, balance training, or digitally supported exercise as an adjunct	Outpatient rehabilitation, specialist referral pathways, community rehabilitation programmes	Professional assessment, fall-risk monitoring, adverse-event surveillance, individualized progression, referral criteria	Evidence is clinically heterogeneous; unsupervised digital delivery should not be generalized to high-risk patients
Fear of falling, low confidence, or reduced activity participation	Older adults with avoidance behaviour or low motivation but without severe instability	Pilates, interactive technology-assisted exercise, or group-based balance exercise	Community group exercise, supervised home exercise, hybrid digital programmes	Motivation support, adherence tracking, social support, safe exercise environment	Fall-related proxy evidence remains secondary and should not be interpreted as direct fall-incidence reduction
Public health programme planning	Community-level older-adult populations requiring scalable fall-risk and functional-maintenance strategies	Stepped balance-oriented exercise pathway combining screening, risk stratification, referral, and follow-up	Primary care, public health services, community health centres, municipal aging services	Workforce training, cost planning, equity safeguards, data privacy, monitoring of falls, adherence, and adverse events	The review informs programme design and evidence gaps, but not definitive policy adoption of a single intervention or platform

Overall, the present network meta-analysis can support clinical and public health reasoning by clarifying the comparative evidence structure, applicability boundaries, and implementation gaps. It should not be used as a stand-alone basis for choosing a single superior intervention, procuring a specific technology platform, or recommending unsupervised digital delivery for high-risk older adults. Stronger policy and implementation conclusions will require trials that evaluate fall incidence, functional decline, durability, safety, adherence, cost-effectiveness, usability, technology acceptance, caregiver or therapist involvement, and digital equity in clearly defined older-adult subgroups.

### 4.6 Strengths and methodological contributions

This review has several strengths. First, the protocol was registered in PROSPERO before quantitative synthesis, and the review transparently documented the protocol-relevant scope broadening from AR/VR-based technology-assisted exercise to a broader digitally supported technology-assisted exercise node. Rather than presenting this broadening as fully protocol-concordant, the final review treated it as an acknowledged methodological limitation and examined its implications through technology-node mapping, AR/VR-only feasibility analysis, transitivity assessment, and conservative certainty downgrading. Second, source verification strengthened the validity of the evidence structure by restricting the primary inference to source-verified randomized trials and retaining the nonrandomized controlled study outside the randomized-trial networks.

Third, the review applied explicit intervention-node definitions, outcome-domain rules, endpoint-prioritization procedures, same-node arm-combination rules, and outcome-direction harmonization before effect-size construction. This was important because the included studies used heterogeneous balance, postural-control, and fall-related measures. Fourth, the use of Hedges’ g with exact small-sample correction, structured baseline and transitivity audits, control-node and concurrent-activity audits, outcome-domain sensitivity analysis, direct–indirect diagnostics, and random-effects contribution summaries provided a more transparent account of analytical uncertainty than a single primary network estimate alone. Finally, the review emphasized reproducibility by linking the search record, full-text decision audit, locked extraction dataset, effect-size construction, network estimation, sensitivity analyses, figures, certainty judgments, and software-environment information through a reproducible analytical workflow (23, 43).

A methodological contribution of this review is that it treated the evidence base as exploratory rather than forcing a definitive treatment hierarchy from a sparse network. This approach is particularly important in emerging rehabilitation fields where intervention categories are evolving, technology-assisted formats vary substantially, and direct head-to-head trials remain limited.

### 4.7 Limitations and future research

Several limitations should be acknowledged. First, the evidence network was small and sparse. The primary randomized-trial network included 12 studies, 25 analytic arms, and 491 participants, and the broader randomized-trial sensitivity network included 14 studies, 29 analytic arms, and 606 participants. Some intervention nodes and direct comparisons were supported by few studies and small sample sizes, limiting the precision of estimates, the stability of exploratory P-score summaries, and the ability to detect inconsistency or small-study effects. To address this limitation, the present review added an aging-public-health applicability mapping layer that distinguished direct older-adult community evidence from aging-related clinical rehabilitation evidence and indirect non-geriatric evidence.

Second, not all contributing trials enrolled older adults. Several studies included younger or non-geriatric populations, including athletes with low back pain, healthy adults, and healthy sedentary women. Consequently, applicability to older-adult care is indirect for part of the network, and the observed comparative estimates should not be generalized to older populations without age-specific confirmation. This population mismatch contributed to clinical indirectness and was considered in the transitivity assessment and certainty judgments.

Third, clinical and methodological heterogeneity was substantial. Included studies differed in population, pathology, mechanism of balance impairment, baseline capacity, intervention content, dose, supervision level, setting, comparator conditions, outcome instruments, and measurement timing. Although standardized Hedges’ g values allowed synthesis across different instruments, they cannot fully eliminate differences in clinical meaning across balance scales, postural-control tests, and fall-related proxies (30, 31). Transitivity was therefore considered clinically fragile, and the comparative estimates should be interpreted as broad standardized summaries rather than as directly interchangeable clinical effects.

Fourth, technology-assisted exercise was necessarily treated as an umbrella node (13, 14). This approach supported network connectivity and reflected the available evidence, but it combined heterogeneous platforms, including AR/VR-based exercise, exergaming, Kinect-based or motion-sensing training, interactive gaming, telerehabilitation, and other digitally supported formats. This broadening from the registered AR/VR-focused title to a broader technology-assisted node was a protocol-relevant scope deviation and limits platform-specific interpretation. The technology-assisted exercise node should therefore be interpreted as an evidence-connecting category for exploratory synthesis rather than as a clinically uniform intervention or a platform-specific estimate. Future studies should examine whether specific technology formats, feedback types, immersion levels, supervision models, progression rules, or delivery settings produce different effects on balance and postural-control outcomes.

Fifth, control conditions were heterogeneous. Control arms included active non-exercise comparator content, waitlist or no intervention, usual activity, usual care, education-only or minimal-contact control, and absence of formal balance training or physical therapy. Because control subtype affected sensitivity estimates, comparisons versus control should not be interpreted as comparisons against a single standardized inactive condition. Reporting of habitual physical activity, outside exercise, co-interventions, and contamination was also incomplete; not reported should not be interpreted as absent. These issues may have influenced observed differences between active intervention nodes and control.

Sixth, the synthesis was limited to available reports with compatible quantitative data. Reports without extractable or derivable post-intervention arm-level data for the prioritized eligible outcome were not entered into Hedges’ g construction. Study authors were not contacted for missing numerical data, and no unpublished arm-level data were incorporated into the reported analyses. Unavailable numerical data may therefore have introduced evidence-availability or reporting-bias concerns. The findings should be interpreted as a synthesis of available and extractable evidence rather than as a complete reconstruction of all conducted trials.

Seventh, the analysis focused on post-intervention outcomes and did not establish durability of effects after follow-up. Baseline-adjusted effects could not be consistently synthesized because adjusted estimates and change-score standard deviations were not consistently available. Although a baseline audit and exclusion sensitivity analysis were performed, the primary analysis remained based on post-intervention Hedges’ g and was not a baseline-adjusted NMA. Adherence, adverse events, intervention fidelity, technology usability, implementation cost, privacy, technology acceptance, and equity were also not synthesized as network outcomes. These factors are especially important for digitally supported and home- or community-delivered exercise interventions, because short-term balance or postural-control improvement alone is insufficient to determine scalability, safety, sustainability, or public health value in older-adult care pathways (16, 17, 31).

Eighth, certainty of evidence limited inference. Technology-assisted exercise versus control was rated as low certainty, and conventional exercise, Pilates, and technology-delivered Pilates versus control were rated as very low certainty. Sparse direct evidence, overlapping confidence intervals, indirectness, imprecision, within-study concerns, control-node heterogeneity, outcome-domain sensitivity, and potential local incoherence limited confidence in the comparative estimates. Accordingly, active-intervention comparisons and P-score summaries should be interpreted only as exploratory.

Ninth, the review has important limitations regarding technology-specific decision-support inference. The quantitatively synthesized interventions were mainly digitally supported or technology-assisted exercise programmes rather than explicitly automated decision-support interventions. The present findings should therefore not be interpreted as direct evidence for automated intervention selection, machine-learning-guided progression, or adaptive personalization. Future trials are needed to evaluate whether sensor-informed monitoring, automated progression rules, therapist-in-the-loop decision support, remote monitoring dashboards, cost, privacy, technology acceptance, and equity of access can be safely and effectively integrated into older-adult care settings.

Tenth, the available evidence was not sufficient for reliable subgroup, dose–response, or meta-regression analyses. Differences in age, clinical condition, intervention dose, supervision intensity, technology platform, control condition, outcome domain, digital literacy, delivery setting, and baseline fall-risk profile may all influence treatment response, but the current network was too small to evaluate these effect modifiers formally. Future evidence syntheses should revisit these questions when more adequately powered head-to-head trials become available.

Future studies should prioritize adequately powered, preregistered, head-to-head randomized trials comparing Pilates, technology-assisted exercise, technology-delivered Pilates, and conventional exercise under standardized conditions. Trials should report intervention dose, progression, supervision, adherence, adverse events, technology-platform characteristics, comparator content, intervention fidelity, follow-up duration, and implementation context using transparent intervention-reporting principles (26–28, 31). For digitally supported and emerging technology-assisted exercise, future trials should additionally report usability, acceptability, technology access requirements, digital literacy demands, therapist involvement, caregiver support, home-based feasibility, data privacy considerations, implementation cost, and equity-relevant factors. A more consistent outcome framework for balance, postural control, fall-related proxies, and functional decline would strengthen future evidence synthesis by reducing outcome heterogeneity and improving comparability across trials. In particular, technology-delivered Pilates should be evaluated in larger comparative trials to determine whether combining Pilates-based motor-control training with digital or technology-mediated delivery provides additional benefit beyond Pilates alone, technology-assisted exercise alone, or conventional exercise.

### 4.8 Overall interpretation of the evidence

Taken together, this review supports a cautious, evidence-graded interpretation. The available evidence indicates a generally favourable direction of active exercise-based interventions versus control, but the network remains sparse, clinically heterogeneous, and largely low or very low certainty. The most defensible conclusion is not that one intervention is universally superior, but that different exercise formats may offer distinct practical, mechanistic, and implementation-related advantages under different clinical, community, home-based, and digital-care contexts.

Technology-assisted exercise should be interpreted as a broad digitally supported exercise category rather than as evidence for a specific platform, device, or automated decision-support model. Technology-delivered Pilates remains a conceptually promising but insufficiently established hybrid approach. Therefore, the present findings should be viewed as exploratory comparative evidence for refining balance-oriented rehabilitation and technology-supported exercise research, rather than as proof that any single platform, exercise format, or digital delivery model is optimal. Stronger conclusions about relative effectiveness, durability, safety, implementation, digital equity, and automated personalization will require larger, directly comparative, and implementation-oriented randomized trials.

## 5 Conclusion

This systematic review and exploratory network meta-analysis found that point estimates for active exercise-based interventions generally favoured the active intervention over control for balance- and postural-control-related outcomes. However, inference was limited by a small and clinically heterogeneous randomized-trial network, sparse direct comparisons, control-node variability, outcome-domain sensitivity, clinically fragile transitivity, and low or very low certainty of evidence. Technology-assisted exercise showed a favourable estimate versus control with low certainty, whereas conventional exercise, Pilates, and technology-delivered Pilates were supported by very low-certainty evidence. The broader randomized-trial sensitivity network showed a similar overall direction but did not replace the primary randomized-trial inference.

The current evidence does not establish a reliable intervention hierarchy, a single superior strategy, platform-specific superiority, fall-incidence reduction, durable functional preservation, or efficacy of automated decision support or adaptive personalization. Technology-assisted exercise should be interpreted as a broad digitally supported exercise category rather than evidence for a specific AR/VR platform, device, or automated decision-support model. Technology-delivered Pilates should be viewed as a promising but insufficiently established hybrid approach.

For aging public health, the main contribution of this review is to clarify the comparative evidence structure, applicability boundaries, and implementation gaps for balance-oriented exercise in older-adult and aging-related rehabilitation contexts. The findings may inform risk-stratified community health-management frameworks, including screening, supervised exercise delivery, home-based or remote support, referral pathways, adherence monitoring, adverse-event surveillance, and digital-equity planning. They should not be used as a stand-alone basis for selecting a single preferred intervention, procuring a specific technology platform, or recommending unsupervised digital delivery for high-risk older adults. Future trials should evaluate fall incidence, functional decline, durability, safety, adherence, cost-effectiveness, usability, technology acceptance, caregiver or therapist involvement, and digital equity in clearly defined older-adult subgroups.

## Data Availability

The original contributions presented in the study are included in the article/Supplementary material, further inquiries can be directed to the corresponding authors.
