# Supplementary material for: Pilates and AR/VR-based and other technology-assisted exercise for balance and postural control in older-adult and aging-related rehabilitation contexts: a systematic review and exploratory network meta-analysis
Source: Front Public Health. 2026 Aug 7;14:1899309. doi: 10.3389/fpubh.2026.1899309 (PMC13495933; doi:10.3389/fpubh.2026.1899309)
Supplement: Supplementary file 1 [file Table_1.DOCX]

**Supplementary Figure S1. Exploratory league table of primary randomized-trial NMA estimates.** Values are Hedges’ g with 95% confidence intervals for the row intervention versus the column intervention in the primary randomized-trial network. Positive Hedges’ g values favour the row intervention after outcome-direction harmonization. Asterisks indicate comparisons for which the 95% confidence interval crosses the null value. League-table estimates were interpreted as exploratory comparative summaries and do not establish intervention superiority, certainty of evidence, or a definitive treatment hierarchy.

**Supplementary Figure S2. Exploratory P-score summaries in the primary and broader randomized-trial networks.** Panel A shows P-score summaries from the primary randomized-trial network. Panel B shows P-score summaries from the broader randomized-trial sensitivity network. Nodes are displayed in a fixed conceptual order to avoid implying a treatment ranking. P-scores are exploratory summaries within each fitted network only and should not be interpreted as evidence of statistically significant superiority, certainty of evidence, clinical recommendation, risk-of-bias status, or a definitive intervention hierarchy. Differences between the primary and broader networks should be interpreted alongside sparse-node, transitivity, and outcome-domain caveats.

**Supplementary Figure S3. Global diagnostics, direct-evidence availability, and local direct–indirect evidence.** Panel A summarizes global heterogeneity and design-level inconsistency diagnostics for the primary randomized-trial network and the broader randomized-trial sensitivity network. Panel B shows direct-evidence availability across possible pairwise comparisons in the primary and broader networks. Panel C summarizes the Pilates-versus-control direct–indirect evidence signal in the primary network. Absence of a statistically clear global heterogeneity or design-level inconsistency signal was not interpreted as evidence of clinical homogeneity or consistency because the networks were small and sparse. The Pilates-versus-control direct–indirect signal was interpreted as a potential local incoherence concern rather than definitive proof of inconsistency.

**Supplementary Figure S4. Exploratory comparison-adjusted funnel plot for the primary randomized-trial network.** The comparison-adjusted funnel plot was used only as a descriptive visual check because the primary randomized-trial network contained few direct contrasts and several sparse comparisons. Direct contrast groups are displayed to show the distribution of comparison-adjusted Hedges’ g values and standard errors. This plot was not interpreted as a formal test of publication bias, reporting bias, or small-study effects.

**Supplementary Figure S5. RoB2 traffic-light plot for all 14 source-verified randomized trials contributing to either randomized-trial evidence layer.** Twelve trials contributed to the primary randomized-trial network, and two contributed only to the broader randomized-trial sensitivity network. Each row represents one source-verified randomized trial, and each column represents a RoB2 domain or the overall judgment. Cell symbols indicate domain-level judgments: “+” denotes low risk, and “?” denotes some concerns. No high-risk RoB2 judgments were observed. The controlled study retained outside the randomized-trial networks because of nonrandom allocation was not included in this RoB2 traffic-light plot and was not used to determine randomized-trial certainty judgments.
