# Supplementary material for: Pilates and AR/VR-based and other technology-assisted exercise for balance and postural control in older-adult and aging-related rehabilitation contexts: a systematic review and exploratory network meta-analysis
Source: Front Public Health. 2026 Aug 7;14:1899309. doi: 10.3389/fpubh.2026.1899309 (PMC13495933; doi:10.3389/fpubh.2026.1899309)
Supplement: Supplementary file 2 [file Table_2.DOCX]

**Supplementary Table S1. Search strategy, information sources, retrieval records, and evidence-allocation linkage**

**Supplementary Table S1A. Information sources and search dates**

| **Source** | **Platform/interface** | **Search date** | **Fields searched** | **Limits applied** | **Records identified** |
| --- | --- | --- | --- | --- | --- |
| PubMed/MEDLINE | PubMed, National Library of Medicine | 2026-05-02 | Title/Abstract | No additional date or language limits at search stage | 1,703 |
| Web of Science | Web of Science Core Collection | 2026-05-02 | Topic | No additional date or language limits at search stage | 2,560 |
| Scopus | Scopus | 2026-05-02 | Title/Abstract/Keywords | No additional date or language limits at search stage | 2,687 |
| Embase | Embase | 2026-05-02 | Title/Abstract/Keywords; Emtree terms where applicable | No additional date or language limits at search stage | 3,460 |
| CINAHL | EBSCOhost/CINAHL Complete | 2026-05-02 | Title/Abstract/Keywords | No additional date or language limits at search stage | 722 |
| APA PsycInfo | EBSCOhost/APA PsycInfo | 2026-05-02 | Title/Abstract/Keywords | No additional date or language limits at search stage | 1,141 |
| Cochrane Library/CENTRAL | Cochrane Library | 2026-05-02 | Title/Abstract/Keywords | No additional date or language limits at search stage | 3,507 |
| ClinicalTrials.gov | ClinicalTrials.gov expert search | 2026-05-02 | Basic search and intervention search fields | No additional date or language limits at search stage | 1,415 |
| Total bibliographic database records | PubMed, Web of Science, Scopus, Embase, CINAHL, APA PsycInfo, and Cochrane Library | 2026-05-02 | Database-specific fields | — | 15,780 |
| Total register records | ClinicalTrials.gov | 2026-05-02 | Register-specific search fields | — | 1,415 |
| Total records identified/exported | Databases and register combined | 2026-05-02 | — | — | 17,195 |

**Supplementary Table S1B. Database-specific search strategies**

| **Source** | **Search strategy used/database-adapted syntax** | **Records retrieved** |
| --- | --- | --- |
| PubMed/MEDLINE | ((Pilates[Title/Abstract] OR "Pilates exercise"[Title/Abstract] OR "Pilates training"[Title/Abstract] OR "Pilates-based exercise"[Title/Abstract] OR "Pilates-based training"[Title/Abstract]) OR (("virtual reality"[Title/Abstract] OR "augmented reality"[Title/Abstract] OR exergam*[Title/Abstract] OR "active video game"[Title/Abstract] OR "active video games"[Title/Abstract] OR Kinect[Title/Abstract] OR Wii[Title/Abstract] OR Nintendo[Title/Abstract] OR "Ring Fit"[Title/Abstract] OR telerehabilitation[Title/Abstract] OR "tele-rehabilitation"[Title/Abstract] OR "technology-assisted"[Title/Abstract] OR "technology-based"[Title/Abstract]) AND (exercise[Title/Abstract] OR training[Title/Abstract] OR rehabilitation[Title/Abstract] OR intervention[Title/Abstract] OR "exercise therapy"[Title/Abstract] OR "physical activity"[Title/Abstract] OR "physical training"[Title/Abstract]))) AND (balance[Title/Abstract] OR "postural control"[Title/Abstract] OR "postural stability"[Title/Abstract] OR "postural balance"[Title/Abstract] OR posture[Title/Abstract] OR "center of pressure"[Title/Abstract] OR "centre of pressure"[Title/Abstract] OR stabil*[Title/Abstract] OR fall*[Title/Abstract] OR "fall risk"[Title/Abstract]) | 1,703 |
| Web of Science Core Collection | TS=((Pilates OR "Pilates exercise" OR "Pilates training" OR "Pilates-based exercise" OR "Pilates-based training") OR (("virtual reality" OR "augmented reality" OR exergam* OR "active video game" OR "active video games" OR Kinect OR Wii OR Nintendo OR "Ring Fit" OR telerehabilitation OR "tele-rehabilitation" OR "technology-assisted" OR "technology-based") AND (exercise OR training OR rehabilitation OR intervention OR "exercise therapy" OR "physical activity" OR "physical training"))) AND TS=(balance OR "postural control" OR "postural stability" OR "postural balance" OR posture OR "center of pressure" OR "centre of pressure" OR stabil* OR fall* OR "fall risk") | 2,560 |
| Scopus | TITLE-ABS-KEY((pilates OR "mat pilates" OR "reformer pilates" OR "pilates-based" OR "pilates exercise" OR "pilates training") OR ("virtual reality" OR "augmented reality" OR exergam* OR exergaming OR exergame OR kinect OR wii OR nintendo OR "ring fit" OR "active video game" OR "active video games" OR "interactive game" OR "interactive games" OR telerehabilitation OR "tele-rehabilitation" OR "technology-assisted exercise" OR "technology-based exercise" OR "digital exercise")) AND TITLE-ABS-KEY(balance OR "postural control" OR "postural stability" OR "postural balance" OR posture OR "center of pressure" OR "centre of pressure" OR stabil* OR fall* OR "fall risk") | 2,687 |
| Embase | ('pilates'/exp OR pilates:ti,ab,kw OR 'pilates exercise':ti,ab,kw OR 'pilates training':ti,ab,kw OR 'pilates-based exercise':ti,ab,kw OR 'pilates-based training':ti,ab,kw OR (('virtual reality'/exp OR 'augmented reality'/exp OR 'telerehabilitation'/exp OR 'exergaming'/exp OR 'active video game':ti,ab,kw OR 'active video games':ti,ab,kw OR 'virtual reality':ti,ab,kw OR 'augmented reality':ti,ab,kw OR exergam*:ti,ab,kw OR kinect:ti,ab,kw OR wii:ti,ab,kw OR nintendo:ti,ab,kw OR 'ring fit':ti,ab,kw OR telerehabilitation:ti,ab,kw OR 'tele-rehabilitation':ti,ab,kw OR 'technology-assisted':ti,ab,kw OR 'technology-based':ti,ab,kw) AND (exercise/exp OR 'exercise therapy'/exp OR rehabilitation/exp OR exercise:ti,ab,kw OR training:ti,ab,kw OR rehabilitation:ti,ab,kw OR intervention:ti,ab,kw OR 'exercise therapy':ti,ab,kw OR 'physical activity':ti,ab,kw OR 'physical training':ti,ab,kw))) AND ('postural balance'/exp OR balance:ti,ab,kw OR 'postural control':ti,ab,kw OR 'postural stability':ti,ab,kw OR 'postural balance':ti,ab,kw OR posture:ti,ab,kw OR 'center of pressure':ti,ab,kw OR 'centre of pressure':ti,ab,kw OR stabil*:ti,ab,kw OR fall*:ti,ab,kw OR 'fall risk':ti,ab,kw) AND ('randomized controlled trial'/exp OR random*:ti,ab,kw OR trial:ti,ab,kw OR rct:ti,ab,kw OR 'controlled trial':ti,ab,kw) | 3,460 |
| CINAHL | (TI (pilates OR "mat pilates" OR "reformer pilates" OR "pilates-based" OR "pilates exercise" OR "pilates training" OR "virtual reality" OR "augmented reality" OR exergam* OR exergaming OR exergame OR kinect OR wii OR nintendo OR "ring fit" OR "active video game" OR "active video games" OR "interactive game" OR "interactive games" OR telerehabilitation OR "tele-rehabilitation" OR "technology-assisted exercise" OR "digital exercise") OR AB (pilates OR "mat pilates" OR "reformer pilates" OR "pilates-based" OR "pilates exercise" OR "pilates training" OR "virtual reality" OR "augmented reality" OR exergam* OR exergaming OR exergame OR kinect OR wii OR nintendo OR "ring fit" OR "active video game" OR "active video games" OR "interactive game" OR "interactive games" OR telerehabilitation OR "tele-rehabilitation" OR "technology-assisted exercise" OR "digital exercise")) AND (balance OR "postural control" OR "postural stability" OR "postural balance" OR posture OR "center of pressure" OR "centre of pressure" OR stabil* OR fall* OR "fall risk") | 722 |
| APA PsycInfo | (pilates OR "mat pilates" OR "reformer pilates" OR "pilates-based" OR "pilates exercise" OR "pilates training" OR "virtual reality" OR "augmented reality" OR exergam* OR exergaming OR exergame OR kinect OR wii OR nintendo OR "ring fit" OR "active video game" OR "active video games" OR "interactive game" OR "interactive games" OR telerehabilitation OR "tele-rehabilitation" OR "technology-assisted exercise" OR "digital exercise") AND (balance OR "postural control" OR "postural stability" OR "postural balance" OR posture OR "center of pressure" OR "centre of pressure" OR stabil* OR fall* OR "fall risk") | 1,141 |
| Cochrane Library/CENTRAL | (Pilates OR "Pilates exercise" OR "Pilates training" OR "Pilates-based exercise" OR "Pilates-based training" OR (("virtual reality" OR "augmented reality" OR exergam* OR "active video game*" OR Kinect OR Wii OR Nintendo OR "Ring Fit" OR telerehabilitation OR "tele-rehabilitation" OR "technology-assisted" OR "technology-based") AND (exercise OR training OR rehabilitation OR intervention OR "exercise therapy" OR "physical activity" OR "physical training"))) AND (balance OR "postural control" OR "postural stability" OR "postural balance" OR posture OR "center of pressure" OR "centre of pressure" OR stabil* OR fall* OR "fall risk*") in Title Abstract Keyword | 3,507 |
| ClinicalTrials.gov | AREA[BasicSearch](balance OR "postural control" OR "postural stability" OR "postural balance" OR "fall risk" OR falls) AND AREA[InterventionSearch](Pilates OR "virtual reality" OR "augmented reality" OR exergame OR exergaming OR Kinect OR Wii OR Nintendo OR "Ring Fit" OR telerehabilitation OR "tele-rehabilitation" OR "technology-assisted") | 1,415 |

**Supplementary Table S1C. Search record, deduplication, retrieval, and revised PRISMA/NMA linkage**

| **Search or allocation item** | **Number** | **Source/derivation** | **Link to PRISMA or evidence-allocation stage** | **Verification status** |
| --- | --- | --- | --- | --- |
| Records identified from bibliographic databases | 15,780 | PubMed, Web of Science, Scopus, Embase, CINAHL, APA PsycInfo, and Cochrane Library exports | Identification | Author-verified against exported search files and source-level search records |
| Records identified from trial register | 1,415 | ClinicalTrials.gov export | Identification | Author-verified against register export and search record |
| Total records identified/exported | 17,195 | Bibliographic database records plus ClinicalTrials.gov records | Identification | Verified by source-level record totals |
| Duplicate records removed before screening | 8,137 | DOI-priority and title-based deduplication | Records removed before screening | Author-verified against deduplication audit |
| Records screened by title and abstract | 9,058 | Total records identified minus duplicate records | Screening | Verified by PRISMA arithmetic |
| Records excluded by title and abstract | 8,992 | Records screened minus reports sought for retrieval | Screening | Verified by PRISMA arithmetic |
| Reports sought for retrieval | 66 | Full-text retrieval set | Retrieval | Author-verified against full-text audit set |
| Reports not retrieved | 0 | All 66 full-text reports were obtained | Retrieval | Author-verified |
| Full-text reports assessed for eligibility/allocation | 66 | Final full-text assessment set | Eligibility/allocation | Verified in Supplementary Tables S3 and S4 |
| Reports not entered into quantitative synthesis after full-text assessment | 38 | Reports without extractable or derivable compatible arm-level post-intervention data for the prioritized eligible outcome, or without compatible quantitative input for the revised synthesis | Eligibility/allocation | Verified in Supplementary Table S4; author-contact status reported in Supplementary Table S4B |
| Final evidence base after full-text assessment | 28 evidence items | Fourteen randomized studies contributing to at least one randomized-trial NMA layer, one nonrandomized controlled study retained only as contextual/nonrandomized controlled evidence, and 13 qualitative/contextual reports | Included evidence base | Verified in Supplementary Tables S3–S6 |
| Studies included in the primary randomized-trial NMA | 12 studies | Source-verified randomized studies with eligible balance or postural-control outcomes and compatible arm-level post-intervention data | Main inferential synthesis | Verified in Supplementary Tables S5–S7 and S13–S14 |
| Randomized source arms represented in the primary randomized-trial NMA | 26 randomized source arms | One study contributed two same-node conventional-exercise arms that were combined before contrast construction | Main inferential synthesis | Verified in Supplementary Tables S7–S8 |
| Analytic arms included in the primary randomized-trial NMA | 25 analytic arms | Source arms after same-node arm combination | Main inferential synthesis | Verified in Supplementary Tables S7–S8 and S13–S14 |
| Participants included in the primary randomized-trial NMA | 491 participants | Sum of participants contributing to analytic arms in the source-verified primary randomized-trial network | Main inferential synthesis | Verified in Supplementary Tables S7 and S14 |
| Additional randomized studies included only in the broader randomized-trial NMA | 2 studies | Randomized broader-layer evidence, including fall-related proxy or broader-layer evidence defined in the final analytical amendment | Sensitivity evidence layer | Verified in Supplementary Tables S5–S7 and S15 |
| Studies included in the broader randomized-trial NMA in total | 14 studies | Twelve primary randomized studies plus two broader-only randomized studies | Sensitivity evidence layer | Verified in Supplementary Tables S5–S7 and S15 |
| Randomized source arms represented in the broader randomized-trial NMA | 30 randomized source arms | Source arms before same-node arm combination | Sensitivity evidence layer | Verified in Supplementary Tables S7–S8 and S15 |
| Analytic arms included in the broader randomized-trial NMA | 29 analytic arms | Source arms after same-node arm combination | Sensitivity evidence layer | Verified in Supplementary Tables S7–S8 and S15 |
| Participants included in the broader randomized-trial NMA | 606 participants | Sum of participants contributing to analytic arms in the broader randomized-trial network | Sensitivity evidence layer | Verified in Supplementary Tables S7 and S15 |
| Nonrandomized controlled evidence retained outside the randomized-trial primary network | 1 study | Source verification identified nonrandom first-come, first-served allocation | Contextual/nonrandomized controlled evidence | Verified in Supplementary Tables S5 and S19 |
| Reports retained for qualitative/contextual synthesis only | 13 reports | Relevant reports not entered into effect-size estimation | Contextual evidence | Verified in Supplementary Table S4 |
| Unresolved or pending reports after final full-text assessment | 0 | Final full-text closure complete | Eligibility/allocation | Verified |

**Supplementary Table S1D. Revised final PRISMA and synthesis-closure summary**

| **PRISMA or synthesis domain** | **Final revised number** | **Interpretation** |
| --- | --- | --- |
| Records identified from bibliographic databases | 15,780 | Search records exported from seven bibliographic databases |
| Records identified from trial register | 1,415 | ClinicalTrials.gov records |
| Total records identified/exported | 17,195 | Total search yield before deduplication |
| Duplicate records removed | 8,137 | Records removed before title/abstract screening |
| Records screened | 9,058 | Unique records screened by title and abstract |
| Records excluded | 8,992 | Records excluded at title/abstract stage |
| Reports sought for retrieval | 66 | Full-text reports selected for retrieval |
| Reports not retrieved | 0 | No full-text report was unavailable |
| Reports assessed for eligibility/allocation | 66 | Full-text reports assessed and assigned a final decision |
| Reports not entered into quantitative synthesis after full-text assessment | 38 | Reports not entered into NMA effect-size estimation because compatible arm-level quantitative input was unavailable or could not be derived for the prioritized eligible outcome |
| Final evidence base after full-text assessment | 28 evidence items | Fourteen randomized quantitative studies contributing to at least one randomized-trial NMA layer, one nonrandomized controlled study retained outside the randomized-trial primary network, and 13 qualitative/contextual reports |
| Studies included in the primary randomized-trial NMA | 12 | Main inferential network based on source-verified randomized trials |
| Randomized source arms represented in the primary randomized-trial NMA | 26 | Source arms before same-node arm combination |
| Analytic arms included in the primary randomized-trial NMA | 25 | Analytic arms after same-node arm combination |
| Participants included in the primary randomized-trial NMA | 491 | Participants contributing to the main inferential network |
| Studies included in the broader randomized-trial NMA | 14 | Sensitivity evidence layer including the primary randomized studies plus two broader-only randomized studies |
| Randomized source arms represented in the broader randomized-trial NMA | 30 | Source arms before same-node arm combination |
| Analytic arms included in the broader randomized-trial NMA | 29 | Analytic arms after same-node arm combination |
| Participants included in the broader randomized-trial NMA | 606 | Participants contributing to the broader randomized-trial network |
| Nonrandomized controlled evidence retained outside the randomized-trial primary network | 1 study | Not used to determine the primary randomized-trial NMA conclusions |
| Reports retained for qualitative/contextual synthesis only | 13 | Relevant reports used for contextual evidence only |
| Final unresolved reports | 0 | Full closure achieved |

Note. NMA, network meta-analysis; PRISMA, Preferred Reporting Items for Systematic Reviews and Meta-Analyses. Search records were identified from seven bibliographic databases and one trial register on 2 May 2026. Duplicate removal was performed before title/abstract screening using DOI-priority matching followed by title-based checking. The 66 full-text reports were all retrieved and assigned final eligibility or allocation decisions. After final source verification of allocation procedures, the primary inferential synthesis was restricted to source-verified randomized trials. The primary randomized-trial network included 12 studies, 26 randomized source arms represented as 25 analytic arms after same-node arm combination, and 491 participants. The broader randomized-trial network included 14 randomized studies, 30 randomized source arms represented as 29 analytic arms, and 606 participants. One controlled study using nonrandom allocation was retained outside the randomized-trial primary network and was not used to determine the primary randomized-trial conclusions. Detailed full-text decisions, missing-data retrieval decisions, study-design verification, arm-level extraction, and network estimates are reported in Supplementary Tables S3–S15.

**Supplementary Table S2. PICOS eligibility criteria, revised evidence-layer allocation rules, intervention-node definitions, and outcome hierarchy**

**Supplementary Table S2A. PICOS eligibility criteria**

| **PICOS domain** | **Inclusion criteria** | **Exclusion criteria** | Application in the final review |
| --- | --- | --- | --- |
| Population | Adults, older adults, or clinical/athletic populations in which balance-, postural-control-, or fall-related outcomes were assessed | Studies without relevant balance-, postural-control-, or fall-related outcomes; studies in which the eligible outcome could not be linked to the review question | Included populations could differ clinically, but potential effect modifiers such as age, pathology, baseline balance/fall-risk profile, mechanism of balance impairment, setting, supervision, and dose were extracted and incorporated into the structured transitivity assessment |
| Intervention | Pilates, technology-assisted exercise, technology-delivered Pilates, conventional exercise, or comparable structured exercise/rehabilitation interventions | Non-exercise interventions, pharmacological interventions, surgery, passive therapy alone, or education-only programmes without an active exercise component | Eligible intervention arms were coded into the prespecified network taxonomy: control, conventional exercise, Pilates, technology-assisted exercise, and technology-delivered Pilates. The technology-assisted node was interpreted as a broad digitally augmented exercise category rather than a platform-specific intervention |
| Comparator | Control, usual activity/usual care, waitlist/no intervention, education-only/minimal-contact control, conventional exercise, Pilates, technology-assisted exercise, technology-delivered Pilates, or another eligible active exercise comparator | Single-arm designs without comparative data; comparator conditions not linkable to the network taxonomy; comparator conditions containing structured exercise but incorrectly labelled as “control” | Comparator content was source-verified. Conditions containing structured exercise, physical therapy, balance training, or active rehabilitation were classified as conventional exercise rather than control. Control conditions were subclassified for a control-node composition audit |
| Outcomes | Balance-, postural-control-, or fall-related outcomes with extractable post-intervention arm-level n, mean, and SD, or data sufficient to derive compatible values | Outcomes unrelated to balance, postural control, or fall-related performance; pain-only, strength-only, adherence-only, satisfaction-only, or quality-of-life-only outcomes when not linked to an eligible balance/postural-control endpoint; outcomes without compatible quantitative input | The prioritized eligible outcome was selected according to the prespecified hierarchy. Positive standardized effects were harmonized to represent better performance. Fall-related proxy outcomes were retained only in the broader randomized-trial network |
| Study design | Randomized or controlled comparative intervention studies were eligible for full-text review and evidence mapping | Reviews, protocols without extractable data, conference abstracts without compatible quantitative input, case reports, non-comparative designs, qualitative-only studies, and observational studies without an intervention comparison | The main inferential synthesis was restricted to source-verified randomized controlled trials. Controlled studies with nonrandom allocation were retained outside the randomized-trial primary network and were not used to determine the primary conclusions |
| Data format | Arm-level post-intervention n, mean, and SD, or equivalent numerical information from which compatible values could be derived | No extractable or derivable arm-level post-intervention quantitative data for the prioritized eligible outcome | Studies without compatible quantitative input were not entered into NMA effect-size estimation and were retained in the full-text decision log. Missing-data retrieval and author-contact status are documented separately |

**Supplementary Table S2B. Revised evidence-layer allocation rules**

| **Evidence layer/allocation** | **Required criteria** | **Quantitative synthesis status** | Role in the final review |
| --- | --- | --- | --- |
| Primary randomized-trial NMA | Source-verified randomized controlled trial; met core PICOS criteria; reported an eligible balance or postural-control outcome; provided extractable or derivable post-intervention arm-level n, mean, and SD; intervention arms compatible with the network taxonomy | Included in the primary randomized-trial network | Main inferential evidence layer. This layer was used for the principal comparative estimates and certainty judgments |
| Broader randomized-trial NMA | Source-verified randomized controlled trial; compatible with node coding; provided extractable or derivable quantitative data; met prespecified broader-layer criteria, such as randomized pilot evidence, fall-related proxy outcomes, or broader eligible balance/postural-control scope | Included only in the broader randomized-trial network, together with the primary randomized-trial studies | Sensitivity evidence layer used to examine directional sensitivity after adding broader randomized evidence. It did not replace the primary analysis or serve as confirmatory evidence. |
| Nonrandomized controlled evidence outside the randomized-trial networks | Controlled comparative intervention study with nonrandom allocation, such as first-come, first-served allocation, or another allocation procedure not compatible with randomized-trial inference | Not included in the primary or broader randomized-trial NMA | Retained outside the randomized-trial primary network as contextual/nonrandomized controlled evidence. It was not used to determine the primary conclusions or randomized-trial certainty judgments |
| Qualitative/contextual evidence only | Relevant to Pilates, technology-assisted exercise, digital exercise, telerehabilitation, balance training, postural control, or fall-related rehabilitation, but lacking compatible quantitative input or not meeting quantitative NMA criteria | Not entered into NMA effect-size estimation | Used to contextualize the evidence landscape, technology modalities, implementation gaps, and missing-data limitations |
| Excluded from quantitative synthesis after full-text assessment | Failed key PICOS requirements or lacked extractable/derivable post-intervention arm-level data for the prioritized eligible outcome | Not entered into NMA | Retained in the full-text audit trail. Missing-data retrieval or author-contact status was documented where applicable |
| Final unresolved/pending status | No report remained unclear after final full-text assessment | Not applicable | Audit confirmation that final full-text closure was achieved |

**Supplementary Table S2C. Intervention-node operational definitions**

| **Network node** | **Operational definition** | **Examples of included formats** | **Key interpretation boundary** |
| --- | --- | --- | --- |
| Control | No active exercise, waitlist/no intervention, usual activity/usual care without structured exercise, education-only/minimal-contact control, or other non-exercise comparator conditions | Waitlist, no formal exercise, no physical therapy, usual activity, education-only/no exercise, minimal-contact control | Control was not treated as clinically uniform. Control subtype was audited because variation in usual care, education, contact, and non-exercise co-interventions may affect transitivity and comparative estimates |
| Conventional exercise | Structured non-technology-assisted exercise, physical therapy, balance training, core-stability exercise, yoga/Otago-type exercise, or conventional rehabilitation without a Pilates-specific or digital-delivery component | Balance training, conventional physical therapy, yoga/Otago-type training, non-digital rehabilitation, non-technology exercise | This node was heterogeneous in content and dose. Comparator intensity and exercise content were treated as potential effect modifiers |
| Pilates | Pilates-based exercise delivered without digital, remote, or technology-mediated delivery | Face-to-face mat Pilates, supervised Pilates exercise, non-digital Pilates-based motor-control training | This node represents Pilates-specific motor-control exercise, not generic core training unless the study explicitly defined the intervention as Pilates-based |
| Technology-assisted exercise | Non-Pilates exercise materially augmented, supported, or delivered by digital technology, including feedback, cueing, interactive task practice, motion sensing, exergaming, or digitally supported balance/postural-control training | Virtual reality exercise, augmented reality exercise, exergaming, Kinect/motion-sensing exercise, interactive digital exercise, Ring Fit or other active video-game exercise | This node represents a broad digitally augmented exercise category. It should not be interpreted as evidence for a specific platform or as evidence that VR, AR, exergaming, and motion-sensing systems are interchangeable |
| Technology-delivered Pilates | Pilates-based training delivered through remote, digital, telecommunication, video, or technology-mediated platforms | Synchronous video Pilates, remote/tele-Pilates, digitally mediated Pilates supervision, online Pilates programmes | This node retains Pilates-specific motor-control content but differs from face-to-face Pilates in delivery mode, supervision, access requirements, and implementation context |

**Supplementary Table S2D. Outcome hierarchy and direction harmonization**

| **Outcome priority level** | **Eligible outcome type** | **Examples** | **Direction rule** | **Use in analysis** |
| --- | --- | --- | --- | --- |
| Priority 1 | Clinical or field-based balance-performance outcomes | BBS, Mini-BESTest, FABS, SEBT, FRT, Stork test | Higher values generally indicate better balance performance; lower-is-better measures were reversed before Hedges’ g calculation | Preferred outcome type for the primary randomized-trial NMA when available; also used for the clinical/field-based balance-performance sensitivity analysis |
| Priority 2 | Instrumented or laboratory-based postural-control outcomes | LOS, SOT equilibrium score, CoP displacement, CoP sway area, instrumented postural-sway outcomes | Direction harmonized before effect-size calculation; lower-is-better outcomes were reversed | Used when a clinical/field-based balance-performance outcome was unavailable or when postural control was the prioritized eligible construct |
| Priority 3 | Fall-related proxy outcomes | FES-I or comparable fear-of-falling/falls-efficacy proxy outcomes | Lower values indicate better fall-related status; direction reversed before effect-size calculation | Retained only in the broader randomized-trial network and not used to replace the primary balance/postural-control analysis |
| Not eligible for quantitative synthesis | Outcomes not compatible with standardized continuous-effect construction or not aligned with the review construct | Pain-only, strength-only, quality-of-life-only, adherence-only, satisfaction-only, or non-balance outcomes | Not applicable | Not entered into NMA effect-size estimation |

**Supplementary Table S2E. Endpoint selection and analysis interpretation rules**

| **Rule** | **Application** |
| --- | --- |
| Immediate post-intervention assessment | When multiple post-intervention time points were available, the immediate post-intervention assessment was used for the primary quantitative synthesis |
| Direction harmonization | All outcomes were harmonized so that positive Hedges’ g values represented better balance-, postural-control-, or fall-related performance |
| Post-intervention effect construction | Post-intervention arm-level n, mean, and SD were used because change-score SDs and adjusted estimates were not consistently available across studies |
| Same-node arm combination | When more than one randomized arm within a study represented the same network node, same-node arms were combined before contrast construction using standard arm-combination formulae |
| Baseline comparability | Baseline values for the selected outcome were extracted where available and summarized using standardized baseline differences. Baseline auditing was used for sensitivity assessment and certainty interpretation, not as a baseline-adjusted NMA |
| Transitivity interpretation | Transitivity was assessed structurally using study-level and comparison-level effect modifiers. It was considered plausible only at a broad balance-oriented exercise-category level and clinically fragile because important effect modifiers were unevenly distributed across comparisons |
| Treatment ranking | League tables and P-score summaries were retained only as exploratory supplementary outputs and were not interpreted as evidence of superiority or a definitive intervention hierarchy |
| Certainty interpretation | Certainty ratings were comparison-specific and used to contextualize effect estimates. They were not used to validate exploratory rankings or to establish a best intervention |

**Note.** BBS, Berg Balance Scale; CoP, center of pressure; FABS, Fullerton Advanced Balance Scale; FES-I, Falls Efficacy Scale-International; FRT, Functional Reach Test; LOS, limits of stability; Mini-BESTest, Mini Balance Evaluation Systems Test; NMA, network meta-analysis; PICOS, population, intervention, comparator, outcome, and study design; RCT, randomized controlled trial; SD, standard deviation; SEBT, Star Excursion Balance Test; SMD, standardized mean difference; SOT, Sensory Organization Test. The primary inferential synthesis was restricted to source-verified randomized trials. Nonrandomized controlled evidence was retained outside the randomized-trial primary network and did not determine the primary conclusions. Outcome direction was harmonized before effect-size construction so that positive Hedges’ g values indicated better balance-, postural-control-, or fall-related performance.

**Supplementary Table S3. PRISMA flow summary and revised evidence-allocation audit**

Supplementary Table S3A. Full-text-stage PRISMA allocation summary after final source verification

| **PRISMA/allocation stage** | **Number** | **Unit counted** | Role in the final review |
| --- | --- | --- | --- |
| Full-text reports assessed and assigned a final decision | 66 | Reports | Final full-text eligibility and allocation audit |
| Reports not entered into NMA effect-size estimation because compatible quantitative input was unavailable or could not be derived | 38 | Reports | Retained in the full-text decision log; missing-data retrieval and author-contact status reported separately |
| Randomized studies included in the primary randomized-trial NMA | 12 | Studies | Main inferential evidence layer based on source-verified randomized trials with eligible balance or postural-control outcomes |
| Randomized source arms represented in the primary randomized-trial NMA | 26 | Randomized source arms | Original randomized arms before same-node arm combination |
| Analytic arms included in the primary randomized-trial NMA | 25 | Analytic arms | Analytic arms after combining two same-node conventional-exercise arms within one randomized study |
| Participants included in the primary randomized-trial NMA | 491 | Participants | Participants contributing to the main inferential randomized-trial network |
| Additional randomized studies included only in the broader randomized-trial NMA | 2 | Studies | Randomized broader-layer evidence, including prespecified broader/pilot or fall-related proxy evidence |
| Randomized studies included in the broader randomized-trial NMA in total | 14 | Studies | Twelve primary randomized studies plus two broader-only randomized studies |
| Randomized source arms represented in the broader randomized-trial NMA | 30 | Randomized source arms | Source arms before same-node arm combination |
| Analytic arms included in the broader randomized-trial NMA | 29 | Analytic arms | Analytic arms after same-node arm combination |
| Participants included in the broader randomized-trial NMA | 606 | Participants | Participants contributing to the broader randomized-trial sensitivity network |
| Nonrandomized controlled evidence retained outside the randomized-trial primary network | 1 | Study | Controlled study with nonrandom allocation; retained as contextual/nonrandomized controlled evidence and not used to determine primary randomized-trial conclusions |
| Additional qualitative/contextual reports retained outside NMA effect-size estimation | 13 | Reports | Relevant contextual evidence used to describe the broader evidence landscape, technology modalities, and implementation or reporting gaps |
| Reports/studies remaining unresolved after final full-text assessment | 0 | Reports/studies | Final full-text closure achieved |

**Supplementary Table S3B. Revised final allocation of all 66 full-text reports**

| **Final allocation category** | **Number** | **Quantitative synthesis status** | **Role in manuscript** | **Detailed source** |
| --- | --- | --- | --- | --- |
| Included in primary randomized-trial NMA | 12 studies | Entered primary randomized-trial quantitative synthesis | Main inferential evidence base | Table 1; Supplementary Tables S5–S8 and S13–S14 |
| Included in broader randomized-trial NMA only | 2 studies | Entered broader randomized-trial quantitative synthesis only | Sensitivity evidence layer | Supplementary Tables S5–S8 and S15 |
| Nonrandomized controlled evidence retained outside randomized-trial networks | 1 study | Not entered into the primary or broader randomized-trial NMA | Contextual/nonrandomized controlled evidence; not used for primary conclusions | Supplementary Tables S5, S19, and S22 |
| Qualitative/contextual evidence only | 13 reports | Not entered into NMA effect-size estimation | Used to contextualize the review question, technology landscape, implementation gaps, and reporting limitations | Supplementary Table S4 |
| Excluded from NMA effect-size estimation because compatible quantitative input was unavailable or not derivable | 38 reports | Not entered into NMA | Retained in the full-text audit trail; author-contact/missing-data retrieval status documented where applicable | Supplementary Tables S4 and S4B |
| Final unresolved reports after full-text assessment | 0 reports | None | Final full-text closure complete | Supplementary Table S4 |
| Total full-text reports assigned a final decision | 66 reports | Completed | Final 66-report allocation closure | Supplementary Tables S3–S4 |

**Supplementary Table S3C. Revised randomized-trial network layers and source-arm accounting**

| **Network layer** | **Studies** | **Source arms** | **Analytic arms** | **Participants** | **Evidence role** | **Key allocation rule** |
| --- | --- | --- | --- | --- | --- | --- |
| Primary randomized-trial NMA | 12 | 26 | 25 | 491 | Main inferential layer | Restricted to source-verified randomized trials reporting eligible balance or postural-control outcomes with compatible arm-level post-intervention data |
| Broader randomized-trial NMA | 14 | 30 | 29 | 606 | Sensitivity layer | Included the 12 primary randomized studies plus 2 additional randomized broader-layer studies with prespecified broader/pilot or fall-related proxy evidence |
| Nonrandomized controlled evidence outside randomized-trial NMA | 1 | Not applicable to randomized-trial arm accounting | Not included in randomized-trial NMA | Not included in randomized-trial NMA totals | Contextual/nonrandomized controlled evidence | Controlled study with nonrandom allocation; excluded from randomized-trial primary inference |
| Qualitative/contextual evidence only | 13 reports | Not applicable | Not applicable | Not applicable | Contextual evidence | Relevant reports not entered into effect-size estimation |
| Reports excluded from NMA effect-size estimation because compatible quantitative input was unavailable or not derivable | 38 reports | Not applicable | Not applicable | Not applicable | Full-text audit trail | Reports without extractable or derivable arm-level numerical input for the prioritized eligible outcome |

Supplementary Table S3D. Final evidence-allocation decisions

| Final decision | **Implementation** | **Rationale** | **Effect on synthesis** |
| --- | --- | --- | --- |
| Restriction of the main inferential network to randomized trials | Source-verified randomized trials were retained in the primary and broader randomized-trial networks | To ensure compatibility with randomized-trial inference, RoB2 assessment, and comparison-level certainty judgments | Primary network revised to 12 studies, 25 analytic arms, and 491 participants |
| Removal of nonrandomized allocation from the randomized-trial primary network | One controlled study with nonrandom allocation was retained outside the randomized-trial networks | Nonrandom allocation was not compatible with the randomized-trial primary inference | Nonrandomized controlled evidence was not used to determine the primary conclusions |
| Same-node arm combination | Two same-node conventional-exercise arms within one randomized study were combined before contrast construction | To avoid treating two same-node arms from the same study as independent comparators and to preserve correct source-arm accounting | Primary randomized-trial network represented 26 randomized source arms as 25 analytic arms |
| Broader randomized-trial sensitivity layer retained | Two additional randomized broader-layer studies were retained only in the broader randomized-trial NMA | To examine directional sensitivity after adding prespecified broader/pilot or fall-related proxy evidence | Broader randomized-trial network included 14 studies, 29 analytic arms, and 606 participants |
| Missing quantitative input retained in audit trail | Reports without compatible numerical data were not imputed | To avoid introducing unavailable or unpublished numerical values into effect-size construction | Thirty-eight reports remained outside NMA effect-size estimation; missing-data retrieval documented separately |
| Contextual evidence retained | Relevant reports not entering NMA were retained for qualitative/contextual synthesis | To preserve transparency of the evidence landscape and avoid discarding clinically relevant but non-synthesizable evidence | Thirteen contextual reports plus one nonrandomized controlled study were retained outside the randomized-trial NMA layers |

Note. NMA, network meta-analysis; PRISMA, Preferred Reporting Items for Systematic Reviews and Meta-Analyses. The 66-report audit refers to all full-text reports assigned a final eligibility or allocation decision after full-text assessment. After final source verification of allocation procedures, the main inferential synthesis was restricted to randomized trials. The primary randomized-trial network included 12 studies, 26 randomized source arms represented as 25 analytic arms after same-node arm combination, and 491 participants. The broader randomized-trial network included 14 randomized studies, 30 randomized source arms represented as 29 analytic arms, and 606 participants. One controlled study with nonrandom allocation was retained outside the randomized-trial primary network and did not determine the primary conclusions. Detailed full-text decisions, missing-data retrieval, study-design verification, arm-level extraction, node coding, effect-size construction, and network estimates are provided in Supplementary Tables S4–S15.

**Supplementary Table S4. Full-text-stage allocation audit for all 66 reports after final evidence allocation**

Supplementary Table S4A. Final full-text allocation summary after final source verification

| **Final revised allocation category** | **Number** | **Unit counted** | **Final quantitative status** | Role in the final review |
| --- | --- | --- | --- | --- |
| Included in primary randomized-trial NMA | 12 | Studies/reports | Entered primary randomized-trial quantitative synthesis | Main inferential evidence base |
| Included in broader randomized-trial NMA only | 2 | Studies/reports | Entered broader randomized-trial quantitative synthesis only | Sensitivity evidence layer; did not replace the primary analysis |
| Nonrandomized controlled evidence outside randomized-trial NMA | 1 | Study/report | Not entered into primary or broader randomized-trial NMA | Contextual nonrandomized controlled evidence; not used to determine primary conclusions |
| Qualitative/contextual evidence only | 13 | Reports | Not entered into NMA effect-size estimation | Used to contextualize the evidence landscape, technology modalities, implementation gaps, and reporting limitations |
| Excluded from NMA effect-size estimation because compatible quantitative input was unavailable or not derivable | 38 | Reports | Not entered into NMA effect-size estimation | Retained in the full-text audit trail; missing-data derivation attempts and author-contact status documented in Supplementary Table S4B |
| Final unresolved reports | 0 | Reports | Not applicable | Final full-text closure achieved |
| Total full-text reports assessed and assigned a final decision | 66 | Reports | Completed | Complete full-text-stage eligibility and allocation audit |

**Supplementary Table S4B. Missing-data retrieval, derivation attempts, and author-contact status for reports not entering NMA effect-size estimation**

**Supplementary Table S4B-1. Missing-data handling framework**

| **Item** | Final handling in the final review |
| --- | --- |
| Full-text reports assessed | 66 reports |
| Reports not entered into NMA effect-size estimation because compatible quantitative input was unavailable or not derivable | 38 reports |
| Required quantitative input for NMA effect-size construction | Arm-level post-intervention n, mean, and SD for the prioritized eligible outcome, or numerical information sufficient to derive compatible values without unverifiable assumptions |
| Primary reason for non-entry into NMA effect-size estimation | Compatible arm-level post-intervention quantitative input for the prioritized eligible outcome was unavailable, incomplete, or not derivable |
| Derivation sources checked | Full text, tables, figures, supplementary materials, and reported statistical summaries where available |
| Derivation rule | Values were considered derivable only when compatible arm-level post-intervention means and SDs could be obtained or calculated without unverifiable assumptions |
| Imputation rule | Missing post-intervention means or SDs were not imputed using unverifiable assumptions |
| Author-contact status | Study authors were not contacted for missing numerical data for the analyses reported in this review |
| Additional unpublished numerical data incorporated | None |
| Consequence for synthesis | Reports without compatible quantitative input were retained in the full-text audit trail but were not entered into pairwise Hedges’ g construction or NMA estimation |
| Certainty implication | Exclusion of otherwise relevant reports because of unavailable compatible numerical data was treated as a potential data-availability/reporting-bias concern in the GRADE/CINeMA-informed certainty assessment |

**Supplementary Table S4B-2. Author-contact and missing-data status statement**

| Reporting item | Final statement |
| --- | --- |
| Author contact for missing numerical data | Study authors were not contacted for missing numerical data for the analyses reported in this review. |
| Unpublished numerical data | No unpublished arm-level numerical data were obtained or incorporated. |
| Reports without compatible data | Reports lacking extractable or derivable arm-level post-intervention n, mean, and SD were retained in the full-text audit trail but were not entered into Hedges' g construction or NMA estimation. |
| Effect-size construction | No missing post-intervention means, SDs, or sample sizes were imputed. |
| Certainty implication | Data-availability limitations were considered in the conservative GRADE/CINeMA-informed certainty assessment. |

Supplementary Table S4B-3. Final report-level audit of reports not entering NMA effect-size estimation because compatible numerical data were unavailable

| Study/report ID | Missing compatible quantitative input | Derivation status | Author-contact status | Final synthesis status |
| --- | --- | --- | --- | --- |
| Toulotte_2012_WiiFit_APA | Post-intervention arm-level n, mean, and SD for the prioritized eligible outcome, or compatible derivable effect data | Not derivable from available report data | Not contacted; not applicable dates/reminders/responses | Not entered into NMA effect-size estimation; no unpublished data incorporated |
| Barker_2015_Pilates_Pilot | Post-intervention arm-level n, mean, and SD for the prioritized eligible outcome, or compatible derivable effect data | Not derivable from available report data | Not contacted; not applicable dates/reminders/responses | Not entered into NMA effect-size estimation; no unpublished data incorporated |
| Yesilyaprak_2016_VR_NursingHome | Post-intervention arm-level n, mean, and SD for the prioritized eligible outcome, or compatible derivable effect data | Not derivable from available report data | Not contacted; not applicable dates/reminders/responses | Not entered into NMA effect-size estimation; no unpublished data incorporated |
| PilatesSuspension_Gait | Post-intervention arm-level n, mean, and SD for the prioritized eligible outcome, or compatible derivable effect data | Not derivable from available report data | Not contacted; not applicable dates/reminders/responses | Not entered into NMA effect-size estimation; no unpublished data incorporated |
| OnlinePilatesSuspension_FearOfFalling | Post-intervention arm-level n, mean, and SD for the prioritized eligible outcome, or compatible derivable effect data | Not derivable from available report data | Not contacted; not applicable dates/reminders/responses | Not entered into NMA effect-size estimation; no unpublished data incorporated |
| DaSilva_2022_Pilates_OlderAdults | Post-intervention arm-level n, mean, and SD for the prioritized eligible outcome, or compatible derivable effect data | Not derivable from available report data | Not contacted; not applicable dates/reminders/responses | Not entered into NMA effect-size estimation; no unpublished data incorporated |
| Sadeghi_2021_BT_VR_MIX | Post-intervention arm-level n, mean, and SD for the prioritized eligible outcome, or compatible derivable effect data | Not derivable from available report data | Not contacted; not applicable dates/reminders/responses | Not entered into NMA effect-size estimation; no unpublished data incorporated |
| DlugoszBos_2021_Pilates_OlderWomen | Post-intervention arm-level n, mean, and SD for the prioritized eligible outcome, or compatible derivable effect data | Not derivable from available report data | Not contacted; not applicable dates/reminders/responses | Not entered into NMA effect-size estimation; no unpublished data incorporated |
| HomeBasedPilates_MS | Post-intervention arm-level n, mean, and SD for the prioritized eligible outcome, or compatible derivable effect data | Not derivable from available report data | Not contacted; not applicable dates/reminders/responses | Not entered into NMA effect-size estimation; no unpublished data incorporated |
| ZahedianNasab_2021_Xbox_Elderly | Post-intervention arm-level n, mean, and SD for the prioritized eligible outcome, or compatible derivable effect data | Not derivable from available report data | Not contacted; not applicable dates/reminders/responses | Not entered into NMA effect-size estimation; no unpublished data incorporated |
| Yuan_2020_IVGB_PD_Crossover | Post-intervention arm-level n, mean, and SD for the prioritized eligible outcome, or compatible derivable effect data | Not derivable from available report data | Not contacted; not applicable dates/reminders/responses | Not entered into NMA effect-size estimation; no unpublished data incorporated |
| Ditchburn_2020_Exergaming_ChronicPain | Post-intervention arm-level n, mean, and SD for the prioritized eligible outcome, or compatible derivable effect data | Not derivable from available report data | Not contacted; not applicable dates/reminders/responses | Not entered into NMA effect-size estimation; no unpublished data incorporated |
| Kayama_2014_Kinect_DTTC | Post-intervention arm-level n, mean, and SD for the prioritized eligible outcome, or compatible derivable effect data | Not derivable from available report data | Not contacted; not applicable dates/reminders/responses | Not entered into NMA effect-size estimation; no unpublished data incorporated |
| Rendon_2012_Wii_BalanceBoard | Post-intervention arm-level n, mean, and SD for the prioritized eligible outcome, or compatible derivable effect data | Not derivable from available report data | Not contacted; not applicable dates/reminders/responses | Not entered into NMA effect-size estimation; no unpublished data incorporated |
| Im_2015_3D_AR_Feasibility | Post-intervention arm-level n, mean, and SD for the prioritized eligible outcome, or compatible derivable effect data | Not derivable from available report data | Not contacted; not applicable dates/reminders/responses | Not entered into NMA effect-size estimation; no unpublished data incorporated |
| Ku_2018_3D_AR_RCT | Post-intervention arm-level n, mean, and SD for the prioritized eligible outcome, or compatible derivable effect data | Not derivable from available report data | Not contacted; not applicable dates/reminders/responses | Not entered into NMA effect-size estimation; no unpublished data incorporated |
| Jorgensen_2013_NintendoWii | Post-intervention arm-level n, mean, and SD for the prioritized eligible outcome, or compatible derivable effect data | Not derivable from available report data | Not contacted; not applicable dates/reminders/responses | Not entered into NMA effect-size estimation; no unpublished data incorporated |
| Pata_2014_Pilates_Quasi | Post-intervention arm-level n, mean, and SD for the prioritized eligible outcome, or compatible derivable effect data | Not derivable from available report data | Not contacted; not applicable dates/reminders/responses | Not entered into NMA effect-size estimation; no unpublished data incorporated |
| Josephs_2016_Pilates_Traditional | Post-intervention arm-level n, mean, and SD for the prioritized eligible outcome, or compatible derivable effect data | Not derivable from available report data | Not contacted; not applicable dates/reminders/responses | Not entered into NMA effect-size estimation; no unpublished data incorporated |
| Vieira_2016_PilatesInspired | Post-intervention arm-level n, mean, and SD for the prioritized eligible outcome, or compatible derivable effect data | Not derivable from available report data | Not contacted; not applicable dates/reminders/responses | Not entered into NMA effect-size estimation; no unpublished data incorporated |
| Bateni_2012_WiiFit_Preliminary | Post-intervention arm-level n, mean, and SD for the prioritized eligible outcome, or compatible derivable effect data | Not derivable from available report data | Not contacted; not applicable dates/reminders/responses | Not entered into NMA effect-size estimation; no unpublished data incorporated |
| Gabizon_2016_Pilates | Post-intervention arm-level n, mean, and SD for the prioritized eligible outcome, or compatible derivable effect data | Not derivable from available report data | Not contacted; not applicable dates/reminders/responses | Not entered into NMA effect-size estimation; no unpublished data incorporated |
| Oliveira_2015_Pilates_Stretching | Post-intervention arm-level n, mean, and SD for the prioritized eligible outcome, or compatible derivable effect data | Not derivable from available report data | Not contacted; not applicable dates/reminders/responses | Not entered into NMA effect-size estimation; no unpublished data incorporated |
| GaticaRojas_2025_Wii_TR_FF | Post-intervention arm-level n, mean, and SD for the prioritized eligible outcome, or compatible derivable effect data | Not derivable from available report data | Not contacted; not applicable dates/reminders/responses | Not entered into NMA effect-size estimation; no unpublished data incorporated |
| Mat_Reformer_Pilates_MS | Post-intervention arm-level n, mean, and SD for the prioritized eligible outcome, or compatible derivable effect data | Not derivable from available report data | Not contacted; not applicable dates/reminders/responses | Not entered into NMA effect-size estimation; no unpublished data incorporated |
| Szturm_2011_ComputerGame | Post-intervention arm-level n, mean, and SD for the prioritized eligible outcome, or compatible derivable effect data | Not derivable from available report data | Not contacted; not applicable dates/reminders/responses | Not entered into NMA effect-size estimation; no unpublished data incorporated |
| Donath_2016_Pilates_Balance_Control | Post-intervention arm-level n, mean, and SD for the prioritized eligible outcome, or compatible derivable effect data | Not derivable from available report data | Not contacted; not applicable dates/reminders/responses | Not entered into NMA effect-size estimation; no unpublished data incorporated |
| Park_2013_VR_Stroke | Post-intervention arm-level n, mean, and SD for the prioritized eligible outcome, or compatible derivable effect data | Not derivable from available report data | Not contacted; not applicable dates/reminders/responses | Not entered into NMA effect-size estimation; no unpublished data incorporated |
| Padala_2017_WiiFit | Post-intervention arm-level n, mean, and SD for the prioritized eligible outcome, or compatible derivable effect data | Not derivable from available report data | Not contacted; not applicable dates/reminders/responses | Not entered into NMA effect-size estimation; no unpublished data incorporated |
| Bieryla_2013_WiiFit | Post-intervention arm-level n, mean, and SD for the prioritized eligible outcome, or compatible derivable effect data | Not derivable from available report data | Not contacted; not applicable dates/reminders/responses | Not entered into NMA effect-size estimation; no unpublished data incorporated |
| Lim_2024_Telerehab_ACL | Post-intervention arm-level n, mean, and SD for the prioritized eligible outcome, or compatible derivable effect data | Not derivable from available report data | Not contacted; not applicable dates/reminders/responses | Not entered into NMA effect-size estimation; no unpublished data incorporated |
| Greco_2025_VR_Traditional_Females | Post-intervention arm-level n, mean, and SD for the prioritized eligible outcome, or compatible derivable effect data | Not derivable from available report data | Not contacted; not applicable dates/reminders/responses | Not entered into NMA effect-size estimation; no unpublished data incorporated |
| deVries_2018_Exergame | Post-intervention arm-level n, mean, and SD for the prioritized eligible outcome, or compatible derivable effect data | Not derivable from available report data | Not contacted; not applicable dates/reminders/responses | Not entered into NMA effect-size estimation; no unpublished data incorporated |
| Mesquita_2015_PNF_Pilates_Control | Post-intervention arm-level n, mean, and SD for the prioritized eligible outcome, or compatible derivable effect data | Not derivable from available report data | Not contacted; not applicable dates/reminders/responses | Not entered into NMA effect-size estimation; no unpublished data incorporated |
| Shih_2016_Kinect_PD | Post-intervention arm-level n, mean, and SD for the prioritized eligible outcome, or compatible derivable effect data | Not derivable from available report data | Not contacted; not applicable dates/reminders/responses | Not entered into NMA effect-size estimation; no unpublished data incorporated |
| Barry_2016_Xbox_HealthyAdults | Post-intervention arm-level n, mean, and SD for the prioritized eligible outcome, or compatible derivable effect data | Not derivable from available report data | Not contacted; not applicable dates/reminders/responses | Not entered into NMA effect-size estimation; no unpublished data incorporated |
| Fakhro_2019_WiiFit_OlderAdults | Post-intervention arm-level n, mean, and SD for the prioritized eligible outcome, or compatible derivable effect data | Not derivable from available report data | Not contacted; not applicable dates/reminders/responses | Not entered into NMA effect-size estimation; no unpublished data incorporated |
| Liao_2019_Kinect_Frailty | Post-intervention arm-level n, mean, and SD for the prioritized eligible outcome, or compatible derivable effect data | Not derivable from available report data | Not contacted; not applicable dates/reminders/responses | Not entered into NMA effect-size estimation; no unpublished data incorporated |

**Supplementary Table S4C. Operational definitions of final allocation categories**

| **Final revised allocation category** | **Operational definition** | **Required condition** | **Revised synthesis role** |
| --- | --- | --- | --- |
| Included in primary randomized-trial NMA | Source-verified randomized controlled trial with an eligible balance or postural-control outcome and compatible post-intervention arm-level n, mean, and SD | Random allocation confirmed; intervention arms linkable to the final network taxonomy; prioritized eligible outcome available | Main inferential synthesis |
| Included in broader randomized-trial NMA only | Source-verified randomized controlled trial with compatible quantitative data but assigned to broader-layer evidence because it used broader-layer evidence defined in the final analytical amendment or fall-related proxy outcome | Random allocation confirmed; compatible numerical data available; primary-layer criteria not met | Sensitivity evidence layer |
| Nonrandomized controlled evidence outside randomized-trial NMA | Controlled intervention study with nonrandom allocation, such as first-come, first-served allocation | Allocation procedure not compatible with randomized-trial primary inference | Contextual nonrandomized controlled evidence only; not used for primary conclusions |
| Qualitative/contextual evidence only | Report relevant to Pilates, technology-assisted exercise, digital exercise, telerehabilitation, balance training, postural control, or fall-related rehabilitation but not eligible for NMA effect-size estimation | Relevant to review scope but not entered into quantitative network because of design, data-format, node-linkage, or contextual-only status | Contextual synthesis |
| Excluded from NMA effect-size estimation because compatible quantitative input was unavailable or not derivable | Report lacked compatible post-intervention arm-level n, mean, and SD for the prioritized eligible outcome, and values could not be derived without unverifiable assumptions | Missing compatible numerical input for Hedges’ g construction | Retained in full-text audit trail; linked to Supplementary Table S4B |
| Excluded after full-text assessment for other PICOS reasons | Report failed one or more core eligibility criteria unrelated to numerical data availability | Ineligible population, intervention, comparator, outcome, design, or non-linkable evidence structure | Not entered into NMA; retained in audit trail |
| Final unresolved/pending | No report remained unresolved after final full-text closure | Not applicable | Audit confirmation only |

Supplementary Table S4D. Selected allocation amendments and source-verification decisions

| Study/report ID or decision group | **Final allocation** | **Final revised allocation** | Reason for final allocation or confirmation | **Final role in revised synthesis** |
| --- | --- | --- | --- | --- |
| Kalron_2016 | Primary NMA; broader/sensitivity NMA | Included in primary randomized-trial NMA | Source-verified randomized trial with compatible arm-level post-intervention data for a prioritized balance/postural-control outcome | Main inferential evidence base |
| Duff_2017_Pilates_MS | Primary NMA; broader/sensitivity NMA | Included in primary randomized-trial NMA | Source-verified randomized trial with compatible arm-level post-intervention data; also informative for control-node sensitivity and Pilates-control direct evidence | Main inferential evidence base |
| Lee_2023_RingFit | Primary NMA; broader/sensitivity NMA | Included in primary randomized-trial NMA | Source-verified randomized trial with compatible arm-level post-intervention data | Main inferential evidence base |
| Eldemir_2024 | Primary NMA; broader/sensitivity NMA | Included in primary randomized-trial NMA | Source-verified randomized trial with compatible arm-level post-intervention data | Main inferential evidence base |
| Hadas_2024 | Primary NMA; broader/sensitivity NMA | Included in primary randomized-trial NMA | Source-verified randomized trial with compatible arm-level post-intervention data for technology-delivered Pilates versus face-to-face Pilates comparison | Main inferential evidence base |
| Abdelraouf_2020 | Primary NMA; broader/sensitivity NMA | Included in primary randomized-trial NMA | Source-verified randomized trial with compatible arm-level post-intervention data; retained with explicit transitivity concern because the population consisted of athletes with nonspecific low back pain | Main inferential evidence base; evaluated in population-sensitivity analysis |
| Yen_2011_VR_PD | Primary NMA; broader/sensitivity NMA | Included in primary randomized-trial NMA | Source-verified randomized trial with compatible arm-level post-intervention data | Main inferential evidence base |
| Molhemi_2021_VR_MS | Primary NMA; broader/sensitivity NMA | Included in primary randomized-trial NMA | Source-verified randomized trial with compatible arm-level post-intervention data | Main inferential evidence base |
| Junata_2021 | Primary NMA; broader/sensitivity NMA | Included in primary randomized-trial NMA | Source-verified randomized trial with compatible arm-level post-intervention data | Main inferential evidence base |
| Lee_and_Yoo_2017 | Primary NMA; broader/sensitivity NMA | Included in primary randomized-trial NMA with same-node arm combination | Source-verified randomized trial; Yoga and Self-Otago were both coded as conventional exercise and were combined into one conventional-exercise analytic arm before contrast construction | Main inferential evidence base; source-arm and analytic-arm accounting documented in Supplementary Tables S7–S8 |
| Bacha_2018_Kinect | Primary NMA; broader/sensitivity NMA | Included in primary randomized-trial NMA | Source-verified randomized trial with compatible arm-level post-intervention data | Main inferential evidence base |
| Sato_2015 | Primary NMA; broader/sensitivity NMA | Included in primary randomized-trial NMA | Source-verified randomized trial with compatible arm-level post-intervention data | Main inferential evidence base |
| AibarAlmazan_2019 | Broader/sensitivity NMA only | Included in broader randomized-trial NMA only | Randomized broader-layer evidence with compatible quantitative input but allocated outside the primary layer because it used a fall-related proxy or broader-layer outcome | Broader randomized-trial sensitivity layer |
| Prasertsakul_2018_VR_Pilot | Broader/sensitivity NMA only | Included in broader randomized-trial NMA only | Randomized broader-layer/pilot evidence with compatible quantitative input | Broader randomized-trial sensitivity layer |
| Sapi_2019 | Primary NMA; broader/sensitivity NMA | Nonrandomized controlled evidence outside randomized-trial NMA | Source verification identified first-come, first-served allocation rather than random allocation; therefore it was not compatible with randomized-trial primary inference | Contextual nonrandomized controlled evidence; not used to determine primary conclusions |
| Yang_2020_Kinect_older_adults | Qualitative/contextual evidence only | Qualitative/contextual evidence only | Relevant to the review question and technology landscape but not entered into effect-size estimation because compatible quantitative input for the final network was not available | Contextual evidence only |
| Bird_2012_Pilates_older_adults | Qualitative/contextual evidence only | Qualitative/contextual evidence only | Relevant to Pilates and balance-oriented exercise but not entered into NMA effect-size estimation | Contextual evidence only |
| Rezaei_2019_Cervigame_neck_pain | Qualitative/contextual evidence only | Qualitative/contextual evidence only | Relevant to digital/technology-supported motor-control rehabilitation but not entered into NMA effect-size estimation | Contextual evidence only |
| Cetin_2022_VR_neck_pain | Qualitative/contextual evidence only | Qualitative/contextual evidence only | Relevant to technology-supported rehabilitation but not entered into NMA effect-size estimation | Contextual evidence only |
| Toulotte_2012_WiiFit_APA | Excluded from quantitative synthesis: no extractable/derivable arm-level post mean/SD | Excluded from NMA effect-size estimation because compatible quantitative input was unavailable or not derivable | Compatible post-intervention arm-level mean and SD for the prioritized eligible outcome were not reported and could not be derived from the available source material | Retained in full-text audit trail; linked to Supplementary Table S4B |
| Barker_2015_Pilates_Pilot | Excluded from quantitative synthesis: no extractable/derivable arm-level post mean/SD | Excluded from NMA effect-size estimation because compatible quantitative input was unavailable or not derivable | Compatible post-intervention arm-level mean and SD were unavailable or not derivable | Retained in full-text audit trail; linked to Supplementary Table S4B |
| Yesilyaprak_2016_VR_NursingHome | Excluded from quantitative synthesis: no extractable/derivable arm-level post mean/SD | Excluded from NMA effect-size estimation because compatible quantitative input was unavailable or not derivable | Compatible post-intervention arm-level mean and SD were unavailable or not derivable | Retained in full-text audit trail; linked to Supplementary Table S4B |
| ZahedianNasab_2021_Xbox_Elderly | Excluded from quantitative synthesis: no extractable/derivable arm-level post mean/SD | Excluded from NMA effect-size estimation because compatible quantitative input was unavailable or not derivable | Compatible post-intervention arm-level mean and SD were unavailable or not derivable | Retained in full-text audit trail; linked to Supplementary Table S4B |
| Yuan_2020_IVGB_PD_Crossover | Excluded from quantitative synthesis: no extractable/derivable arm-level post mean/SD | Excluded from NMA effect-size estimation because compatible quantitative input was unavailable or not derivable | Compatible post-intervention arm-level mean and SD were unavailable or not derivable | Retained in full-text audit trail; linked to Supplementary Table S4B |
| Park_2013_VR_Stroke | Excluded from quantitative synthesis: no extractable/derivable arm-level post mean/SD | Excluded from NMA effect-size estimation because compatible quantitative input was unavailable or not derivable | Compatible post-intervention arm-level mean and SD were unavailable or not derivable | Retained in full-text audit trail; linked to Supplementary Table S4B |
| Padala_2017_WiiFit | Excluded from quantitative synthesis: no extractable/derivable arm-level post mean/SD | Excluded from NMA effect-size estimation because compatible quantitative input was unavailable or not derivable | Compatible post-intervention arm-level mean and SD were unavailable or not derivable | Retained in full-text audit trail; linked to Supplementary Table S4B |
| Bieryla_2013_WiiFit | Excluded from quantitative synthesis: no extractable/derivable arm-level post mean/SD | Excluded from NMA effect-size estimation because compatible quantitative input was unavailable or not derivable | Compatible post-intervention arm-level mean and SD were unavailable or not derivable | Retained in full-text audit trail; linked to Supplementary Table S4B |

Note. NMA, network meta-analysis; RCT, randomized controlled trial; SD, standard deviation. This table reports the final full-text allocation audit and the final handling of reports that did not enter NMA effect-size estimation. Study authors were not contacted for missing numerical data, no unpublished arm-level numerical data were obtained, and no unavailable numerical values were imputed.

**Supplementary Table S5. Study-design verification, allocation classification, and risk-of-bias-tool compatibility**

Supplementary Table S5A. Purpose and final decision rules

| **Verification item** | Decision rule used in the final review | **Consequence for evidence allocation** |
| --- | --- | --- |
| Source-verified randomized allocation | The source report described random assignment, random allocation, random sequence generation, or another allocation procedure compatible with randomized-trial inference | Eligible for the primary or broader randomized-trial NMA if all PICOS, node-coding, outcome, and data-format criteria were also met |
| Nonrandom allocation | The source report described first-come, first-served allocation, sequential allocation, convenience allocation, participant preference, or another allocation procedure not compatible with randomized-trial inference | Excluded from the primary and broader randomized-trial NMA layers; retained outside the randomized-trial evidence base as contextual nonrandomized controlled evidence |
| Unclear allocation | The source report did not provide sufficient information to confirm randomized allocation | Classified conservatively and reflected in risk-of-bias and certainty judgments; not upgraded to randomized-trial status without source support |
| Same-node arms within a randomized trial | More than one source arm in the same randomized trial represented the same network node | Same-node arms were combined before contrast construction using standard arm-combination formulae |
| RoB2 compatibility | The study was a source-verified randomized trial contributing to the primary or broader randomized-trial NMA | Assessed using RoB2 and incorporated into randomized-trial certainty judgments |
| Nonrandomized controlled evidence | The study used a controlled but nonrandom allocation procedure | Not assessed using RoB2 for randomized-trial inference and not used to determine primary randomized-trial certainty conclusions |
| Assessment workflow | Study-design classification and risk-of-bias-tool compatibility were reviewed using an author/verifier workflow one reviewer assessed and a second reviewer verified | The workflow was reported transparently to avoid overstating independent duplication |

**Supplementary Table S5B. Study-level allocation verification and revised synthesis role**

| **Study/report ID** | **Population/context** | **Source-verified allocation classification** | **Randomized allocation confirmed?** | **Revised evidence-layer allocation** | **Reason for revised allocation** | **Risk-of-bias handling for revised synthesis** |
| --- | --- | --- | --- | --- | --- | --- |
| Kalron_2016 | People with multiple sclerosis; balance/postural-control rehabilitation | Randomized controlled trial | Yes | Primary randomized-trial NMA | Source-verified randomized trial with compatible arm-level post-intervention data for a prioritized balance/postural-control outcome | RoB2 |
| Duff_2017_Pilates_MS | People with multiple sclerosis; Pilates or comparator rehabilitation | Randomized controlled trial | Yes | Primary randomized-trial NMA | Source-verified randomized trial with compatible arm-level post-intervention data; also informative for control-node sensitivity and Pilates-control direct evidence | RoB2 |
| Lee_2023_RingFit | Community-dwelling older adults; Ring Fit or active video-game technology-assisted exercise compared with education-only/no structured exercise control | Randomized controlled trial | Yes | Primary randomized-trial NMA | Source-verified randomized trial with compatible arm-level post-intervention data for a prioritized balance-performance outcome. Home-based digital delivery and control subtype were considered in certainty interpretation | RoB2 |
| Eldemir_2024 | People with multiple sclerosis; technology-delivered Pilates compared with waitlist/no active Pilates telerehabilitation control | Randomized controlled trial | Yes | Primary randomized-trial NMA | Source-verified randomized trial with compatible arm-level post-intervention data for a prioritized postural-control outcome | RoB2 |
| Hadas_2024 | Healthy sedentary women; remote technology-delivered Pilates compared with in-studio face-to-face Pilates | Randomized controlled intervention | Yes | Primary randomized-trial NMA | Source-verified randomized study with compatible arm-level post-intervention data. The study informed the technology-delivered Pilates versus face-to-face Pilates comparison and was interpreted cautiously because delivery mode, supervision, adherence, and implementation context may differ | RoB2 |
| Abdelraouf_2020 | Athletes with nonspecific low back pain; virtual-reality or technology-assisted exercise compared with conventional core-stability exercise | Randomized clinical trial | Yes | Primary randomized-trial NMA | Source-verified randomized trial with compatible quantitative data; retained in the primary randomized-trial network but explicitly flagged as a transitivity concern because the athletic low-back-pain population differed from older-adult and neurological rehabilitation samples | RoB2 |
| Yen_2011_VR_PD | People with Parkinson disease; virtual-reality exercise, conventional exercise, and no formal balance/physical-therapy control | Randomized controlled trial | Yes | Primary randomized-trial NMA | Source-verified randomized trial with compatible arm-level post-intervention data; multi-arm structure handled within the network model | RoB2 |
| Molhemi_2021_VR_MS | People with multiple sclerosis; virtual-reality exercise compared with conventional balance/rehabilitation programme | Randomized controlled trial | Yes | Primary randomized-trial NMA | Source-verified randomized trial with compatible arm-level post-intervention data for a prioritized balance-performance outcome | RoB2 |
| Junata_2021 | Chronic stroke survivors; Kinect or technology-assisted balance training compared with conventional balance training | Assessor-blinded randomized controlled trial | Yes | Primary randomized-trial NMA | Source-verified randomized trial with compatible arm-level post-intervention data; neurological rehabilitation context considered in transitivity assessment | RoB2 |
| Lee_and_Yoo_2017 | Elderly women; AR-Otago, Yoga, and Self-Otago exercise arms | Three-arm randomized controlled trial | Yes | Primary randomized-trial NMA with same-node arm combination | Source-verified randomized trial. Yoga and Self-Otago were both coded as conventional exercise and were combined into one conventional-exercise analytic arm before contrast construction | RoB2 |
| Bacha_2018_Kinect | Community-dwelling older adults; Kinect-based technology-assisted training compared with conventional physical therapy | Randomized clinical trial | Yes | Primary randomized-trial NMA | Source-verified randomized trial with compatible arm-level post-intervention data for a prioritized balance-performance outcome. Active conventional physical-therapy comparator content was considered in transitivity and certainty interpretation | RoB2 |
| Sato_2015 | Healthy elderly adults; interactive video-game or Kinect-type technology-assisted exercise compared with usual-activity/no structured exercise control | Randomized controlled trial | Yes | Primary randomized-trial NMA | Source-verified randomized trial with compatible arm-level post-intervention data for a prioritized balance-performance outcome. Usual-activity control subtype was considered in the control-node audit and certainty assessment | RoB2 |
| AibarAlmazan_2019 | Community-dwelling elderly women; Pilates compared with no formal intervention control using fall-related proxy evidence | Randomized controlled trial | Yes | Broader randomized-trial NMA only | Source-verified randomized study with compatible quantitative input but allocated to the broader layer because the selected outcome was a fall-related proxy rather than a primary balance/postural-control endpoint | RoB2 |
| Prasertsakul_2018_VR_Pilot | Healthy adults; virtual-reality or technology-assisted postural-control training compared with conventional balance training | Randomized pilot/preliminary controlled study | Yes | Broader randomized-trial NMA only | Source-verified randomized broader/pilot evidence with compatible quantitative input; retained only in the broader randomized-trial network because of small pilot evidence and broader-layer status | RoB2 |
| Sapi_2019 | Older adults; controlled technology-assisted exercise evidence | Nonrandomized controlled allocation; first-come, first-served allocation | No | Nonrandomized controlled evidence outside randomized-trial NMA | Allocation was not compatible with randomized-trial primary inference. The study was excluded from the primary and broader randomized-trial NMA layers and was not used to determine the primary randomized-trial conclusions | Not assessed using RoB2 for randomized-trial inference; retained outside the randomized-trial certainty framework |

**Supplementary Table S5C. Revised synthesis totals derived from source-verified allocation classification**

| **Revised evidence layer** | **Studies/reports** | **Randomized/source arms** | **Analytic arms** | **Participants** | **Study-design basis** | **Risk-of-bias framework** |
| --- | --- | --- | --- | --- | --- | --- |
| Primary randomized-trial NMA | 12 studies | 26 randomized source arms | 25 analytic arms | 491 | Source-verified randomized controlled trials | RoB2 |
| Broader randomized-trial NMA | 14 studies | 30 randomized source arms | 29 analytic arms | 606 | Source-verified randomized controlled trials, including the 12 primary randomized studies plus 2 broader-only randomized studies | RoB2 |
| Nonrandomized controlled evidence outside randomized-trial networks | 1 study | Not included in randomized-trial arm accounting | Not included in randomized-trial arm accounting | Not included in randomized-trial totals | Nonrandom controlled allocation | Retained outside randomized-trial certainty conclusions |
| Qualitative/contextual evidence only | 13 reports | Not applicable | Not applicable | Not applicable | Contextual evidence | Not applicable to NMA risk-of-bias synthesis |
| Reports excluded from NMA effect-size estimation because compatible data were unavailable or not derivable | 38 reports | Not applicable | Not applicable | Not applicable | Not entered into NMA | Considered under data-availability/reporting-bias concerns where relevant |

**Supplementary Table S5D. Risk-of-bias-tool compatibility and certainty-use rules**

| **Evidence type** | **Risk-of-bias tool or handling** | **Used in primary randomized-trial certainty conclusions?** | **Rationale** |
| --- | --- | --- | --- |
| Source-verified randomized trials in the primary randomized-trial NMA | RoB2 | Yes | Compatible with randomized-trial inference and primary network estimates |
| Source-verified randomized trials included only in the broader randomized-trial NMA | RoB2 | Used for broader/sensitivity interpretation, not as replacement for primary inference | Included to examine directional sensitivity after adding broader-layer randomized evidence |
| Nonrandomized controlled evidence outside randomized-trial NMA | Not assessed using RoB2 for randomized-trial inference | No | Nonrandom allocation is not compatible with RoB2-based randomized-trial certainty judgments |
| Contextual reports not entering NMA | Not applicable to NMA certainty | No | Used only for contextual evidence, implementation discussion, or evidence-landscape description |
| Reports without compatible numerical data | Not applicable to direct effect-size RoB contribution | No direct contribution to NMA effect estimates | Considered qualitatively under data-availability/reporting-bias concerns |

**Supplementary Table S5E. Operational workflow for study-design verification and risk-of-bias judgments**

| Step | Procedure used in the final review |
| --- | --- |
| 1. Study-design verification | Allocation procedures and analysis-layer eligibility were checked against the source reports and the locked extraction dataset. |
| 2. Randomized-trial restriction | The primary inferential analysis was restricted to source-verified randomized trials with eligible balance/postural-control outcomes and compatible arm-level data. |
| 3. RoB2 scope | RoB2 domain-level and overall judgments were completed for all 14 source-verified randomized trials contributing to either randomized-trial evidence layer. Twelve trials contributed to the primary network, and two contributed only to the broader randomized-trial sensitivity network. |
| 4. Nonrandomized controlled evidence | The controlled study with nonrandom first-come, first-served allocation was retained outside both randomized-trial networks and was not assessed using RoB2 for randomized-trial inference. |
| 5. Certainty linkage | Primary comparison-level certainty ratings were based on the 12-study primary randomized-trial network and informed by its evidence structure. |

Note. AR, augmented reality; NMA, network meta-analysis; RCT, randomized controlled trial; RoB, risk of bias; RoB2, revised Cochrane risk-of-bias tool for randomized trials. The primary and broader randomized-trial networks were restricted to source-verified randomized trials. One controlled study using nonrandom allocation was retained outside the randomized-trial networks and was not used to determine the primary conclusions. RoB2 judgments were applied to randomized trials only. The study-design and risk-of-bias workflow was reported transparently as an author/verifier model one reviewer assessed and a second reviewer verified. Study descriptions in this table were aligned with the revised arm-level extraction, node coding, outcome-domain, control-node, and certainty-assessment tables.

**Supplementary Table S6. Study characteristics and revised quantitative evidence allocation**

**Supplementary Table S6A. Studies included in the primary randomized-trial network meta-analysis**

| **No.** | **Study/report ID** | **Population/condition** | **Source-verified design** | **Participants in revised primary RCT network** | **Source arms** | **Analytic arms** | **Intervention node(s) represented** | **Comparator node(s) represented** | **Outcome used in revised NMA** | **Outcome domain** | **Revised analysis layer** | **Key allocation note** |
| --- | --- | --- | --- | --- | --- | --- | --- | --- | --- | --- | --- | --- |
| 1 | Abdelraouf_2020 | Athletes with nonspecific low back pain | Randomized clinical trial | 50 | 2 | 2 | Technology-assisted exercise | Conventional exercise | SEBT posteromedial reach | Balance performance | Primary randomized-trial NMA; broader randomized-trial NMA | Retained in the primary RCT network, but explicitly flagged in the structured transitivity assessment because athletic low-back-pain participants differed from older-adult and neurological rehabilitation samples |
| 2 | Bacha_2018_Kinect | Community-dwelling older adults | Randomized clinical trial | 46 | 2 | 2 | Technology-assisted exercise | Conventional exercise | Mini-BESTest | Balance performance | Primary randomized-trial NMA; broader randomized-trial NMA | Eligible RCT contributing direct technology-assisted exercise versus conventional exercise evidence |
| 3 | Duff_2017_Pilates_MS | People with multiple sclerosis | Randomized controlled trial | 30 | 2 | 2 | Pilates | Control | FABS | Balance performance | Primary randomized-trial NMA; broader randomized-trial NMA | Eligible RCT contributing direct Pilates versus control evidence; also relevant to control-node sensitivity and Pilates-control direct–indirect evidence assessment |
| 4 | Eldemir_2024 | People with multiple sclerosis | Randomized controlled trial | 30 | 2 | 2 | Technology-delivered Pilates | Control | LOS | Instrumented/laboratory postural control | Primary randomized-trial NMA; broader randomized-trial NMA | Eligible RCT contributing technology-delivered Pilates versus control evidence |
| 5 | Hadas_2024 | Healthy sedentary women | Randomized controlled intervention | 38 | 2 | 2 | Technology-delivered Pilates | Pilates | Stork test | Balance performance | Primary randomized-trial NMA; broader randomized-trial NMA | Eligible randomized study contributing direct technology-delivered Pilates versus face-to-face Pilates evidence |
| 6 | Junata_2021 | Chronic stroke survivors | Assessor-blinded randomized controlled trial | 30 | 2 | 2 | Technology-assisted exercise | Conventional exercise | BBS | Balance performance | Primary randomized-trial NMA; broader randomized-trial NMA | Eligible RCT contributing technology-assisted exercise versus conventional exercise evidence in a neurological rehabilitation context |
| 7 | Kalron_2016 | People with multiple sclerosis | Assessor-blinded randomized controlled trial | 45 | 2 | 2 | Pilates | Conventional exercise | BBS | Balance performance | Primary randomized-trial NMA; broader randomized-trial NMA | Eligible RCT contributing direct Pilates versus conventional exercise evidence |
| 8 | Lee_and_Yoo_2017 | Elderly women | Three-arm randomized controlled trial | 30 | 3 | 2 | Technology-assisted exercise | Conventional exercise | EO CoP-x | Instrumented/laboratory postural control | Primary randomized-trial NMA; broader randomized-trial NMA | Three randomized source arms were available. Yoga and Self-Otago were both coded as conventional exercise and were combined into one conventional-exercise analytic arm before contrast construction |
| 9 | Lee_2023_RingFit | Community-dwelling older adults | Single-blind randomized controlled trial | 57 | 2 | 2 | Technology-assisted exercise | Control | BBS | Balance performance | Primary randomized-trial NMA; broader randomized-trial NMA | Eligible RCT contributing direct technology-assisted exercise versus control evidence |
| 10 | Molhemi_2021_VR_MS | People with multiple sclerosis | Single-blind randomized controlled trial | 39 | 2 | 2 | Technology-assisted exercise | Conventional exercise | BBS | Balance performance | Primary randomized-trial NMA; broader randomized-trial NMA | Eligible RCT contributing technology-assisted exercise versus conventional exercise evidence |
| 11 | Sato_2015 | Healthy elderly adults | Randomized controlled trial | 54 | 2 | 2 | Technology-assisted exercise | Control | FRT | Balance performance | Primary randomized-trial NMA; broader randomized-trial NMA | Eligible RCT contributing direct technology-assisted exercise versus control evidence |
| 12 | Yen_2011_VR_PD | People with Parkinson disease | Single-blinded randomized controlled trial | 42 | 3 | 3 | Technology-assisted exercise; conventional exercise | Control | SOT-6 single-task equilibrium score | Instrumented/laboratory postural control | Primary randomized-trial NMA; broader randomized-trial NMA | Eligible three-arm RCT contributing direct evidence among technology-assisted exercise, conventional exercise, and control |

**Supplementary Table S6B. Studies included only in the broader randomized-trial network meta-analysis**

| **No.** | **Study/report ID** | **Population/condition** | **Source-verified design** | **Participants in revised broader RCT network** | **Source arms** | **Analytic arms** | **Intervention node(s) represented** | **Comparator node(s) represented** | **Outcome used in revised NMA** | **Outcome domain** | **Revised analysis layer** | **Key allocation note** |
| --- | --- | --- | --- | --- | --- | --- | --- | --- | --- | --- | --- | --- |
| 13 | AibarAlmazan_2019 | Community-dwelling elderly women | Randomized controlled trial | 107 | 2 | 2 | Pilates | Control | FES-I | Fall-related proxy | Broader randomized-trial NMA only | Randomized broader-layer evidence retained only in the broader network because the prioritized quantitative outcome was a fall-related proxy rather than a primary balance/postural-control endpoint |
| 14 | Prasertsakul_2018_VR_Pilot | Healthy adults | Randomized pilot/preliminary study | 8 | 2 | 2 | Technology-assisted exercise | Conventional exercise | CoP sway area, eyes closed | Instrumented/laboratory postural control | Broader randomized-trial NMA only | Randomized pilot/broader-layer evidence retained only in the broader randomized-trial sensitivity network |

**Supplementary Table S6C. Nonrandomized controlled evidence retained outside the randomized-trial networks**

| **No.** | **Study/report ID** | **Population/condition** | **Source-verified design** | **Source-report participants** | **Previously extracted comparison** | **Outcome considered in previous extraction** | **Revised allocation** | **Reason not included in randomized-trial NMA** | **Use in revised manuscript** |
| --- | --- | --- | --- | --- | --- | --- | --- | --- | --- |
| 15 | Sapi_2019 | Older adults | Nonrandomized controlled intervention; first-come, first-served allocation | 53 | Technology-assisted exercise versus conventional exercise | LOS movement velocity | Nonrandomized controlled evidence outside the randomized-trial NMA | Allocation procedure was not compatible with randomized-trial primary inference. The study was excluded from the primary and broader randomized-trial NMA layers | Contextual/nonrandomized controlled evidence only; not used to determine primary RCT estimates or primary RCT certainty conclusions |

**Supplementary Table S6D. Revised quantitative evidence accounting**

| **Evidence layer** | **Studies** | **Source arms** | **Analytic arms** | **Participants** | **Included studies** |
| --- | --- | --- | --- | --- | --- |
| Primary randomized-trial NMA | 12 | 26 | 25 | 491 | Abdelraouf_2020; Bacha_2018_Kinect; Duff_2017_Pilates_MS; Eldemir_2024; Hadas_2024; Junata_2021; Kalron_2016; Lee_and_Yoo_2017; Lee_2023_RingFit; Molhemi_2021_VR_MS; Sato_2015; Yen_2011_VR_PD |
| Broader randomized-trial NMA | 14 | 30 | 29 | 606 | All 12 primary randomized-trial studies plus AibarAlmazan_2019 and Prasertsakul_2018_VR_Pilot |
| Nonrandomized controlled evidence outside randomized-trial networks | 1 | Not included in randomized-trial arm accounting | Not included in randomized-trial arm accounting | Not included in randomized-trial totals | Sapi_2019 |
| Qualitative/contextual reports only | 13 reports | Not applicable | Not applicable | Not applicable | Listed in Supplementary Table S4 |
| Reports excluded from NMA effect-size estimation because compatible numerical data were unavailable or not derivable | 38 reports | Not applicable | Not applicable | Not applicable | Listed in Supplementary Tables S4 and S4B |

**Note.** BBS, Berg Balance Scale; CoP, center of pressure; EO CoP-x, eyes-open center-of-pressure displacement in the x-axis; FABS, Fullerton Advanced Balance Scale; FES-I, Falls Efficacy Scale-International; FRT, Functional Reach Test; LOS, limits of stability; Mini-BESTest, Mini Balance Evaluation Systems Test; NMA, network meta-analysis; RCT, randomized controlled trial; SEBT, Star Excursion Balance Test; SOT, Sensory Organization Test. Source arms refer to original randomized study arms before same-node arm combination. Analytic arms refer to arms entered into network contrast construction after same-node combination. The primary randomized-trial NMA included 12 studies, 26 randomized source arms represented as 25 analytic arms, and 491 participants. The broader randomized-trial NMA included 14 randomized studies, 30 randomized source arms represented as 29 analytic arms, and 606 participants. Sapi_2019 was retained outside the randomized-trial networks because its allocation was nonrandom. Risk-of-bias-tool compatibility is reported separately in Supplementary Table S5, and detailed arm-level baseline and post-intervention extraction is reported in Supplementary Table S7.

**Supplementary Table S7. Baseline and post-intervention arm-level extraction dataset for the randomized-trial networks**

**Supplementary Table S7A. Arm-level extraction variables and analysis rules**

| **Field** | **Definition and use in the revised analysis** |
| --- | --- |
| Study/report ID | Unique study identifier used across the revised NMA files |
| Arm ID | Unique analytic arm identifier used in the revised arm-level dataset |
| Source arm | Original randomized study arm before any same-node arm combination |
| Analytic arm | Arm entered into contrast construction after same-node arm combination, where applicable |
| Node | Revised network node: control, conventional exercise, Pilates, technology-assisted exercise, or technology-delivered Pilates |
| Analysis layer | Primary randomized-trial NMA or broader randomized-trial NMA only |
| Outcome | Prioritized eligible outcome used for revised effect-size construction |
| Outcome domain | Balance performance, instrumented/laboratory postural control, or fall-related proxy |
| Baseline n/mean/SD | Arm-level baseline values for the selected outcome, extracted where available |
| Post-intervention n/mean/SD | Arm-level immediate post-intervention values used for Hedges’ g construction |
| Direction harmonization | Outcomes were harmonized so that higher values represented better balance-, postural-control-, or fall-related performance |
| Lower-is-better transformation | For lower-is-better outcomes, mean values were sign-reversed before effect-size construction; SDs were unchanged |
| Same-node arm combination | Same-node arms within the same randomized trial were combined before contrast construction using standard arm-combination formulae |
| Baseline comparability audit | Baseline standardized differences were used descriptively to identify possible selected-outcome baseline imbalance; they were not used as baseline-adjusted NMA estimates |

**Supplementary Table S7B. Analytic arm-level extraction dataset for the primary randomized-trial NMA**

| **Study/report ID** | **Arm ID** | **Arm name** | **Node** | **n** | **Baseline mean** | **Baseline SD** | **Post mean** | **Post SD** | **Outcome** | **Outcome domain** | **Direction harmonization** | **Use in primary RCT NMA** |
| --- | --- | --- | --- | --- | --- | --- | --- | --- | --- | --- | --- | --- |
| Abdelraouf_2020 | Abdelraouf_2020_A | Core stability exercise plus VR | Technology-assisted exercise | 25 | 84.24 | 13.76 | 94.40 | 12.95 | SEBT posteromedial reach | Balance performance | Higher values better; no reversal | Yes |
| Abdelraouf_2020 | Abdelraouf_2020_B | Core stability exercise | Conventional exercise | 25 | 83.44 | 14.15 | 86.40 | 13.39 | SEBT posteromedial reach | Balance performance | Higher values better; no reversal | Yes |
| Bacha_2018_Kinect | Bacha_2018_A | Kinect Adventures training | Technology-assisted exercise | 23 | 26.52 | 3.24 | 29.65 | 2.87 | Mini-BESTest | Balance performance | Higher values better; no reversal | Yes |
| Bacha_2018_Kinect | Bacha_2018_B | Conventional physical therapy | Conventional exercise | 23 | 27.60 | 2.64 | 29.82 | 2.08 | Mini-BESTest | Balance performance | Higher values better; no reversal | Yes |
| Duff_2017_Pilates_MS | Duff_2017_A | Pilates exercise programme | Pilates | 15 | 28.70 | 11.70 | 31.00 | 9.20 | FABS | Balance performance | Higher values better; no reversal | Yes |
| Duff_2017_Pilates_MS | Duff_2017_B | Control group | Control | 15 | 28.00 | 13.20 | 30.20 | 13.30 | FABS | Balance performance | Higher values better; no reversal | Yes |
| Eldemir_2024 | Eldemir_2024_A | Pilates-based telerehabilitation | Technology-delivered Pilates | 15 | 50.53 | 13.55 | 61.86 | 12.51 | LOS | Instrumented/laboratory postural control | Higher values better; no reversal | Yes |
| Eldemir_2024 | Eldemir_2024_B | Waitlist/no Pilates telerehabilitation | Control | 15 | 47.46 | 15.56 | 47.93 | 16.90 | LOS | Instrumented/laboratory postural control | Higher values better; no reversal | Yes |
| Hadas_2024 | Hadas_2024_A | Remote synchronous Zoom Pilates | Technology-delivered Pilates | 19 | 2.90 | 1.40 | 3.30 | 2.30 | Stork test | Balance performance | Higher values better; no reversal | Yes |
| Hadas_2024 | Hadas_2024_B | In-studio Pilates | Pilates | 19 | 2.30 | 1.20 | 3.50 | 1.70 | Stork test | Balance performance | Higher values better; no reversal | Yes |
| Junata_2021 | Junata_2021_A | Kinect-based rapid movement training | Technology-assisted exercise | 16 | 49.13 | 1.63 | 52.75 | 2.46 | BBS | Balance performance | Higher values better; no reversal | Yes |
| Junata_2021 | Junata_2021_B | Conventional balance training | Conventional exercise | 14 | 48.64 | 2.56 | 52.00 | 2.96 | BBS | Balance performance | Higher values better; no reversal | Yes |
| Kalron_2016 | Kalron_2016_A | Pilates exercise training | Pilates | 22 | 46.80 | 9.60 | 47.90 | 6.40 | BBS | Balance performance | Higher values better; no reversal | Yes |
| Kalron_2016 | Kalron_2016_B | Standardized physical therapy | Conventional exercise | 23 | 43.30 | 7.10 | 44.60 | 4.90 | BBS | Balance performance | Higher values better; no reversal | Yes |
| Lee_and_Yoo_2017 | LeeYoo_2017_A | AR-based Otago exercise | Technology-assisted exercise | 10 | −16.90 | 2.23 | −15.90 | 2.07 | EO CoP-x | Instrumented/laboratory postural control | Lower CoP displacement harmonized by sign reversal | Yes |
| Lee_and_Yoo_2017 | LeeYoo_2017_combined_conventional | Combined Yoga + Self-Otago exercise | Conventional exercise | 20 | −17.93 | 1.15 | −16.86 | 1.34 | EO CoP-x | Instrumented/laboratory postural control | Lower CoP displacement harmonized by sign reversal | Yes |
| Lee_2023_RingFit | Lee_2023_RingFit_A | Home-based Nintendo Ring Fit Adventure exergame | Technology-assisted exercise | 28 | 46.36 | 4.29 | 48.18 | 3.71 | BBS | Balance performance | Higher values better; no reversal | Yes |
| Lee_2023_RingFit | Lee_2023_RingFit_B | Online education/no exercise | Control | 29 | 44.62 | 4.81 | 44.86 | 4.70 | BBS | Balance performance | Higher values better; no reversal | Yes |
| Molhemi_2021_VR_MS | Molhemi_2021_A | Virtual reality-based balance training | Technology-assisted exercise | 19 | 46.60 | 3.90 | 52.40 | 2.10 | BBS | Balance performance | Higher values better; no reversal | Yes |
| Molhemi_2021_VR_MS | Molhemi_2021_B | Control balance/rehabilitation programme | Conventional exercise | 20 | 45.50 | 7.20 | 49.90 | 5.50 | BBS | Balance performance | Higher values better; no reversal | Yes |
| Sato_2015 | Sato_2015_A | Interactive video-game/Kinect exercise | Technology-assisted exercise | 28 | 29.36 | 4.40 | 33.88 | 3.94 | FRT | Balance performance | Higher values better; no reversal | Yes |
| Sato_2015 | Sato_2015_B | Control/usual activity | Control | 26 | 30.38 | 3.85 | 30.82 | 4.08 | FRT | Balance performance | Higher values better; no reversal | Yes |
| Yen_2011_VR_PD | Yen_2011_A | Virtual reality-augmented balance training | Technology-assisted exercise | 14 | 37.40 | 22.45 | 54.30 | 19.47 | SOT-6 single-task equilibrium score | Instrumented/laboratory postural control | Higher values better; no reversal | Yes |
| Yen_2011_VR_PD | Yen_2011_B | Conventional balance training | Conventional exercise | 14 | 40.20 | 22.45 | 55.00 | 19.47 | SOT-6 single-task equilibrium score | Instrumented/laboratory postural control | Higher values better; no reversal | Yes |
| Yen_2011_VR_PD | Yen_2011_C | No balance/no physical therapy control | Control | 14 | 44.50 | 22.45 | 42.10 | 19.47 | SOT-6 single-task equilibrium score | Instrumented/laboratory postural control | Higher values better; no reversal | Yes |

**Supplementary Table S7C. Additional analytic arms included only in the broader randomized-trial NMA**

| **Study/report ID** | **Arm ID** | **Arm name** | **Node** | **n** | **Baseline mean** | **Baseline SD** | **Post mean** | **Post SD** | **Outcome** | **Outcome domain** | **Direction harmonization** | **Use in broader RCT NMA** |
| --- | --- | --- | --- | --- | --- | --- | --- | --- | --- | --- | --- | --- |
| AibarAlmazan_2019 | AibarAlmazan_2019_A | Pilates method exercise programme | Pilates | 55 | −24.80 | 7.34 | −22.07 | 5.73 | FES-I | Fall-related proxy | Lower FES-I harmonized by sign reversal | Yes |
| AibarAlmazan_2019 | AibarAlmazan_2019_B | No formal intervention control | Control | 52 | −23.77 | 6.36 | −27.90 | 6.95 | FES-I | Fall-related proxy | Lower FES-I harmonized by sign reversal | Yes |
| Prasertsakul_2018_VR_Pilot | Prasertsakul_2018_A | Virtual reality-based balance training | Technology-assisted exercise | 4 | −313.22 | 309.38 | −90.34 | 64.55 | CoP sway area, eyes closed | Instrumented/laboratory postural control | Lower CoP area harmonized by sign reversal | Yes |
| Prasertsakul_2018_VR_Pilot | Prasertsakul_2018_B | Conventional balance training | Conventional exercise | 4 | −127.27 | 69.07 | −128.04 | 56.91 | CoP sway area, eyes closed | Instrumented/laboratory postural control | Lower CoP area harmonized by sign reversal | Yes |

**Supplementary Table S7D. Source arms and same-node arm combination in Lee and Yoo 2017**

| **Study/report ID** | **Source arm ID** | **Source arm name** | **Node** | **n** | **Baseline mean** | **Baseline SD** | **Post mean** | **Post SD** | **Outcome** | **Role in final analytic dataset** |
| --- | --- | --- | --- | --- | --- | --- | --- | --- | --- | --- |
| Lee_and_Yoo_2017 | LeeYoo_2017_A | AR-based Otago exercise | Technology-assisted exercise | 10 | −16.90 | 2.23 | −15.90 | 2.07 | EO CoP-x | Entered as technology-assisted exercise analytic arm |
| Lee_and_Yoo_2017 | LeeYoo_2017_Yoga_source | Yoga exercise | Conventional exercise | 10 | −18.05 | 1.38 | −16.66 | 1.30 | EO CoP-x | Source arm used only for same-node conventional-exercise combination |
| Lee_and_Yoo_2017 | LeeYoo_2017_SelfOtago_source | Self-Otago exercise | Conventional exercise | 10 | −17.80 | 0.93 | −17.06 | 1.42 | EO CoP-x | Source arm used only for same-node conventional-exercise combination |
| Lee_and_Yoo_2017 | LeeYoo_2017_combined_conventional | Combined Yoga + Self-Otago exercise | Conventional exercise | 20 | −17.93 | 1.15 | −16.86 | 1.34 | EO CoP-x | Final conventional-exercise analytic arm entered into contrast construction |

**Supplementary Table S7E. Baseline comparability audit based on selected outcomes**

| **Study/report ID** | **Baseline comparison assessed** | **Approximate maximum absolute standardized baseline difference** | **Baseline-comparability interpretation** | **Action in revised analysis** |
| --- | --- | --- | --- | --- |
| Abdelraouf_2020 | Technology-assisted exercise vs conventional exercise | 0.06 | No evident selected-outcome baseline imbalance | No baseline-imbalance exclusion sensitivity required |
| Bacha_2018_Kinect | Technology-assisted exercise vs conventional exercise | 0.36 | Possible selected-outcome baseline difference | Considered in transitivity and certainty judgments |
| Duff_2017_Pilates_MS | Pilates vs control | 0.06 | No evident selected-outcome baseline imbalance | No baseline-imbalance exclusion sensitivity required |
| Eldemir_2024 | Technology-delivered Pilates vs control | 0.21 | Possible selected-outcome baseline difference | Considered in transitivity and certainty judgments |
| Hadas_2024 | Technology-delivered Pilates vs Pilates | 0.46 | Possible selected-outcome baseline difference | Considered in transitivity and certainty judgments |
| Junata_2021 | Technology-assisted exercise vs conventional exercise | 0.23 | Possible selected-outcome baseline difference | Considered in transitivity and certainty judgments |
| Kalron_2016 | Pilates vs conventional exercise | 0.42 | Possible selected-outcome baseline difference | Considered in transitivity and certainty judgments |
| Lee_and_Yoo_2017 | Technology-assisted exercise vs combined conventional exercise | 0.65 | Potentially consequential selected-outcome baseline difference | Evaluated in additional baseline-imbalance exclusion sensitivity |
| Lee_2023_RingFit | Technology-assisted exercise vs control | 0.38 | Possible selected-outcome baseline difference | Considered in transitivity and certainty judgments |
| Molhemi_2021_VR_MS | Technology-assisted exercise vs conventional exercise | 0.19 | No evident selected-outcome baseline imbalance | No baseline-imbalance exclusion sensitivity required |
| Sato_2015 | Technology-assisted exercise vs control | 0.25 | Possible selected-outcome baseline difference | Considered in transitivity and certainty judgments |
| Yen_2011_VR_PD | Technology-assisted exercise, conventional exercise, and control pairwise comparisons | 0.32 | Possible selected-outcome baseline difference across direct arm comparisons | Considered in transitivity and certainty judgments |
| AibarAlmazan_2019 | Pilates vs control | 0.15 | No evident selected-outcome baseline imbalance; broader layer only | Considered only in broader randomized-trial interpretation |
| Prasertsakul_2018_VR_Pilot | Technology-assisted exercise vs conventional exercise | 0.83 | Potentially important selected-outcome baseline difference; broader/pilot layer only | Considered in broader-layer imprecision and certainty interpretation |

**Supplementary Table S7F. Nonrandomized controlled arm-level data retained outside the randomized-trial datasets**

| **Study/report ID** | **Revised status** | **Reason for exclusion from randomized-trial arm-level dataset** | Use in final review |
| --- | --- | --- | --- |
| Sapi_2019 | Nonrandomized controlled evidence outside randomized-trial NMA | First-come, first-served allocation was not compatible with randomized-trial primary inference. Therefore, its arm-level values were not entered into the primary or broader randomized-trial NMA dataset | Retained as contextual nonrandomized controlled evidence only; not used to determine primary randomized-trial estimates or certainty judgments |

**Supplementary Table S7G. Interpretation notes for baseline and arm-level extraction**

| **Issue** | **Revised handling** | **Interpretation consequence** |
| --- | --- | --- |
| Baseline values | Extracted for the selected outcome where available | Used to audit selected-outcome baseline comparability; not used as baseline-adjusted NMA |
| Post-intervention values | Used for Hedges’ g construction | Primary effect estimates were based on post-intervention arm-level values |
| Same-node arm combination | Lee and Yoo 2017 Yoga and Self-Otago arms were combined as one conventional-exercise analytic arm | Prevented double-counting same-node conventional-exercise arms within one randomized trial |
| Direction reversal | Lower-is-better outcomes were harmonized by sign reversal; SDs were unchanged | Positive Hedges’ g values consistently indicate better performance |
| Baseline imbalance | Lee and Yoo 2017 showed the largest potentially consequential selected-outcome baseline difference in the primary RCT network | An additional baseline-imbalance exclusion sensitivity analysis was performed |
| Nonrandomized controlled evidence | Sapi_2019 was retained outside the randomized-trial networks | Nonrandomized data were not entered into RCT NMA effect-size construction |
| Baseline standardized differences | Used descriptively to identify possible selected-outcome baseline imbalance | These values were not treated as definitive clinical thresholds or as adjusted treatment effects |

**Note.** BBS, Berg Balance Scale; CoP, center of pressure; EO CoP-x, eyes-open center-of-pressure displacement in the x-axis; FABS, Fullerton Advanced Balance Scale; FES-I, Falls Efficacy Scale-International; FRT, Functional Reach Test; LOS, limits of stability; NMA, network meta-analysis; RCT, randomized controlled trial; SD, standard deviation; SEBT, Star Excursion Balance Test; SMD, standardized mean difference; SOT, Sensory Organization Test. Values for lower-is-better outcomes are shown after sign reversal so that higher values consistently represent better performance. Source arms refer to original randomized study arms before same-node arm combination. Analytic arms refer to arms entered into network contrast construction after same-node combination. The primary randomized-trial NMA included 25 analytic arms and 491 participants. The broader randomized-trial NMA included 29 analytic arms and 606 participants. Baseline standardized differences were used only as descriptive audit measures and did not constitute baseline-adjusted network estimates.

**Supplementary Table S8. Intervention-node coding and same-node arm-combination decisions**

**Supplementary Table S8A. Revised intervention-node coding rules**

| **Node** | Coding rule used in the final review | **Examples of eligible arms** | **Main interpretation boundary** |
| --- | --- | --- | --- |
| Control | Comparator condition without structured exercise, Pilates-specific training, digital exercise, balance training, or active rehabilitation. Control conditions could include waitlist/no intervention, usual activity/usual care without structured exercise, education-only/minimal-contact control, or other non-exercise comparator conditions | Waitlist/no intervention; online education/no exercise; usual activity; no balance/no physical therapy; non-exercise comparator condition | Control was not treated as a clinically uniform condition. Control subtype was audited separately in Supplementary Table S10 and considered in sensitivity and certainty interpretation |
| Conventional exercise | Structured non-technology-assisted exercise, physical therapy, balance training, core-stability training, yoga/Otago-type exercise, or conventional rehabilitation without a Pilates-specific or digital-delivery component | Core stability exercise; conventional physical therapy; conventional balance training; standardized physical therapy; yoga; Self-Otago exercise | This node may include heterogeneous active exercise or rehabilitation formats. Exercise content, dose, and comparator intensity were treated as potential effect modifiers |
| Pilates | Pilates-based exercise delivered without digital, telecommunication, remote, or technology-mediated delivery | Face-to-face Pilates; in-studio Pilates; supervised non-digital Pilates training | This node represents Pilates-specific motor-control exercise, not generic core training unless the source study defined the intervention as Pilates-based |
| Technology-assisted exercise | Non-Pilates exercise materially augmented, supported, or delivered by digital technology, including virtual reality, augmented reality, exergaming, Kinect/motion-sensing systems, active video-game exercise, or digitally supported interactive balance/postural-control exercise | VR-based balance training; AR-based Otago exercise; Kinect-based training; Ring Fit Adventure; interactive video-game exercise | This node is a broad digitally augmented exercise category. It should not be interpreted as a platform-specific effect or as evidence that VR, AR, exergaming, and motion-sensing systems are interchangeable |
| Technology-delivered Pilates | Pilates-based exercise delivered through remote, digital, telecommunication, synchronous video, or technology-mediated delivery while retaining Pilates-specific exercise content | Pilates telerehabilitation; synchronous Zoom Pilates; digitally mediated Pilates programme | This node retains Pilates-specific motor-control content but differs from face-to-face Pilates in delivery mode, supervision, access requirements, and implementation context |

**Supplementary Table S8B. Primary randomized-trial arm-level node coding**

| **Study/report ID** | **Arm/source description** | **Revised node** | **Coding rationale** | **Final analytic use** |
| --- | --- | --- | --- | --- |
| Abdelraouf_2020 | Core stability exercise plus virtual reality | Technology-assisted exercise | Exercise programme augmented by virtual-reality technology | Entered primary RCT NMA |
| Abdelraouf_2020 | Core stability exercise | Conventional exercise | Structured non-digital exercise without Pilates-specific or technology-mediated delivery | Entered primary RCT NMA |
| Bacha_2018_Kinect | Kinect Adventures training | Technology-assisted exercise | Motion-sensing/Kinect-based digitally augmented exercise | Entered primary RCT NMA |
| Bacha_2018_Kinect | Conventional physical therapy | Conventional exercise | Structured non-digital physical therapy | Entered primary RCT NMA |
| Duff_2017_Pilates_MS | Pilates exercise programme | Pilates | Pilates-specific exercise delivered without digital/remote technology | Entered primary RCT NMA |
| Duff_2017_Pilates_MS | Control comparator | Control | Non-Pilates, non-technology, non-structured exercise comparator; control subtype audited separately | Entered primary RCT NMA |
| Eldemir_2024 | Pilates-based telerehabilitation | Technology-delivered Pilates | Pilates-specific exercise delivered through telerehabilitation/digital platform | Entered primary RCT NMA |
| Eldemir_2024 | Waitlist/no Pilates telerehabilitation | Control | No active Pilates telerehabilitation or structured exercise comparator | Entered primary RCT NMA |
| Hadas_2024 | Remote synchronous Zoom Pilates | Technology-delivered Pilates | Pilates-specific training delivered through synchronous remote/digital format | Entered primary RCT NMA |
| Hadas_2024 | In-studio Pilates | Pilates | Face-to-face/non-digital Pilates training | Entered primary RCT NMA |
| Junata_2021 | Kinect-based rapid movement training | Technology-assisted exercise | Kinect/motion-sensing digitally augmented exercise | Entered primary RCT NMA |
| Junata_2021 | Conventional balance training | Conventional exercise | Structured non-digital balance training | Entered primary RCT NMA |
| Kalron_2016 | Pilates exercise training | Pilates | Pilates-specific non-digital exercise | Entered primary RCT NMA |
| Kalron_2016 | Standardized physical therapy | Conventional exercise | Structured non-digital physical therapy | Entered primary RCT NMA |
| Lee_and_Yoo_2017 | AR-based Otago exercise | Technology-assisted exercise | Otago-type exercise augmented by augmented-reality technology | Entered primary RCT NMA |
| Lee_and_Yoo_2017 | Yoga exercise | Conventional exercise | Structured non-digital exercise comparator without Pilates-specific or digital-delivery component | Combined with Self-Otago source arm before contrast construction |
| Lee_and_Yoo_2017 | Self-Otago exercise | Conventional exercise | Structured non-digital/home exercise comparator without digital-delivery component | Combined with Yoga source arm before contrast construction |
| Lee_2023_RingFit | Home-based Nintendo Ring Fit Adventure exergame | Technology-assisted exercise | Commercial active video-game/exergaming intervention | Entered primary RCT NMA |
| Lee_2023_RingFit | Online education/no exercise | Control | Education-only/no structured exercise comparator | Entered primary RCT NMA |
| Molhemi_2021_VR_MS | Virtual reality-based balance training | Technology-assisted exercise | VR-based digitally augmented balance exercise | Entered primary RCT NMA |
| Molhemi_2021_VR_MS | Control balance/rehabilitation programme | Conventional exercise | Active non-digital balance/rehabilitation comparator rather than inactive control | Entered primary RCT NMA |
| Sato_2015 | Interactive video-game/Kinect exercise | Technology-assisted exercise | Interactive video-game/motion-sensing exercise | Entered primary RCT NMA |
| Sato_2015 | Control/usual activity | Control | Usual-activity/no structured digital or balance-training comparator | Entered primary RCT NMA |
| Yen_2011_VR_PD | Virtual reality-augmented balance training | Technology-assisted exercise | Balance training augmented by virtual-reality technology | Entered primary RCT NMA |
| Yen_2011_VR_PD | Conventional balance training | Conventional exercise | Structured non-digital balance training | Entered primary RCT NMA |
| Yen_2011_VR_PD | No balance/no physical therapy control | Control | No structured balance or physical-therapy comparator | Entered primary RCT NMA |

**Supplementary Table S8C. Broader randomized-trial arm-level node coding**

| **Study/report ID** | **Arm/source description** | **Revised node** | **Coding rationale** | **Final analytic use** |
| --- | --- | --- | --- | --- |
| AibarAlmazan_2019 | Pilates method exercise programme | Pilates | Pilates-specific exercise delivered without digital/remote technology | Broader randomized-trial NMA only |
| AibarAlmazan_2019 | No formal intervention control | Control | No formal structured exercise comparator | Broader randomized-trial NMA only |
| Prasertsakul_2018_VR_Pilot | Virtual reality-based balance training | Technology-assisted exercise | VR-based digitally augmented balance exercise | Broader randomized-trial NMA only |
| Prasertsakul_2018_VR_Pilot | Conventional balance training | Conventional exercise | Structured non-digital balance training | Broader randomized-trial NMA only |

**Supplementary Table S8D. Nonrandomized controlled evidence retained outside randomized-trial node analysis**

| **Study/report ID** | **Source arm/comparison previously considered** | **Potential node coding if considered descriptively** | **Revised status** | **Reason not entered into randomized-trial network** |
| --- | --- | --- | --- | --- |
| Sapi_2019 | Kinect-based exercise, conventional balance training, and no-intervention/control evidence as available in the source study | Technology-assisted exercise; conventional exercise; control | Nonrandomized controlled evidence outside randomized-trial NMA | Source verification identified first-come, first-served allocation; therefore the study was not compatible with randomized-trial primary inference and was not used in primary RCT estimates or primary RCT certainty judgments |

**Supplementary Table S8E. Same-node arm-combination decision for Lee and Yoo 2017**

| **Study/report ID** | **Source arm** | **Source node** | **Reason for same-node decision** | **Combination decision** | **Final analytic representation** |
| --- | --- | --- | --- | --- | --- |
| Lee_and_Yoo_2017 | AR-based Otago exercise | Technology-assisted exercise | AR technology materially augmented the exercise programme | Not combined with conventional arms | Entered as technology-assisted exercise analytic arm |
| Lee_and_Yoo_2017 | Yoga exercise | Conventional exercise | Structured non-digital exercise without Pilates-specific or technology-mediated delivery | Combined with Self-Otago because both represented the conventional-exercise node | Entered only through combined conventional-exercise analytic arm |
| Lee_and_Yoo_2017 | Self-Otago exercise | Conventional exercise | Structured non-digital/home exercise without technology-mediated delivery | Combined with Yoga because both represented the conventional-exercise node | Entered only through combined conventional-exercise analytic arm |
| Lee_and_Yoo_2017 | Combined Yoga + Self-Otago | Conventional exercise | Combined same-node comparator prevents double-counting two conventional-exercise arms from the same randomized trial | Final analytic arm | Entered as the conventional-exercise analytic arm |

Supplementary Table S8F. Interpretation boundaries for intervention-node coding

| **Issue** | **Revised handling** | **Interpretation consequence** |
| --- | --- | --- |
| Broad technology-assisted node | VR, AR, Kinect/motion-sensing, exergaming, active video-game, and related digitally augmented non-Pilates exercise formats were coded under technology-assisted exercise | Pooled estimate is not platform-specific and cannot guide selection of a specific technology platform |
| Technology-delivered Pilates separated from technology-assisted exercise | Pilates-based exercise delivered through digital/remote/technology-mediated platforms was coded separately | Preserves distinction between Pilates-specific content and generic digital exercise |
| Conventional exercise heterogeneity | Non-digital balance training, physical therapy, core-stability exercise, yoga, Otago-type exercise, and conventional rehabilitation could be coded as conventional exercise | Node is active and heterogeneous; comparator intensity was considered in transitivity and certainty judgments |
| Control-node heterogeneity | Inactive, usual-activity, education-only, and non-exercise comparator conditions were coded as control when no structured exercise or active rehabilitation was delivered | Control subtype was audited separately; control should not be interpreted as a uniform placebo/no-contact condition |
| Same-node multi-arm study | Same-node arms within a randomized trial were combined before contrast construction | Prevents double-counting and explains source-arm versus analytic-arm counts |
| Nonrandomized controlled evidence | Potential node labels could be described for context, but nonrandomized evidence did not enter randomized-trial NMA | Protects primary inference from nonrandom allocation |
| AI terminology | Node coding was based on intervention content and delivery, not on technology-readiness | Explicit AI or algorithmic features are described separately in Supplementary Table S9 and were not used to define efficacy nodes |

**Note.** AR, augmented reality; NMA, network meta-analysis; RCT, randomized controlled trial; VR, virtual reality. Node coding was based on exercise content, delivery mode, and comparator structure. Technology-assisted exercise was treated as a broad digitally augmented exercise category, whereas technology-delivered Pilates was coded separately because the Pilates-specific exercise content was retained. Same-node arms within a randomized trial were combined before contrast construction. Explicit AI or algorithmic features were not used to define efficacy nodes and are described separately in Supplementary Table S9.

**Supplementary Table S9. Technology modality, delivery characteristics, and explicitly reported algorithmic features**

**Supplementary Table S9A. Technology and algorithmic-feature coding framework**

| **Coding domain** | Definition used in the final review | **Coding categories** |
| --- | --- | --- |
| Technology modality | The main digital or technology-supported format used to deliver, augment, or support the exercise intervention | Virtual reality; augmented reality; Kinect/motion-sensing; exergaming/active video game; synchronous video or telecommunication delivery; telerehabilitation; non-digital face-to-face delivery |
| Delivery setting | Where or how the intervention was delivered | Clinic/laboratory; rehabilitation setting; home-based; remote/synchronous online; mixed or not clearly reported |
| Digital function | The primary function of technology within the intervention | Visual/auditory feedback; external cueing; motion tracking; interactive task practice; engagement/motivation support; remote supervision; digital delivery; none/non-digital |
| Supervision mode | Whether the intervention was supervised or delivered independently | Face-to-face supervised; remotely supervised; home-based independent; mixed; not clearly reported |
| Explicit AI or algorithmic component reported | Whether the source report explicitly described AI-driven decision support, machine-learning prediction, automated risk stratification, algorithm-guided adaptation, automated personalized progression, or AI-based monitoring | Yes; No explicit AI/algorithmic component reported; Unclear |
| Sensor or motion-tracking component | Whether the intervention used motion sensors, cameras, balance systems, or similar devices for interaction or feedback | Reported; not reported; unclear |
| Implementation reporting | Whether the study reported features relevant to clinical translation | Adherence; adverse events; usability/acceptability; equipment access; digital literacy; cost; therapist involvement; follow-up durability; equity considerations |
| Interpretation boundary | Restriction placed on interpretation of technology-related findings | Technology mapping was descriptive only and did not define efficacy nodes, treatment rankings, technology-readiness, or technology-efficacy claims |

**Supplementary Table S9B. Technology-related intervention arms in the primary randomized-trial network**

| **Study/report ID** | **Intervention arm** | **Network node** | **Technology modality** | **Delivery setting/supervision** | **Main digital function** | **Sensor/motion-tracking component** | **Explicit AI or algorithmic component reported?** | **Interpretation note** |
| --- | --- | --- | --- | --- | --- | --- | --- | --- |
| Abdelraouf_2020 | Core stability exercise plus VR | Technology-assisted exercise | Virtual reality-supported exercise | Supervised exercise setting | Visual feedback and digitally augmented motor-control/balance practice | Reported as VR-supported; specific sensor details depend on source report | No explicit AI/algorithmic component reported | Technology-assisted estimate should not be interpreted as platform-specific VR efficacy |
| Bacha_2018_Kinect | Kinect Adventures training | Technology-assisted exercise | Kinect/motion-sensing exergaming | Supervised or structured training setting | Motion-sensing interaction, feedback, and task engagement | Reported | No explicit AI/algorithmic component reported | Motion sensing was treated as digital augmentation, not AI |
| Eldemir_2024 | Pilates-based telerehabilitation | Technology-delivered Pilates | Telerehabilitation/digital Pilates delivery | Remote or technology-mediated delivery | Remote delivery and Pilates-specific exercise support | Not reported as AI-driven | No explicit AI/algorithmic component reported | Coded separately from technology-assisted exercise because Pilates content was retained |
| Hadas_2024 | Remote synchronous Zoom Pilates | Technology-delivered Pilates | Synchronous video/telecommunication Pilates | Remote synchronous supervision | Digital delivery and remote supervision | Not reported as AI-driven | No explicit AI/algorithmic component reported | Represents digitally delivered Pilates rather than generic technology-assisted exercise |
| Junata_2021 | Kinect-based rapid movement training | Technology-assisted exercise | Kinect/motion-sensing exercise | Structured rehabilitation/training setting | Motion-sensing task practice and feedback | Reported | No explicit AI/algorithmic component reported | Digital interaction may support task engagement but was not AI-personalized |
| Lee_and_Yoo_2017 | AR-based Otago exercise | Technology-assisted exercise | Augmented reality-supported exercise | Structured exercise setting | Augmented visual cueing and digitally supported exercise guidance | Reported as AR-supported; AI not reported | No explicit AI/algorithmic component reported | AR was treated as digital augmentation, not as AI-enabled rehabilitation |
| Lee_2023_RingFit | Nintendo Ring Fit Adventure exergame | Technology-assisted exercise | Commercial exergaming/active video game | Home-based digital exercise | Interactive game-based exercise and engagement support | Reported through commercial exergaming system | No explicit AI/algorithmic component reported | Commercial exergaming evidence cannot be generalized to all digital platforms |
| Molhemi_2021_VR_MS | Virtual reality-based balance training | Technology-assisted exercise | Virtual reality-based balance exercise | Structured rehabilitation/training setting | Visual feedback, task practice, and immersive or semi-immersive digital training | Reported as VR-supported | No explicit AI/algorithmic component reported | VR evidence remained part of a broad technology-assisted node |
| Sato_2015 | Interactive video-game/Kinect exercise | Technology-assisted exercise | Interactive video-game/motion-sensing exercise | Structured or home-compatible training context | Motion-sensing interaction, feedback, and engagement support | Reported | No explicit AI/algorithmic component reported | Interactive digital exercise was not interpreted as AI-driven adaptation |
| Yen_2011_VR_PD | Virtual reality-augmented balance training | Technology-assisted exercise | Virtual reality-augmented balance training | Supervised balance-training setting | Augmented feedback and task-specific balance practice | Reported as VR-augmented; AI not reported | No explicit AI/algorithmic component reported | Included as digitally augmented exercise; no AI efficacy inference |

**Supplementary Table S9C. Technology-related intervention arms included only in the broader randomized-trial network**

| **Study/report ID** | **Intervention arm** | **Network node** | **Technology modality** | **Delivery setting/supervision** | **Main digital function** | **Sensor/motion-tracking component** | **Explicit AI or algorithmic component reported?** | **Interpretation note** |
| --- | --- | --- | --- | --- | --- | --- | --- | --- |
| Prasertsakul_2018_VR_Pilot | Virtual reality-based balance training | Technology-assisted exercise | Virtual reality-based balance training | Pilot or laboratory-based training context | Visual feedback and instrumented/digital balance practice | Reported as VR-based | No explicit AI/algorithmic component reported | Broader-layer pilot evidence only; did not replace primary inference |

**Supplementary Table S9D. Non-digital or comparator arms relevant to technology interpretation**

| **Study/report ID** | **Comparator/intervention arm** | **Network node** | **Technology modality** | **Main role in the network** | **Interpretation note** |
| --- | --- | --- | --- | --- | --- |
| Abdelraouf_2020 | Core stability exercise | Conventional exercise | Non-digital exercise | Active comparator | Used to compare VR-augmented core stability against non-digital core stability |
| Bacha_2018_Kinect | Conventional physical therapy | Conventional exercise | Non-digital rehabilitation | Active comparator | Conventional comparator intensity considered in transitivity assessment |
| Duff_2017_Pilates_MS | Control group | Control | Non-digital/non-exercise comparator | Reference comparator | Control subtype audited separately |
| Eldemir_2024 | Waitlist/no Pilates telerehabilitation | Control | Non-digital/no active telerehabilitation | Reference comparator | Control subtype audited separately |
| Hadas_2024 | In-studio Pilates | Pilates | Non-digital face-to-face Pilates | Active Pilates comparator | Allowed separation of technology-delivered Pilates from face-to-face Pilates |
| Junata_2021 | Conventional balance training | Conventional exercise | Non-digital balance training | Active comparator | Used to compare digital/motion-sensing training with conventional balance training |
| Kalron_2016 | Pilates exercise training | Pilates | Non-digital Pilates | Active Pilates node | Pilates-specific comparator in active network |
| Kalron_2016 | Standardized physical therapy | Conventional exercise | Non-digital physical therapy | Active comparator | Comparator intensity considered in transitivity assessment |
| Lee_and_Yoo_2017 | Combined Yoga + Self-Otago exercise | Conventional exercise | Non-digital exercise | Active comparator | Same-node conventional arms combined before contrast construction |
| Lee_2023_RingFit | Online education/no exercise | Control | Education-only/non-exercise comparator | Reference comparator | Control subtype audited separately |
| Molhemi_2021_VR_MS | Control balance/rehabilitation programme | Conventional exercise | Non-digital balance/rehabilitation | Active comparator | Coded as conventional exercise rather than control |
| Sato_2015 | Control/usual activity | Control | Usual activity/no structured digital exercise | Reference comparator | Control subtype audited separately |
| Yen_2011_VR_PD | Conventional balance training | Conventional exercise | Non-digital balance training | Active comparator | Active comparator in three-arm RCT |
| Yen_2011_VR_PD | No balance/no physical therapy control | Control | Non-digital/no active physical-therapy comparator | Reference comparator | Control subtype audited separately |
| AibarAlmazan_2019 | Pilates method exercise programme | Pilates | Non-digital Pilates | Broader-layer active intervention | Broader RCT network only |
| AibarAlmazan_2019 | No formal intervention control | Control | Non-digital/no formal intervention | Broader-layer control | Broader RCT network only |
| Prasertsakul_2018_VR_Pilot | Conventional balance training | Conventional exercise | Non-digital balance training | Broader-layer active comparator | Broader RCT network only |

**Supplementary Table S9E. Node-level technology and implementation summary**

| **Network node** | **Technology/delivery content represented** | **Main digital function or non-digital comparator role** | **Explicit AI/algorithmic component reported?** | **Implementation limitations relevant to interpretation** |
| --- | --- | --- | --- | --- |
| Control | Waitlist/no intervention, usual activity, education-only/no exercise, no active balance/physical-therapy comparator, or other non-exercise reference conditions | Reference condition; not a uniform placebo/no-contact node | Not applicable | Control subtype varied across studies and was audited separately |
| Conventional exercise | Non-digital exercise, balance training, physical therapy, core stability, yoga, Otago-type exercise, or conventional rehabilitation | Active non-digital comparator | Not applicable | Content and dose were heterogeneous; comparator intensity may modify relative effects |
| Pilates | Non-digital Pilates-specific exercise | Pilates-specific motor-control exercise | No explicit AI/algorithmic component reported | Evidence base was sparse and population/context varied |
| Technology-assisted exercise | VR, AR, Kinect/motion-sensing, exergaming, active video-game, and related digitally augmented non-Pilates exercise formats | Digital feedback, cueing, motion tracking, interactive task practice, and engagement support | No explicit AI-driven decision support, machine-learning prediction, automated risk stratification, or algorithm-guided personalization was reported | Broad heterogeneous node; not platform-specific; cannot guide procurement of a specific device or software |
| Technology-delivered Pilates | Pilates-specific exercise delivered through telerehabilitation, synchronous video, or digital/remote platforms | Remote delivery, supervision, or technology-mediated Pilates instruction | No explicit AI/algorithmic component reported | Very sparse node; delivery mode differs from face-to-face Pilates and requires separate implementation evaluation |

**Supplementary Table S9F. Explicit AI or algorithmic-feature boundary**

| **Feature** | Classification in the final review | **Interpretation** |
| --- | --- | --- |
| Motion tracking, Kinect, VR, AR, or exergaming without reported predictive or adaptive algorithm | Digital technology or sensor-supported exercise | Not classified as AI-driven |
| Remote video delivery, telerehabilitation, or Zoom-based exercise | Digital delivery or remote supervision | Not classified as AI-driven |
| Commercial exergaming platform | Digitally supported interactive exercise | Not classified as AI-driven unless the source report explicitly described AI-based decision support or algorithmic adaptation |
| Sensor-based monitoring alone | Digital monitoring or motion tracking | Not classified as AI-driven unless linked to predictive modelling, automated risk stratification, or algorithm-guided adaptation |
| Predictive machine-learning model | Explicit AI/algorithmic component | Would be classified as explicitly AI/algorithmic if reported |
| Automated risk stratification | Explicit AI/algorithmic component | Would be classified as explicitly AI/algorithmic if reported |
| Algorithm-guided adaptive progression or personalization | Explicit AI/algorithmic component | Would be classified as explicitly AI/algorithmic if reported |
| AI-driven clinical decision support | Explicit AI/algorithmic component | Would be classified as explicitly AI/algorithmic if reported |

Note. AI, artificial intelligence; AR, augmented reality; NMA, network meta-analysis; RCT, randomized controlled trial; VR, virtual reality. Technology mapping was descriptive and did not define efficacy nodes, treatment ranking, certainty, or technology-efficacy claims. Interventions were classified as explicitly AI/algorithmic only when source reports described AI-driven decision support, machine-learning prediction, automated risk stratification, algorithm-guided adaptation, automated personalized progression, or AI-based monitoring. Standard digital sensors, motion tracking, VR, AR, Kinect, exergaming, remote delivery, or telerehabilitation alone were not treated as AI/algorithmic interventions.

**Supplementary Table S10. Control-node composition, comparator activity, and concurrent physical-activity/co-intervention audit**

**Supplementary Table S10A. Coding framework for control conditions, comparator activity, and non-intervention physical activity**

| **Coding domain** | Definition used in the final review | **Coding categories or interpretation** |
| --- | --- | --- |
| Control subtype | Classification of arms coded as the control node according to the source-described comparator condition | Waitlist/no intervention; no formal exercise/no physical therapy; usual activity/usual care; education-only/minimal-contact; active non-exercise co-intervention control; unclear or minimally described |
| Structured exercise or rehabilitation in comparator | Whether the comparator delivered structured exercise, physical therapy, balance training, Pilates, digital exercise, or active rehabilitation | Yes; No; unclear. Arms with structured exercise/rehabilitation were coded as conventional exercise rather than control |
| Non-exercise active comparator content | Whether the control condition included active non-exercise contact or treatment, such as massage, education, attention, or other non-exercise co-intervention | Yes; No; unclear |
| Habitual physical-activity instruction | Whether participants were instructed to maintain usual physical activity outside the assigned intervention | Reported; not reported; unclear |
| Additional exercise or rehabilitation restriction | Whether participants were asked to avoid additional structured exercise, rehabilitation, Pilates, balance training, or comparable co-interventions outside the assigned intervention | Reported; not reported; unclear |
| Outside-activity monitoring | Whether non-intervention physical activity was monitored using accelerometry, IPAQ, activity diary, exercise log, therapist record, or comparable tools | Reported; not reported; unclear |
| Co-intervention or contamination concern | Whether the source-described comparator condition, outside-activity reporting, or differential contact could affect interpretation of the assigned intervention effect | Low concern; possible concern; sensitivity-relevant concern; unclear |
| Sensitivity-analysis role | Whether the control/comparator issue was assessed in an additional sensitivity analysis | Retained; excluded in sensitivity analysis; broader-layer only; not applicable |
| Interpretation principle | How these variables were used in the final review | Control subtype and concurrent-activity reporting were used to inform transitivity, indirectness, applicability, and certainty judgments; they were not used to create new efficacy nodes |

**Supplementary Table S10B. Control-node composition in the primary randomized-trial network**

| **Study/report ID** | **Control arm/source description** | **Revised control subtype** | **Structured exercise or rehabilitation in control?** | **Non-exercise active comparator content?** | **Final node coding** | **Sensitivity-analysis handling** | **Interpretation note** |
| --- | --- | --- | --- | --- | --- | --- | --- |
| Duff_2017_Pilates_MS | Massage-only or active non-exercise comparator/control condition | Active non-exercise co-intervention control | No structured exercise coded as control | Yes | Control | Excluded in non-exercise/minimal-intervention control sensitivity | This comparator was not a pure inactive or no-contact control and was treated as a control-node heterogeneity concern |
| Eldemir_2024 | Waitlist/no Pilates telerehabilitation | Waitlist/no active Pilates telerehabilitation | No | No or unclear | Control | Retained | Minimally active reference condition relative to Pilates telerehabilitation |
| Lee_2023_RingFit | Online education/no exercise | Education-only/minimal-contact control | No | Education/contact present | Control | Retained | Education/contact may differ from waitlist/no-intervention control and was considered in control-node heterogeneity |
| Sato_2015 | Usual activity control | Usual activity/usual-care control | No structured exercise reported | No or unclear | Control | Retained | Usual activity differs from pure waitlist/no-intervention control |
| Yen_2011_VR_PD | No balance/no physical therapy control | No formal balance training/no physical therapy | No formal balance training or physical therapy | No or unclear | Control | Retained | Not equivalent to complete inactivity; retained as a non-exercise/minimal-intervention reference condition |

**Supplementary Table S10C. Control-node composition in the broader randomized-trial network only**

| **Study/report ID** | **Control arm/source description** | **Revised control subtype** | **Structured exercise or rehabilitation in control?** | **Non-exercise active comparator content?** | **Final node coding** | **Sensitivity-analysis handling** | **Interpretation note** |
| --- | --- | --- | --- | --- | --- | --- | --- |
| AibarAlmazan_2019 | No formal intervention control | No formal intervention | No | No or unclear | Control | Broader randomized-trial network only | Broader-layer fall-related proxy evidence only |
| Sapi_2019 | No-intervention/control evidence in source report | Nonrandomized control evidence outside RCT networks | Not applicable to randomized-trial networks | Not applicable | Not entered into RCT networks | Not applicable | Nonrandom allocation; excluded from the randomized-trial primary and broader NMA |

**Supplementary Table S10D. Active comparator arms not coded as control**

| **Study/report ID** | **Active comparator/source description** | **Revised node** | **Reason not coded as control** | **Transitivity or interpretation note** |
| --- | --- | --- | --- | --- |
| Abdelraouf_2020 | Core stability exercise | Conventional exercise | Structured active exercise comparator | Technology-assisted estimate reflects VR-augmented exercise relative to active non-digital exercise, not relative to inactive control |
| Bacha_2018_Kinect | Conventional physical therapy | Conventional exercise | Structured physical therapy comparator | Active comparator intensity may attenuate differences versus technology-assisted exercise |
| Junata_2021 | Conventional balance training | Conventional exercise | Structured balance-training comparator | Active comparator should not be interpreted as control |
| Kalron_2016 | Standardized physical therapy | Conventional exercise | Structured physical therapy comparator | Contributes direct Pilates versus conventional exercise evidence |
| Lee_and_Yoo_2017 | Combined Yoga + Self-Otago exercise | Conventional exercise | Structured non-digital exercise comparator | Same-node conventional arms combined before contrast construction |
| Molhemi_2021_VR_MS | Control balance/rehabilitation programme | Conventional exercise | Structured balance/rehabilitation content was present | Coded as conventional exercise rather than control |
| Yen_2011_VR_PD | Conventional balance training | Conventional exercise | Structured balance-training comparator | In the three-arm trial, conventional exercise and control were coded separately |
| Hadas_2024 | In-studio Pilates | Pilates | Pilates-specific active comparator | Allows comparison of technology-delivered Pilates versus face-to-face Pilates |
| Prasertsakul_2018_VR_Pilot | Conventional balance training | Conventional exercise | Structured balance-training comparator | Broader randomized-trial NMA only |

**Supplementary Table S10E. Concurrent physical activity and co-intervention reporting audit**

| **Study/report ID** | **Habitual physical-activity instruction reported?** | **Additional structured exercise or rehabilitation restricted?** | **Outside activity monitored?** | **Monitoring instrument or source** | **Co-intervention/contact concern** | **How this information was used** |
| --- | --- | --- | --- | --- | --- | --- |
| Abdelraouf_2020 | Not clearly reported in extracted source information | Not clearly reported | Not clearly reported | NR | Possible residual concern | Considered in transitivity and certainty interpretation |
| Bacha_2018_Kinect | Not clearly reported in extracted source information | Not clearly reported | Not clearly reported | NR | Possible residual concern | Considered in transitivity and certainty interpretation |
| Duff_2017_Pilates_MS | Not clearly reported in extracted source information | Not clearly reported | Not clearly reported | NR | Sensitivity-relevant because the control condition included active non-exercise comparator content | Evaluated through exclusion of the active non-exercise control study |
| Eldemir_2024 | Not clearly reported in extracted source information | Not clearly reported | Not clearly reported | NR | Possible residual concern | Considered in certainty and implementation interpretation |
| Hadas_2024 | Not clearly reported in extracted source information | Not clearly reported | Not clearly reported | NR | Possible residual concern; remote versus in-studio delivery may differ in supervision and adherence context | Considered in transitivity and implementation interpretation |
| Junata_2021 | Not clearly reported in extracted source information | Not clearly reported | Not clearly reported | NR | Possible rehabilitation-context co-intervention concern | Considered in transitivity and certainty interpretation |
| Kalron_2016 | Not clearly reported in extracted source information | Not clearly reported | Not clearly reported | NR | Possible residual concern | Considered in transitivity and certainty interpretation |
| Lee_and_Yoo_2017 | Not clearly reported in extracted source information | Not clearly reported | Not clearly reported | NR | Possible residual concern; also selected-outcome baseline-imbalance concern | Considered in baseline, transitivity, and certainty interpretation |
| Lee_2023_RingFit | Not clearly reported in extracted source information | Not clearly reported | Not clearly reported | NR | Possible home-based adherence and outside-activity concern | Considered in implementation and certainty interpretation |
| Molhemi_2021_VR_MS | Not clearly reported in extracted source information | Not clearly reported | Not clearly reported | NR | Possible rehabilitation-context co-intervention concern | Considered in transitivity and certainty interpretation |
| Sato_2015 | Usual-activity control described; detailed outside-activity monitoring not clearly reported | Not clearly reported | Not clearly reported | NR | Possible habitual-activity concern | Considered in control-node and transitivity interpretation |
| Yen_2011_VR_PD | No balance/no physical therapy control described; broader activity outside protocol not clearly reported | Balance/physical-therapy restriction implied by control description; other outside activity unclear | Not clearly reported | NR | Possible residual concern | Considered in control-node and certainty interpretation |
| AibarAlmazan_2019 | No formal intervention control described; detailed outside-activity monitoring not clearly reported | Not clearly reported | Not clearly reported | NR | Broader-layer concern | Considered only in broader randomized-trial interpretation |
| Prasertsakul_2018_VR_Pilot | Not clearly reported in extracted source information | Not clearly reported | Not clearly reported | NR | Pilot/broader-layer concern | Considered only in broader randomized-trial interpretation |

Supplementary Table S10F. Control-node sensitivity-analysis definition

| **Sensitivity analysis** | **Inclusion/exclusion rule** | **Rationale** | **Interpretation boundary** |
| --- | --- | --- | --- |
| Non-exercise/minimal-intervention control sensitivity | Excluded active non-exercise co-intervention control evidence while retaining no formal intervention, waitlist/no active intervention, usual activity/usual care without structured exercise, education-only/no exercise, and no formal balance/physical-therapy control conditions | To examine whether estimates versus control were sensitive to inclusion of active non-exercise comparator content within the control node | This analysis was not interpreted as a pure placebo/no-contact or strictly inactive-control analysis; it assessed structural sensitivity of the control node |
| Active non-exercise control exclusion | The active non-exercise comparator/control evidence from Duff_2017_Pilates_MS was excluded | To address the possibility that active non-exercise contact or treatment differed meaningfully from minimally active control conditions | Results were interpreted cautiously because direct-evidence structure and indirect evidence contributions changed |
| Usual activity/usual care retained | Usual activity/no formal structured exercise control conditions were retained | These conditions remained within the non-exercise/minimal-intervention reference category | Usual activity is not equivalent to complete inactivity and was discussed as a limitation |
| Education-only/minimal-contact retained | Education-only/no exercise comparator conditions were retained | These comparators did not deliver structured exercise or active rehabilitation | Education/contact may affect expectations and attention and was considered in certainty interpretation |
| No formal balance/no physical therapy retained | Control conditions explicitly lacking formal balance training or physical therapy were retained | These conditions did not include structured balance/physical-therapy intervention | They should not be interpreted as complete physical inactivity |

Note. IPAQ, International Physical Activity Questionnaire; NMA, network meta-analysis; NR, not reported in the extracted source information; RCT, randomized controlled trial. Control conditions were not assumed to be clinically identical. The final review subclassified control arms and evaluated an additional non-exercise/minimal-intervention control sensitivity analysis. Concurrent physical activity and co-intervention information were extracted where reported; absence of reporting was coded as NR rather than as evidence that no additional activity occurred. These variables were incorporated into structured transitivity, indirectness, applicability, and certainty judgments.

**Supplementary Table S11. Structured transitivity assessment across direct comparisons**

**Supplementary Table S11A. Transitivity framework and candidate effect modifiers**

| **Candidate effect modifier** | **Why it may affect relative treatment effects** | How it was assessed in the final review | **Use in interpretation** |
| --- | --- | --- | --- |
| Population/pathology | Balance impairment may differ between age-related decline, neurological disease, musculoskeletal pain, athletic populations, and healthy adults | Extracted at study level and compared across direct comparisons | Used to judge indirectness and transitivity fragility |
| Age or life-stage context | Exercise responsiveness, fall-risk profile, digital familiarity, and baseline balance capacity may differ by age group | Extracted as source-reported age group/population context | Considered in comparison-level transitivity assessment |
| Mechanism of balance impairment | Neurological, musculoskeletal, age-related, and healthy-population mechanisms may not be exchangeable | Categorized as neurological, age-related, musculoskeletal/athletic, healthy/sedentary, or mixed | Used to identify clinically non-exchangeable comparison links |
| Baseline balance/fall-risk status | Post-intervention SMDs may partly reflect baseline imbalance or different baseline severity | Baseline values and standardized baseline differences were audited in Supplementary Table S7 | Used in transitivity and certainty interpretation |
| Intervention modality | Pilates, conventional exercise, digital feedback, VR/AR, motion sensing, and remote Pilates may operate through different mechanisms | Node coding and technology mapping were audited in Supplementary Tables S8–S9 | Used to avoid platform-specific inference |
| Intervention dose and supervision | Frequency, duration, intensity, therapist supervision, and home-based delivery may modify effects | Extracted where reported and summarized qualitatively | Treated as a potential effect modifier; not modelled because of network sparsity |
| Comparator type and intensity | Active conventional comparators differ from waitlist/usual activity/education-only controls | Control and comparator conditions were audited in Supplementary Table S10 | Incorporated into transitivity and sensitivity interpretation |
| Control subtype | Waitlist, usual activity, education-only, no physical therapy, and active non-exercise control are not equivalent | Subclassified and assessed in control-node audit and sensitivity analysis | Used to interpret estimates versus control |
| Outcome domain | Clinical balance tests, instrumented postural-control measures, and fall-related proxy outcomes may not represent identical constructs | Outcome hierarchy and direction harmonization were audited in Supplementary Table S12 | Evaluated through outcome-domain sensitivity analysis |
| Timing of assessment | Immediate post-intervention and longer follow-up effects may differ | Immediate post-intervention assessment was prioritized | Reduced timing heterogeneity in primary effect construction |
| Concurrent physical activity/co-intervention | Additional unmonitored activity or co-interventions may alter observed effects | Extracted where reported and coded as NR when not reported | Considered under transitivity, RoB2 Domain 2, and certainty |
| Evidence sparsity | Sparse direct comparisons limit ability to verify exchangeability | Number of studies and direct comparisons assessed for each contrast | Led to cautious transitivity judgments |

**Supplementary Table S11B. Study-level effect modifiers in the primary randomized-trial network**

| **Study/report ID** | **Population/pathology** | **Broad mechanism/context** | **Intervention and comparator structure** | **Outcome domain** | **Control/comparator subtype** | **Key transitivity concern** |
| --- | --- | --- | --- | --- | --- | --- |
| Abdelraouf_2020 | Athletes with nonspecific low back pain | Musculoskeletal/athletic population; not an older-adult or neurological rehabilitation sample | Technology-assisted exercise vs conventional exercise | Balance performance | Active conventional exercise comparator | Population differs substantially from older-adult and neurological rehabilitation studies; retained but flagged in population-sensitivity analysis |
| Bacha_2018_Kinect | Community-dwelling older adults | Age-related balance context | Technology-assisted exercise vs conventional exercise | Balance performance | Active conventional physical therapy comparator | More clinically aligned with older-adult balance rehabilitation, but active comparator intensity may influence relative effect |
| Duff_2017_Pilates_MS | People with multiple sclerosis | Neurological rehabilitation | Pilates vs control | Balance performance | Active non-exercise/massage-type control concern | Control condition differs from inactive/usual-care controls; direct Pilates-control evidence sparse |
| Eldemir_2024 | People with multiple sclerosis | Neurological rehabilitation | Technology-delivered Pilates vs control | Instrumented/laboratory postural control | Waitlist/no active Pilates telerehabilitation | Sparse direct evidence; outcome and delivery mode differ from face-to-face exercise trials |
| Hadas_2024 | Healthy sedentary women | Healthy/sedentary adult exercise context | Technology-delivered Pilates vs Pilates | Balance performance | Active face-to-face Pilates comparator | Population and delivery setting differ from neurological and older-adult rehabilitation samples |
| Junata_2021 | Chronic stroke survivors | Neurological rehabilitation | Technology-assisted exercise vs conventional exercise | Balance performance | Active conventional balance training | Neurological post-stroke context differs from MS, PD, older adults, and athletic samples |
| Kalron_2016 | People with multiple sclerosis | Neurological rehabilitation | Pilates vs conventional exercise | Balance performance | Active standardized physical therapy comparator | One direct Pilates-conventional link; clinical context limited to MS |
| Lee_and_Yoo_2017 | Elderly women | Age-related fall-prevention context | Technology-assisted exercise vs combined conventional exercise | Instrumented/laboratory postural control | Active non-digital exercise comparator | Same-node conventional arms combined; selected-outcome baseline imbalance flagged; instrumented outcome differs from clinical balance tests |
| Lee_2023_RingFit | Community-dwelling older adults | Age-related home-based digital exercise context | Technology-assisted exercise vs control | Balance performance | Education-only/no exercise control | Home-based exergame and education-only comparator may differ from supervised rehabilitation settings |
| Molhemi_2021_VR_MS | People with multiple sclerosis | Neurological rehabilitation | Technology-assisted exercise vs conventional exercise | Balance performance | Active balance/rehabilitation comparator | MS rehabilitation context; active comparator differs from inactive controls |
| Sato_2015 | Healthy elderly adults | Age-related exercise context | Technology-assisted exercise vs control | Balance performance | Usual activity/no structured exercise control | Usual activity control not equivalent to waitlist/no intervention |
| Yen_2011_VR_PD | People with Parkinson disease | Neurological rehabilitation | Technology-assisted exercise, conventional exercise, and control | Instrumented/laboratory postural control | No formal balance/no physical therapy control | Three-arm neurological trial; outcome and disease mechanism differ from MS, stroke, and older-adult trials |

**Supplementary Table S11C. Study-level effect modifiers in broader-only randomized evidence**

| **Study/report ID** | **Population/pathology** | **Broad mechanism/context** | **Intervention and comparator structure** | **Outcome domain** | **Control/comparator subtype** | **Key transitivity concern** |
| --- | --- | --- | --- | --- | --- | --- |
| AibarAlmazan_2019 | Community-dwelling elderly women | Age-related fall-risk context | Pilates vs control | Fall-related proxy | No formal intervention control | Broader-layer evidence only; FES-I is a fall-related proxy rather than a direct balance/postural-control endpoint |
| Prasertsakul_2018_VR_Pilot | Healthy adults | Healthy adult motor-learning/postural-control context | Technology-assisted exercise vs conventional exercise | Instrumented/laboratory postural control | Active conventional balance training | Pilot/broader-layer evidence; very small sample and healthy adult population limit exchangeability with older-adult and neurological rehabilitation trials |

**Supplementary Table S11D. Direct-comparison-level transitivity assessment in the primary randomized-trial network**

| **Direct comparison** | **Direct evidence contributing to comparison** | **Main populations represented** | **Main comparator/control structure** | **Outcome domain(s)** | **Effect-modifier distribution** | **Overall transitivity concern** |
| --- | --- | --- | --- | --- | --- | --- |
| Technology-assisted exercise vs conventional exercise | Abdelraouf_2020; Bacha_2018_Kinect; Junata_2021; Lee_and_Yoo_2017; Molhemi_2021_VR_MS; Yen_2011_VR_PD | Athletic low-back-pain participants; community-dwelling older adults; chronic stroke survivors; elderly women; people with MS; people with PD | Active conventional exercise/physical therapy/balance training comparators | Balance performance and instrumented/laboratory postural control | Highly heterogeneous populations, pathology mechanisms, technology formats, comparator intensity, and outcome domains | Major concern |
| Technology-assisted exercise vs control | Lee_2023_RingFit; Sato_2015; Yen_2011_VR_PD | Community-dwelling older adults; healthy elderly adults; people with PD | Education-only/no exercise; usual activity; no balance/no physical therapy control | Balance performance and instrumented/laboratory postural control | Control subtype, disease context, setting, and outcome domain differed across studies | Major concern |
| Pilates vs control | Duff_2017_Pilates_MS | People with MS | Active non-exercise comparator/control condition | Balance performance | Single direct RCT link; control condition not equivalent to inactive/usual-care control; local direct–indirect disagreement detected in primary network | Major concern |
| Technology-delivered Pilates vs control | Eldemir_2024 | People with MS | Waitlist/no active Pilates telerehabilitation | Instrumented/laboratory postural control | Single direct RCT link; remote Pilates delivery and neurological context only | Major concern |
| Technology-delivered Pilates vs Pilates | Hadas_2024 | Healthy sedentary women | Active face-to-face Pilates comparator | Balance performance | Single direct RCT link; healthy/sedentary population and delivery comparison may not generalize to older-adult or neurological rehabilitation settings | Major concern |
| Pilates vs conventional exercise | Kalron_2016 | People with MS | Active standardized physical therapy comparator | Balance performance | Single direct RCT link in MS only | Major concern |
| Conventional exercise vs control | Yen_2011_VR_PD | People with PD | No balance/no physical therapy control | Instrumented/laboratory postural control | Single direct RCT link; neurological disease-specific context | Major concern |
| Technology-assisted exercise vs Pilates | No direct primary RCT evidence | Indirect only | Indirect only | Indirect only | Entirely indirect comparison; transitivity depends on multiple heterogeneous indirect paths | Major concern |
| Technology-assisted exercise vs technology-delivered Pilates | No direct primary RCT evidence | Indirect only | Indirect only | Indirect only | Entirely indirect comparison; depends on broad technology-assisted and sparse technology-delivered Pilates evidence | Major concern |
| Conventional exercise vs Pilates-related digital delivery nodes | Mostly indirect or single-link pathways | Mixed | Mixed | Mixed | Sparse and structurally dependent on heterogeneous direct comparisons | Major concern |

**Supplementary Table S11E. Additional transitivity considerations in the broader randomized-trial network**

| **Added broader evidence** | **Comparison affected** | **Additional effect modifier introduced** | **Interpretation** |
| --- | --- | --- | --- |
| AibarAlmazan_2019 | Pilates vs control | Fall-related proxy outcome and community-dwelling elderly women | Supports broader contextual direction but introduces outcome-domain heterogeneity; did not replace primary balance/postural-control inference |
| Prasertsakul_2018_VR_Pilot | Technology-assisted exercise vs conventional exercise | Healthy adult pilot evidence, very small sample, instrumented postural-control outcome | Supports broader sensitivity mapping but increases sparsity and population/outcome heterogeneity |
| Broader network as a whole | All active-versus-control and active-versus-active comparisons | Broader-layer evidence increases network size but also adds outcome-domain and population heterogeneity | Used only as sensitivity evidence layer |

**Supplementary Table S11F. Sensitivity analyses used to examine transitivity-related concerns**

| **Concern** | **Sensitivity/audit used** | **What the analysis addressed** | **Interpretation boundary** |
| --- | --- | --- | --- |
| Athletic low-back-pain population may not be exchangeable with older-adult or neurological rehabilitation populations | Exclusion of athlete/low-back-pain study; clinical/older-rehabilitation population restriction | Tested whether obvious population outlier dominated point estimates | Did not eliminate clinical heterogeneity across MS, stroke, PD, older adults, and healthy/sedentary samples |
| Baseline imbalance may influence post-intervention SMDs | Baseline audit and exclusion of Lee_and_Yoo_2017 | Tested sensitivity to the study with potentially consequential selected-outcome baseline difference | Did not create baseline-adjusted NMA |
| Control-node heterogeneity may affect estimates versus control | Non-exercise/minimal-intervention control sensitivity | Tested influence of active non-exercise comparator/control evidence | Did not create a pure placebo/no-contact or strictly inactive-control network |
| Outcome-domain heterogeneity may affect relative estimates | Clinical/field-based balance-performance outcome restriction | Tested sensitivity to excluding some instrumented/laboratory or broader outcome domains | Network remained sparse; results remained exploratory |
| Technology-assisted node combines different digital platforms | Technology modality audit | Clarified that VR, AR, Kinect, exergaming, and active video-game interventions were not assumed to be interchangeable at platform level | No platform-specific NMA was performed because of sparse data |
| Sparse direct evidence may obscure inconsistency | Direct–indirect and node-splitting diagnostics | Identified potential Pilates-control direct–indirect disagreement in primary network | Absence of global signal was not interpreted as proof of consistency |

**Supplementary Table S11G. Final transitivity judgment for the revised randomized-trial networks**

| **Assessment level** | **Judgment** | **Rationale** |
| --- | --- | --- |
| Broad construct-level transitivity | Partially plausible | All synthesized interventions targeted balance-, postural-control-, or fall-related performance through active exercise, motor-control training, digital augmentation, or comparator conditions relevant to exercise rehabilitation |
| Clinical exchangeability across all direct comparisons | Clinically fragile | Direct comparisons linked heterogeneous populations, including athletes with low-back pain, community-dwelling older adults, healthy sedentary adults, stroke survivors, people with MS, and people with PD |
| Intervention-node exchangeability | Limited | Technology-assisted exercise combined VR, AR, Kinect/motion-sensing, exergaming, and active video-game formats; conventional exercise also included multiple active non-digital formats |
| Comparator/control exchangeability | Limited | Control conditions varied across active non-exercise control, waitlist/no intervention, education-only/no exercise, usual activity, and no formal balance/physical therapy |
| Outcome-domain exchangeability | Limited | Primary network included balance-performance tests and instrumented/laboratory postural-control outcomes; broader network also included fall-related proxy evidence |
| Statistical ability to verify transitivity/incoherence | Limited | Network was small and sparse; several comparisons were single-study or indirect-only |
| Overall judgment | Transitivity was considered plausible only at a broad balance-oriented exercise-category level and clinically fragile | Network estimates should be interpreted as exploratory, not as evidence of a definitive treatment hierarchy |

**Supplementary Table S11H. How transitivity concerns were incorporated into certainty assessment**

| Concern type | Primary certainty domain | Double-counting rule |
| --- | --- | --- |
| Population/pathology, intervention-node breadth, technology modality, control subtype, outcome domain, and transitivity/exchangeability concerns | Indirectness | Grouped under indirectness when clinically linked; not also downgraded as heterogeneity or applicability. |
| Small sample size, sparse direct evidence, indirect-only evidence, and wide or null-crossing confidence intervals | Imprecision | Assessed together under imprecision; sparse evidence was not counted again as indirectness unless the concern was clinical exchangeability. |
| Direct-indirect disagreement or node-splitting signal | Incoherence | Applied only when a comparison-specific inconsistency signal was estimable or clinically relevant. |
| Statistical heterogeneity signal beyond clinical indirectness | Heterogeneity | Used only for residual statistical heterogeneity not already captured under indirectness. |
| Risk of bias from included randomized trials | Within-study bias | RoB2 concerns were counted under within-study bias and not repeated in indirectness. |
| Unavailable compatible numerical data and non-contributing eligible reports | Reporting/data availability | Considered separately from imprecision to avoid double counting missingness and wide intervals. |

**Note.** MS, multiple sclerosis; NMA, network meta-analysis; PD, Parkinson disease; RCT, randomized controlled trial; SMD, standardized mean difference. Transitivity was assessed structurally rather than assumed from statistical heterogeneity tests. Concern levels reflect the plausibility that effect modifiers were sufficiently similar across direct comparisons to support indirect comparisons. Because the network was sparse and clinically heterogeneous, no comparison was judged as definitively satisfying transitivity. These judgments were used to support conservative interpretation and comparison-level GRADE/CINeMA-informed certainty assessment.

**Supplementary Table S12. Outcome selection, hierarchy, and direction harmonization**

**Supplementary Table S12A. Revised outcome hierarchy used for the randomized-trial networks**

| **Priority level** | **Outcome category** | **Examples** | **Direction rule** | **Use in revised analysis** |
| --- | --- | --- | --- | --- |
| Priority 1 | Clinical or field-based balance-performance outcomes | BBS, Mini-BESTest, FABS, FRT, SEBT, Stork test | Higher values generally indicate better balance performance | Preferred outcome type for the primary randomized-trial NMA when available; used for the clinical/field-based balance-performance sensitivity analysis |
| Priority 2 | Instrumented or laboratory-based postural-control outcomes | LOS, SOT equilibrium score, CoP displacement, CoP sway area, postural-sway measures | Direction harmonized before effect-size construction; lower-is-better outcomes were sign-reversed | Used when a clinical/field-based balance-performance outcome was unavailable or when the prioritized construct was postural control |
| Priority 3 | Fall-related proxy outcomes | FES-I or comparable fear-of-falling/falls-efficacy proxy outcomes | Lower values indicate better fall-related status; values were sign-reversed before effect-size construction | Used only in the broader randomized-trial network; not used to replace the primary balance/postural-control analysis |
| Not eligible for NMA effect-size construction | Outcomes unrelated to balance, postural control, or fall-related performance, or outcomes without compatible quantitative input | Pain-only, strength-only, quality-of-life-only, adherence-only, satisfaction-only, or non-compatible outcomes | Not applicable | Not entered into NMA effect-size construction |

**Supplementary Table S12B. Outcome selection and direction harmonization in the primary randomized-trial NMA**

| **Study/report ID** | **Outcome selected for revised NMA** | **Outcome category** | **Raw direction in source measure** | **Harmonization applied** | **Use in primary RCT NMA** | **Use in clinical/field-based balance-performance sensitivity** |
| --- | --- | --- | --- | --- | --- | --- |
| Abdelraouf_2020 | SEBT posteromedial reach | Clinical/field-based balance performance | Higher values better | No reversal | Yes | Yes |
| Bacha_2018_Kinect | Mini-BESTest | Clinical/field-based balance performance | Higher values better | No reversal | Yes | Yes |
| Duff_2017_Pilates_MS | FABS | Clinical/field-based balance performance | Higher values better | No reversal | Yes | Yes |
| Eldemir_2024 | LOS | Instrumented/laboratory postural control | Higher values better | No reversal | Yes | No; excluded from clinical/field-based balance-performance sensitivity |
| Hadas_2024 | Stork test | Clinical/field-based balance performance | Higher values better | No reversal | Yes | Yes |
| Junata_2021 | BBS | Clinical/field-based balance performance | Higher values better | No reversal | Yes | Yes |
| Kalron_2016 | BBS | Clinical/field-based balance performance | Higher values better | No reversal | Yes | Yes |
| Lee_and_Yoo_2017 | EO CoP-x | Instrumented/laboratory postural control | Lower CoP displacement better | Sign reversal applied | Yes | No; excluded from clinical/field-based balance-performance sensitivity |
| Lee_2023_RingFit | BBS | Clinical/field-based balance performance | Higher values better | No reversal | Yes | Yes |
| Molhemi_2021_VR_MS | BBS | Clinical/field-based balance performance | Higher values better | No reversal | Yes | Yes |
| Sato_2015 | FRT | Clinical/field-based balance performance | Higher values better | No reversal | Yes | Yes |
| Yen_2011_VR_PD | SOT-6 single-task equilibrium score | Instrumented/laboratory postural control | Higher values better | No reversal | Yes | No; excluded from clinical/field-based balance-performance sensitivity |

**Supplementary Table S12C. Outcome selection and direction harmonization in broader-only randomized evidence**

| **Study/report ID** | **Outcome selected for revised NMA** | **Outcome category** | **Raw direction in source measure** | **Harmonization applied** | **Use in broader RCT NMA** | **Reason not included in primary RCT NMA** |
| --- | --- | --- | --- | --- | --- | --- |
| AibarAlmazan_2019 | FES-I | Fall-related proxy | Lower values better | Sign reversal applied | Yes | Broader-layer outcome; fall-related proxy rather than primary balance/postural-control endpoint |
| Prasertsakul_2018_VR_Pilot | CoP sway area, eyes closed | Instrumented/laboratory postural control | Lower values better | Sign reversal applied | Yes | Broader/pilot evidence only |

**Supplementary Table S12D. Direction-harmonization rules for lower-is-better outcomes**

| **Outcome** | **Studies using the outcome** | **Original interpretation** | **Harmonization rule** | **Interpretation after harmonization** |
| --- | --- | --- | --- | --- |
| EO CoP-x | Lee_and_Yoo_2017 | Lower center-of-pressure displacement indicates better postural control | Mean values were sign-reversed; SDs unchanged | Higher harmonized values indicate better postural control |
| FES-I | AibarAlmazan_2019 | Lower fear-of-falling score indicates better fall-related status | Mean values were sign-reversed; SDs unchanged | Higher harmonized values indicate better fall-related status |
| CoP sway area, eyes closed | Prasertsakul_2018_VR_Pilot | Lower sway area indicates better postural control | Mean values were sign-reversed; SDs unchanged | Higher harmonized values indicate better postural control |
| Other higher-is-better outcomes | BBS, Mini-BESTest, FABS, FRT, SEBT, Stork test, LOS, SOT-6 equilibrium score | Higher values indicate better balance/postural-control performance | No reversal | Higher values indicate better performance |

**Supplementary Table S12E. Clinical/field-based balance-performance outcome sensitivity rule**

| **Sensitivity rule** | **Included outcomes** | **Excluded outcomes** | **Rationale** |
| --- | --- | --- | --- |
| Clinical/field-based balance-performance sensitivity | BBS, Mini-BESTest, FABS, FRT, SEBT, Stork test | LOS, EO CoP-x, SOT-6 equilibrium score, CoP sway area, FES-I | To examine whether primary estimates were sensitive to inclusion of instrumented/laboratory postural-control outcomes and fall-related proxy outcomes |
| Primary randomized-trial NMA | Clinical/field-based balance-performance outcomes and instrumented/laboratory postural-control outcomes | Fall-related proxy outcomes | To synthesize eligible balance and postural-control endpoints while excluding broader fall-related proxy evidence from primary inference |
| Broader randomized-trial NMA | Primary-network outcomes plus prespecified broader/pilot or fall-related proxy evidence | Non-compatible or non-balance outcomes | To examine directional sensitivity after adding broader-layer evidence without replacing primary inference |

**Supplementary Table S12F. Outcome-domain distribution across the final networks**

| **Network layer** | **Balance-performance outcomes** | **Instrumented/laboratory postural-control outcomes** | **Fall-related proxy outcomes** | **Interpretation** |
| --- | --- | --- | --- | --- |
| Primary randomized-trial NMA | Abdelraouf_2020; Bacha_2018_Kinect; Duff_2017_Pilates_MS; Hadas_2024; Junata_2021; Kalron_2016; Lee_2023_RingFit; Molhemi_2021_VR_MS; Sato_2015 | Eldemir_2024; Lee_and_Yoo_2017; Yen_2011_VR_PD | None | Primary inference combines clinical/field-based balance performance and instrumented/laboratory postural-control outcomes |
| Broader randomized-trial NMA | Primary balance-performance studies plus no additional broader-only balance-performance study | Primary instrumented studies plus Prasertsakul_2018_VR_Pilot | AibarAlmazan_2019 | Broader layer adds pilot/instrumented evidence and fall-related proxy evidence |
| Clinical/field-based balance-performance sensitivity | Abdelraouf_2020; Bacha_2018_Kinect; Duff_2017_Pilates_MS; Hadas_2024; Junata_2021; Kalron_2016; Lee_2023_RingFit; Molhemi_2021_VR_MS; Sato_2015 | Excluded | Excluded | Used to evaluate outcome-domain sensitivity |

Note. BBS, Berg Balance Scale; CoP, center of pressure; EO CoP-x, eyes-open center-of-pressure displacement in the x-axis; FABS, Fullerton Advanced Balance Scale; FES-I, Falls Efficacy Scale-International; FRT, Functional Reach Test; LOS, limits of stability; NMA, network meta-analysis; RCT, randomized controlled trial; SD, standard deviation; SEBT, Star Excursion Balance Test; SMD, standardized mean difference; SOT, Sensory Organization Test. Direction harmonization was performed before effect-size construction. For lower-is-better outcomes, mean values were sign-reversed while SDs were unchanged. Positive Hedges’ g values consistently indicate better balance-, postural-control-, or fall-related performance. The clinical/field-based balance-performance sensitivity analysis was additional and exploratory.

**Supplementary Table S13. Pairwise Hedges’ g construction for the revised randomized-trial networks**

**Supplementary Table S13A. Effect-size construction rules**

| **Item** | **Rule used in the revised analysis** |
| --- | --- |
| Effect metric | Hedges’ g standardized mean difference |
| Source data | Harmonized post-intervention arm-level n, mean, and SD |
| Small-sample correction | Exact small-sample correction applied using method.smd = "Hedges" and exact.smd = TRUE |
| Standardizing SD | Pooled post-intervention within-arm SD |
| Direction rule | Outcomes were harmonized before effect-size construction so that higher values represented better balance-, postural-control-, or fall-related performance |
| Lower-is-better outcomes | Mean values were sign-reversed before effect-size construction; SDs were unchanged |
| Multi-arm trials | Multi-arm correlations were handled in the network model |
| Same-node arms | Same-node arms within one randomized trial were combined before contrast construction |
| Between-study variance in NMA | REML estimator used in the random-effects network model |
| Interpretation of pairwise Hedges’ g | Pairwise values are reported as treatment 1 versus treatment 2. Positive values favour treatment 1; negative values favour treatment 2 |
| Role in manuscript | These pairwise estimates are construction inputs for the network model and should not be interpreted as independent confirmatory pairwise meta-analytic conclusions |

**Supplementary Table S13B. Pairwise Hedges’ g contrasts entering the primary randomized-trial NMA**

| **Study/report ID** | **Treatment 1** | **Treatment 2** | **Hedges’ g** | **SE** | **95% CI** | **n1** | **n2** | **Mean1** | **Mean2** | **SD1** | **SD2** | **Interpretation** |
| --- | --- | --- | --- | --- | --- | --- | --- | --- | --- | --- | --- | --- |
| Abdelraouf_2020 | Conventional exercise | Technology-assisted exercise | −0.60 | 0.29 | −1.17 to −0.03 | 25 | 25 | 86.40 | 94.40 | 13.39 | 12.95 | Favours technology-assisted exercise |
| Bacha_2018_Kinect | Conventional exercise | Technology-assisted exercise | 0.07 | 0.29 | −0.51 to 0.64 | 23 | 23 | 29.82 | 29.65 | 2.08 | 2.87 | No clear direction |
| Duff_2017_Pilates_MS | Control | Pilates | −0.07 | 0.37 | −0.78 to 0.65 | 15 | 15 | 30.20 | 31.00 | 13.30 | 9.20 | No clear direction; direct Pilates-control evidence sparse |
| Eldemir_2024 | Control | Technology-delivered Pilates | −0.91 | 0.39 | −1.67 to −0.15 | 15 | 15 | 47.93 | 61.86 | 16.90 | 12.51 | Favours technology-delivered Pilates |
| Hadas_2024 | Pilates | Technology-delivered Pilates | 0.10 | 0.32 | −0.54 to 0.73 | 19 | 19 | 3.50 | 3.30 | 1.70 | 2.30 | No clear direction |
| Junata_2021 | Conventional exercise | Technology-assisted exercise | −0.27 | 0.37 | −0.99 to 0.45 | 14 | 16 | 52.00 | 52.75 | 2.96 | 2.46 | No clear direction |
| Kalron_2016 | Conventional exercise | Pilates | −0.57 | 0.30 | −1.17 to 0.03 | 23 | 22 | 44.60 | 47.90 | 4.90 | 6.40 | Favours Pilates, but CI includes null |
| Lee_2023_RingFit | Control | Technology-assisted exercise | −0.77 | 0.28 | −1.31 to −0.23 | 29 | 28 | 44.86 | 48.18 | 4.70 | 3.71 | Favours technology-assisted exercise |
| Lee_and_Yoo_2017 | Conventional exercise | Technology-assisted exercise | −0.58 | 0.40 | −1.35 to 0.20 | 20 | 10 | −16.86 | −15.90 | 1.34 | 2.07 | Favours technology-assisted exercise, but CI includes null |
| Molhemi_2021_VR_MS | Conventional exercise | Technology-assisted exercise | −0.58 | 0.33 | −1.22 to 0.06 | 20 | 19 | 49.90 | 52.40 | 5.50 | 2.10 | Favours technology-assisted exercise, but CI includes null |
| Sato_2015 | Control | Technology-assisted exercise | −0.75 | 0.28 | −1.31 to −0.20 | 26 | 28 | 30.82 | 33.88 | 4.08 | 3.94 | Favours technology-assisted exercise |
| Yen_2011_VR_PD | Control | Conventional exercise | −0.64 | 0.39 | −1.41 to 0.12 | 14 | 14 | 42.10 | 55.00 | 19.47 | 19.47 | Favours conventional exercise, but CI includes null |
| Yen_2011_VR_PD | Control | Technology-assisted exercise | −0.61 | 0.39 | −1.37 to 0.15 | 14 | 14 | 42.10 | 54.30 | 19.47 | 19.47 | Favours technology-assisted exercise, but CI includes null |
| Yen_2011_VR_PD | Conventional exercise | Technology-assisted exercise | 0.03 | 0.38 | −0.71 to 0.78 | 14 | 14 | 55.00 | 54.30 | 19.47 | 19.47 | No clear direction |

**Supplementary Table S13C. Additional pairwise Hedges’ g contrasts entering only the broader randomized-trial NMA**

| **Study/report ID** | **Treatment 1** | **Treatment 2** | **Hedges’ g** | **SE** | **95% CI** | **n1** | **n2** | **Mean1** | **Mean2** | **SD1** | **SD2** | **Interpretation** |
| --- | --- | --- | --- | --- | --- | --- | --- | --- | --- | --- | --- | --- |
| AibarAlmazan_2019 | Control | Pilates | −0.91 | 0.20 | −1.31 to −0.51 | 52 | 55 | −27.90 | −22.07 | 6.95 | 5.73 | Favours Pilates in broader fall-related proxy layer |
| Prasertsakul_2018_VR_Pilot | Conventional exercise | Technology-assisted exercise | −0.54 | 0.73 | −1.97 to 0.89 | 4 | 4 | −128.04 | −90.34 | 56.91 | 64.55 | No clear direction; very small pilot evidence |

**Supplementary Table S13D. Pairwise contrast accounting**

| **Analysis layer** | **Studies contributing pairwise contrasts** | **Direct pairwise contrasts** | **Notes** |
| --- | --- | --- | --- |
| Primary randomized-trial NMA | 12 | 14 | Includes multi-arm contrasts from Yen_2011_VR_PD and same-node arm combination in Lee_and_Yoo_2017 before contrast construction |
| Broader randomized-trial NMA | 14 | 16 | Includes all primary randomized-trial contrasts plus AibarAlmazan_2019 and Prasertsakul_2018_VR_Pilot |
| Nonrandomized controlled evidence | 1 | 0 in randomized-trial NMA | Not entered into primary or broader randomized-trial effect-size construction |
| Reports without compatible quantitative input | 38 reports | 0 | Not entered into pairwise Hedges’ g construction |

Supplementary Table S13E. Interpretation boundaries for pairwise Hedges’ g inputs

| **Issue** | **Revised handling** | **Interpretation consequence** |
| --- | --- | --- |
| Pairwise estimates are model inputs | Pairwise Hedges’ g values were used as direct contrast inputs for the network model | They should not be interpreted as independent confirmatory pairwise meta-analytic conclusions |
| Negative estimates | Negative values indicate that treatment 2 had better harmonized post-intervention performance than treatment 1 | Direction depends on the treatment ordering in the table |
| Sparse direct comparisons | Several direct comparisons were informed by only one study | Direct evidence was interpreted cautiously and integrated with direct–indirect evidence diagnostics |
| Multi-arm study correlations | Multi-arm correlations were handled in the network model | Pairwise rows from multi-arm trials were not treated as fully independent in the NMA |
| Same-node arms | Same-node comparator arms were combined before contrast construction | Prevents double-counting within a single study |
| Clinical interpretability of SMD | Hedges’ g standardizes heterogeneous outcome scales | Effect magnitude should not be directly translated into scale-specific clinical importance without caution |
| Certainty | Certainty was judged at the comparison level using GRADE/CINeMA-informed methods | Pairwise effect size alone did not determine certainty |

**Note.** CI, confidence interval; NMA, network meta-analysis; RCT, randomized controlled trial; SD, standard deviation; SE, standard error. Pairwise Hedges’ g values were calculated from harmonized post-intervention arm-level n, mean, and SD. Positive Hedges’ g values favour treatment 1 over treatment 2; negative values favour treatment 2 over treatment 1. Lower-is-better outcomes were sign-reversed before effect-size construction. Hedges’ g values were calculated with exact small-sample correction. Multi-arm correlations were handled within the network model. Nonrandomized controlled evidence was not entered into the randomized-trial pairwise effect-size dataset.

**Supplementary Table S14. Primary randomized-trial network meta-analysis estimates**

**Supplementary Table S14A. Primary randomized-trial NMA model specification**

| **Model component** | **Specification used in the revised primary analysis** |
| --- | --- |
| Evidence layer | Primary randomized-trial network |
| Included studies | 12 source-verified randomized trials |
| Source arms | 26 randomized source arms |
| Analytic arms | 25 analytic arms after same-node arm combination |
| Participants | 491 participants |
| Effect metric | Hedges’ g standardized mean difference |
| Direction of effect | Positive values favour the row intervention or active intervention over the comparator after outcome-direction harmonization |
| Source data | Harmonized post-intervention arm-level n, mean, and SD |
| Small-sample correction | Exact Hedges’ g correction |
| Primary model | Random-effects frequentist network meta-analysis |
| Between-study variance estimator | Restricted maximum likelihood |
| Reference node for main estimates | Control |
| Multi-arm trials | Multi-arm correlations handled within the network model |
| Same-node arms | Same-node arms within Lee_and_Yoo_2017 were combined before contrast construction |
| Nonrandomized controlled evidence | Not included in the primary randomized-trial NMA |
| Ranking interpretation | P-score and league-table outputs were exploratory only and not interpreted as a definitive treatment hierarchy |

**Supplementary Table S14B. Primary randomized-trial NMA estimates versus control**

| **Intervention node versus control** | **Hedges’ g** | **95% CI** | **Direction of point estimate** | **Primary interpretation** |
| --- | --- | --- | --- | --- |
| Conventional exercise | 0.33 | −0.04 to 0.69 | Favourable point estimate versus control | Confidence interval includes the null; estimate should be interpreted cautiously |
| Pilates | 0.64 | 0.18 to 1.09 | Favourable estimate versus control | Estimate favours Pilates versus control, but evidence is sparse and certainty is limited |
| Technology-assisted exercise | 0.69 | 0.38 to 1.01 | Favourable estimate versus control | Estimate favours technology-assisted exercise versus control, but the node is broad and heterogeneous |
| Technology-delivered Pilates | 0.69 | 0.14 to 1.25 | Favourable estimate versus control | Estimate favours technology-delivered Pilates versus control, but the node is sparse |

**Supplementary Table S14C. Interpretation of active-intervention comparisons in the primary randomized-trial network**

| **Interpretation issue** | **Revised interpretation** |
| --- | --- |
| Active-intervention hierarchy | The primary randomized-trial network did not establish a definitive hierarchy among active interventions |
| Overlapping uncertainty | Confidence intervals for active interventions versus control overlapped substantially |
| Conventional exercise versus control | Conventional exercise had a favourable point estimate, but its confidence interval included the null |
| Technology-assisted exercise | The estimate should be interpreted as an average effect for a broad digitally augmented exercise category, not as platform-specific evidence for VR, AR, Kinect, exergaming, or any single device |
| Technology-delivered Pilates | The estimate was based on sparse node evidence and should be considered preliminary |
| Pilates | Pilates estimates were limited by sparse direct evidence and a potential local direct–indirect disagreement for Pilates versus control identified in node-splitting diagnostics |
| P-score or ranking | Ranking metrics were retained only as exploratory supplementary outputs and were not used to infer superiority |
| Certainty | Certainty was assessed separately using the GRADE/CINeMA-informed framework and should not be inferred from point estimates alone |

**Supplementary Table S14D. Primary randomized-trial network evidence structure**

| **Direct comparison in primary RCT network** | **Direct evidence available?** | **Main studies contributing direct evidence** | **Interpretation note** |
| --- | --- | --- | --- |
| Technology-assisted exercise vs conventional exercise | Yes | Abdelraouf_2020; Bacha_2018_Kinect; Junata_2021; Lee_and_Yoo_2017; Molhemi_2021_VR_MS; Yen_2011_VR_PD | Most populated direct comparison, but clinically heterogeneous across populations, technologies, and comparator content |
| Technology-assisted exercise vs control | Yes | Lee_2023_RingFit; Sato_2015; Yen_2011_VR_PD | Direct evidence available, but control subtype and population context varied |
| Pilates vs control | Yes | Duff_2017_Pilates_MS | Sparse direct evidence; potential direct–indirect disagreement identified in primary network |
| Technology-delivered Pilates vs control | Yes | Eldemir_2024 | Sparse direct evidence from one RCT |
| Technology-delivered Pilates vs Pilates | Yes | Hadas_2024 | Sparse direct evidence from one RCT |
| Pilates vs conventional exercise | Yes | Kalron_2016 | Sparse direct evidence from one RCT |
| Conventional exercise vs control | Yes | Yen_2011_VR_PD | Sparse direct evidence from one RCT |
| Technology-assisted exercise vs Pilates | No direct primary RCT evidence | Indirect only | Estimate depends on transitivity across heterogeneous direct comparisons |
| Technology-assisted exercise vs technology-delivered Pilates | No direct primary RCT evidence | Indirect only | Estimate depends on sparse and heterogeneous indirect pathways |
| Conventional exercise vs technology-delivered Pilates | No direct primary RCT evidence | Indirect only | Estimate depends on indirect evidence and sparse technology-delivered Pilates node |

**Supplementary Table S14E. Main limitations of the primary randomized-trial NMA estimates**

| **Limitation** | **How it affected interpretation** |
| --- | --- |
| Sparse network | Several comparisons were based on one direct study or indirect-only evidence |
| Clinical heterogeneity | Populations included older adults, athletic low-back-pain participants, healthy sedentary adults, and neurological rehabilitation groups |
| Broad technology-assisted node | Technology-assisted exercise combined VR, AR, Kinect/motion-sensing, exergaming, and active video-game formats |
| Comparator heterogeneity | Control and conventional exercise comparators varied in content, contact, and activity level |
| Outcome-domain heterogeneity | The primary network included both clinical/field-based balance outcomes and instrumented/laboratory postural-control outcomes |
| Post-intervention effect construction | Estimates used post-intervention Hedges’ g because change-score SDs and adjusted estimates were not consistently available |
| Baseline imbalance | Baseline comparability was audited separately, and an additional exclusion sensitivity analysis was performed for the study with potentially consequential selected-outcome baseline difference |
| Inconsistency assessment | Global tests had limited power in the sparse network; local direct–indirect evidence was examined separately |
| Certainty | Certainty was low or very low for principal comparisons and was assessed separately from point estimates |

Note. CI, confidence interval; NMA, network meta-analysis; RCT, randomized controlled trial; VR, virtual reality; AR, augmented reality. Estimates are random-effects network estimates from the source-verified primary randomized-trial network. Hedges’ g values were calculated after direction harmonization so that positive values indicate better balance or postural-control performance. The primary network included 12 randomized trials, 26 randomized source arms represented as 25 analytic arms after same-node arm combination, and 491 participants. Estimates should not be interpreted as treatment ranking or definitive evidence of superiority.

**Supplementary Table S15. Broader randomized-trial network meta-analysis estimates**

**Supplementary Table S15A. Broader randomized-trial NMA model specification**

| **Model component** | **Specification used in the broader randomized-trial analysis** |
| --- | --- |
| Evidence layer | Broader randomized-trial network |
| Included studies | 14 source-verified randomized trials |
| Source arms | 30 randomized source arms |
| Analytic arms | 29 analytic arms after same-node arm combination |
| Participants | 606 participants |
| Relationship to primary analysis | Sensitivity evidence layer; did not replace the primary randomized-trial NMA |
| Studies added beyond the primary network | AibarAlmazan_2019 and Prasertsakul_2018_VR_Pilot |
| Type of added evidence | Prespecified broader-layer randomized evidence, including fall-related proxy evidence and pilot/broader postural-control evidence |
| Effect metric | Hedges’ g standardized mean difference |
| Direction of effect | Positive values favour the row intervention or active intervention over comparator after outcome-direction harmonization |
| Source data | Harmonized post-intervention arm-level n, mean, and SD |
| Small-sample correction | Exact Hedges’ g correction |
| Primary model for broader layer | Random-effects frequentist network meta-analysis |
| Between-study variance estimator | Restricted maximum likelihood |
| Reference node for main estimates | Control |
| Multi-arm trials | Multi-arm correlations handled within the network model |
| Same-node arms | Same-node arms within Lee_and_Yoo_2017 were combined before contrast construction |
| Nonrandomized controlled evidence | Not included in the broader randomized-trial NMA |
| Interpretation | Used to examine directional sensitivity after adding broader randomized evidence; not used to establish confirmatory inference or a definitive treatment hierarchy |

**Supplementary Table S15B. Broader randomized-trial NMA estimates versus control**

| **Intervention node versus control** | **Hedges’ g** | **95% CI** | **Direction of point estimate** | **Broader-layer interpretation** |
| --- | --- | --- | --- | --- |
| Conventional exercise | 0.37 | 0.02 to 0.72 | Favourable estimate versus control | Estimate favours conventional exercise in the broader randomized-trial network, but this did not replace the primary inference and certainty remained limited |
| Pilates | 0.79 | 0.49 to 1.09 | Favourable estimate versus control | Estimate increased after adding broader-layer Pilates evidence based partly on fall-related proxy evidence; interpreted as sensitivity evidence only |
| Technology-assisted exercise | 0.72 | 0.42 to 1.03 | Favourable estimate versus control | Estimate remained favourable after adding broader randomized evidence, but the node remained broad and heterogeneous |
| Technology-delivered Pilates | 0.78 | 0.27 to 1.30 | Favourable estimate versus control | Estimate remained favourable but the node remained sparse and delivery-mode-specific inference was not supported |

**Supplementary Table S15C. Comparison of primary and broader randomized-trial estimates versus control**

| **Intervention node versus control** | **Primary RCT Hedges’ g** | **Primary RCT 95% CI** | **Broader RCT Hedges’ g** | **Broader RCT 95% CI** | **Interpretation of change** |
| --- | --- | --- | --- | --- | --- |
| Conventional exercise | 0.33 | −0.04 to 0.69 | 0.37 | 0.02 to 0.72 | Direction remained favourable; broader-layer inclusion slightly shifted the interval away from the null, but this did not change the primary conclusion |
| Pilates | 0.64 | 0.18 to 1.09 | 0.79 | 0.49 to 1.09 | Estimate increased after adding broader-layer fall-related proxy evidence; this was not interpreted as stronger primary balance/postural-control evidence |
| Technology-assisted exercise | 0.69 | 0.38 to 1.01 | 0.72 | 0.42 to 1.03 | Estimate remained similar and favourable; technology-assisted exercise remained a broad digitally supported exercise node |
| Technology-delivered Pilates | 0.69 | 0.14 to 1.25 | 0.78 | 0.27 to 1.30 | Estimate remained favourable, but sparse node evidence and delivery-mode indirectness persisted |

**Supplementary Table S15D. Added broader-layer randomized studies and their influence on interpretation**

| **Added study** | **Added comparison** | **Added outcome** | **Outcome domain** | **Reason for broader-only allocation** | **Interpretation consequence** |
| --- | --- | --- | --- | --- | --- |
| AibarAlmazan_2019 | Pilates vs control | FES-I | Fall-related proxy | Outcome was a fall-related proxy rather than a direct balance-performance or postural-control endpoint | Increased the broader Pilates-versus-control estimate but introduced outcome-domain heterogeneity |
| Prasertsakul_2018_VR_Pilot | Technology-assisted exercise vs conventional exercise | CoP sway area, eyes closed | Instrumented/laboratory postural control | Pilot/broader-layer randomized evidence with very small sample | Added broader technology-assisted versus conventional evidence but contributed sparse and imprecise information |

**Supplementary Table S15E. Evidence-structure interpretation in the broader randomized-trial NMA**

| **Issue** | **Broader-layer handling** | **Interpretation** |
| --- | --- | --- |
| Added fall-related proxy evidence | FES-I evidence entered only the broader randomized-trial network | Broader estimates were not treated as primary balance/postural-control estimates |
| Added pilot evidence | Very small randomized pilot evidence was retained only in the broader layer | Used for sensitivity mapping, not definitive inference |
| Primary versus broader inference | Primary RCT estimates remained the main inferential results | Broader estimates were used only to examine whether directional patterns changed after adding broader randomized evidence |
| Technology-assisted node | Remained broad after adding broader-layer evidence | No platform-specific inference was made |
| Pilates estimate | Increased in the broader network after adding fall-related proxy evidence | Interpreted cautiously because outcome-domain differences may influence standardized estimates |
| Conventional exercise estimate | Became slightly more favourable in the broader layer | Interpreted as sensitivity evidence only, not as a new primary conclusion |
| Technology-delivered Pilates estimate | Remained based on sparse technology-delivered Pilates evidence | Broader network did not resolve the sparse evidence base for technology-delivered Pilates |
| Treatment ranking | P-score summaries remained exploratory | Broader network was not used to establish a best intervention |

**Note.** CI, confidence interval; CoP, center of pressure; FES-I, Falls Efficacy Scale-International; NMA, network meta-analysis; RCT, randomized controlled trial. Estimates are random-effects network estimates from the broader randomized-trial network. The broader network included the 12 primary randomized trials plus 2 additional broader-layer randomized studies, for a total of 14 studies, 30 randomized source arms represented as 29 analytic arms, and 606 participants. Hedges’ g values were calculated after outcome-direction harmonization so that positive values indicate better balance-, postural-control-, or fall-related performance. The broader randomized-trial network was a sensitivity evidence layer and did not replace the primary randomized-trial NMA.

**Supplementary Table S16. Exploratory P-score summaries in fixed conceptual order**

**Supplementary Table S16A. Interpretation rules for P-score summaries**

| **Item** | Rule used in the final review |
| --- | --- |
| Purpose of P-score | Exploratory numerical summary of relative ordering within a fitted network |
| Main inference | P-scores were not used to determine superiority, certainty, clinical recommendation, or treatment hierarchy |
| Display order | Nodes were displayed in a fixed conceptual order rather than ranked by P-score |
| Conceptual node order | Control; conventional exercise; Pilates; technology-assisted exercise; technology-delivered Pilates |
| Primary interpretation | Comparative estimates versus control, confidence intervals, direct/indirect evidence structure, transitivity, and certainty ratings were prioritized over P-scores |
| Certainty link | P-scores do not incorporate risk of bias, indirectness, imprecision, inconsistency, or data-availability concerns |
| Clinical interpretation | P-scores should not be used to select a specific intervention format, technology platform, device, or delivery model |
| Reporting boundary | P-scores were retained only in supplementary materials and were not used in the abstract, conclusion, or principal findings as evidence of a best intervention |

**Supplementary Table S16B. Exploratory P-score summary from the primary randomized-trial network**

| **Conceptual order** | **Intervention node** | **P-score** | **Display note** |
| --- | --- | --- | --- |
| 1 | Control | 0.012 | Exploratory only; displayed in fixed conceptual order |
| 2 | Conventional exercise | 0.293 | Exploratory only; not interpreted as ranking |
| 3 | Pilates | 0.683 | Exploratory only; not interpreted as superiority |
| 4 | Technology-assisted exercise | 0.771 | Exploratory only; broad heterogeneous technology node |
| 5 | Technology-delivered Pilates | 0.741 | Exploratory only; sparse node evidence |

**Supplementary Table S16C. Exploratory P-score summary from the broader randomized-trial network**

| **Conceptual order** | **Intervention node** | **P-score** | **Display note** |
| --- | --- | --- | --- |
| 1 | Control | 0.005 | Exploratory only; displayed in fixed conceptual order |
| 2 | Conventional exercise | 0.273 | Exploratory only; not interpreted as ranking |
| 3 | Pilates | 0.781 | Exploratory only; influenced by broader-layer fall-related proxy evidence |
| 4 | Technology-assisted exercise | 0.697 | Exploratory only; broad heterogeneous technology node |
| 5 | Technology-delivered Pilates | 0.745 | Exploratory only; sparse node evidence |

**Supplementary Table S16D. Primary versus broader P-score comparison**

| **Intervention node** | **Primary randomized-trial P-score** | **Broader randomized-trial P-score** | **Interpretation of change** |
| --- | --- | --- | --- |
| Control | 0.012 | 0.005 | Control remained lowest in relative ordering, but P-score was not used for inference |
| Conventional exercise | 0.293 | 0.273 | Similar low-to-intermediate exploratory ordering across networks |
| Pilates | 0.683 | 0.781 | Increased after adding broader-layer evidence, including fall-related proxy evidence; this did not replace primary inference |
| Technology-assisted exercise | 0.771 | 0.697 | Remained high in exploratory ordering, but the node was broad and certainty remained limited |
| Technology-delivered Pilates | 0.741 | 0.745 | Similar exploratory ordering across networks, but evidence remained sparse |

**Supplementary Table S16E. Why P-scores were not used for primary interpretation**

| **Limitation of P-score** | Relevance to the final review |
| --- | --- |
| Does not incorporate certainty of evidence | Technology-assisted exercise had a favourable P-score but certainty remained low because of indirectness, sparse evidence, broad technology platforms, and other concerns |
| Does not incorporate transitivity limitations | The network linked heterogeneous populations, interventions, comparators, and outcomes |
| Does not incorporate direct-evidence availability | Some active comparisons were indirect-only or supported by sparse direct evidence |
| Does not account for local incoherence as a certainty judgment | Pilates versus control showed a potential local direct–indirect disagreement in the primary network |
| Does not distinguish technology platforms | VR, AR, Kinect/motion-sensing, exergaming, and active video-game formats were grouped within a broad technology-assisted exercise node |
| Does not address control-node heterogeneity | Control conditions varied across waitlist/no intervention, education-only, usual activity, no physical therapy, and active non-exercise comparator content |
| Does not provide clinical procurement guidance | P-scores cannot identify which technology platform, device, or delivery model should be adopted in practice |
| Sensitive to network structure | Broader P-scores changed after adding broader-layer fall-related proxy or pilot evidence |

Note. P-score values summarize relative ordering within a fitted network and range from 0 to 1, with larger values indicating a higher relative ordering within that specific fitted model. In this review, P-scores were reported only as exploratory supplementary summaries. Nodes were displayed in a fixed conceptual order rather than sorted by P-score. P-scores were not used to establish superiority, treatment hierarchy, certainty of evidence, clinical recommendation, technology procurement decisions, or technology-efficacy claims.

Supplementary Table S17. Sensitivity analyses

**Supplementary Table S17A. Sensitivity-analysis framework**

| **Sensitivity analysis domain** | **Analysis performed** | **Purpose** | **Interpretation boundary** |
| --- | --- | --- | --- |
| Baseline imbalance | Exclusion of the study with potentially consequential selected-outcome baseline difference | To examine whether post-intervention Hedges’ g estimates were sensitive to the study flagged in the baseline audit | Exploratory sensitivity; not a baseline-adjusted NMA |
| Control-node heterogeneity | Non-exercise/minimal-intervention control sensitivity excluding active non-exercise control evidence | To examine whether estimates versus control were sensitive to inclusion of active non-exercise comparator content within the control node | Exploratory structural sensitivity; not a pure placebo/no-contact control analysis |
| Active non-exercise control influence | Exclusion of Duff_2017_Pilates_MS | To examine influence of the active non-exercise Pilates-control study and its effect on direct Pilates-control evidence | Same numerical result as the non-exercise/minimal-intervention control sensitivity; not interpreted as an independent duplicate test |
| Population heterogeneity | Exclusion of athlete/low-back-pain study | To assess influence of the clinically distinct athletic low-back-pain population | Does not resolve remaining clinical heterogeneity across MS, stroke, PD, older-adult, and healthy/sedentary samples |
| Clinical/older-rehabilitation population restriction | Restriction to clinical or older-rehabilitation populations | To examine whether estimates were sensitive to removal of more clinically atypical or healthy/non-rehabilitation evidence | Exploratory; network remained clinically heterogeneous and sparse |
| Outcome-domain heterogeneity | Restriction to clinical/field-based balance-performance outcomes | To examine whether estimates were sensitive to inclusion of instrumented/laboratory postural-control outcomes | Exploratory; important for indirectness and certainty interpretation |
| Pilates-related node definition | Merging of Pilates and technology-delivered Pilates | To evaluate sensitivity to separating versus combining Pilates-content interventions | Does not establish comparable effectiveness between face-to-face and digitally delivered Pilates |
| Modelling assumption | Common-effect model | To compare random-effects and common-effect modelling under sparse network conditions | Similar estimates do not prove clinical homogeneity |
| AR/VR protocol-scope feasibility | AR/VR-only feasibility analysis | To examine whether an AR/VR-restricted subset could be estimated | Feasibility check only; not used as primary inference |
| High-risk/nonrandom evidence | Exclusion of high-risk/nonrandom evidence from RCT-primary set | Addressed by restricting the main analysis to source-verified randomized trials | No additional RCT-primary estimate change because nonrandom evidence was already outside the RCT-primary network |

Supplementary Table S17B. Network geometry of sensitivity analyses

| **Analysis** | **Studies** | **Analytic arms** | **Participants** | **Nodes retained** | **Network connected?** |
| --- | --- | --- | --- | --- | --- |
| Primary randomized-trial NMA | 12 | 25 | 491 | Control; conventional exercise; Pilates; technology-assisted exercise; technology-delivered Pilates | Yes |
| Broader randomized-trial NMA | 14 | 29 | 606 | Control; conventional exercise; Pilates; technology-assisted exercise; technology-delivered Pilates | Yes |
| Pilates-related node merging | 11 | 23 | 453 | Control; conventional exercise; Pilates-related exercise; technology-assisted exercise | Yes |
| Excluding athlete/low-back-pain study | 11 | 23 | 441 | Control; conventional exercise; Pilates; technology-assisted exercise; technology-delivered Pilates | Yes |
| Excluding Duff_2017_Pilates_MS | 11 | 23 | 461 | Control; conventional exercise; Pilates; technology-assisted exercise; technology-delivered Pilates | Yes |
| Non-exercise/minimal-intervention control sensitivity | 11 | 23 | 461 | Control; conventional exercise; Pilates; technology-assisted exercise; technology-delivered Pilates | Yes |
| Excluding potentially consequential baseline imbalance | 11 | 23 | 461 | Control; conventional exercise; Pilates; technology-assisted exercise; technology-delivered Pilates | Yes |
| Clinical/older-rehabilitation population restriction | 10 | 21 | 403 | Control; conventional exercise; Pilates; technology-assisted exercise; technology-delivered Pilates | Yes |
| Clinical/field-based balance-performance outcome restriction | 9 | 18 | 389 | Control; conventional exercise; Pilates; technology-assisted exercise; technology-delivered Pilates | Yes |
| AR/VR-only feasibility analysis | 4 | 9 | 161 | Control; conventional exercise; technology-assisted exercise | Yes |
| Common-effect model | 12 | 25 | 491 | Control; conventional exercise; Pilates; technology-assisted exercise; technology-delivered Pilates | Yes |

**Supplementary Table S17C. Primary and sensitivity estimates versus control**

| **Analysis** | **Conventional exercise vs control** | **Pilates or Pilates-related node vs control** | **Technology-assisted exercise vs control** | **Technology-delivered Pilates vs control** | **Main interpretation** |
| --- | --- | --- | --- | --- | --- |
| Primary randomized-trial NMA | 0.33 [−0.04, 0.69] | 0.64 [0.18, 1.09] | 0.69 [0.38, 1.01] | 0.69 [0.14, 1.25] | Main inferential analysis; active nodes generally favourable, but conventional exercise CI included null and active estimates overlapped |
| Broader randomized-trial NMA | 0.37 [0.02, 0.72] | 0.79 [0.49, 1.09] | 0.72 [0.42, 1.03] | 0.78 [0.27, 1.30] | Broader sensitivity layer; added broader/pilot or fall-related proxy evidence and did not replace primary inference |
| Pilates-related node merging | 0.33 [−0.03, 0.69] | 0.65 [0.23, 1.08] | 0.70 [0.38, 1.01] | Merged with Pilates-related node | Estimates were similar after merging Pilates and technology-delivered Pilates, but this does not establish comparable effectiveness between delivery formats |
| Excluding athlete/low-back-pain study | 0.37 [−0.01, 0.75] | 0.66 [0.20, 1.11] | 0.68 [0.36, 1.00] | 0.71 [0.15, 1.26] | Direction remained broadly favourable; exclusion did not resolve residual clinical heterogeneity |
| Excluding Duff_2017_Pilates_MS | 0.45 [0.06, 0.83] | 1.01 [0.43, 1.60] | 0.77 [0.44, 1.09] | 0.92 [0.32, 1.51] | Estimates increased after removing the direct Pilates-control active non-exercise control study; evidence structure changed and should not be interpreted as robustness proof |
| Non-exercise/minimal-intervention control sensitivity | 0.45 [0.06, 0.83] | 1.01 [0.43, 1.60] | 0.77 [0.44, 1.09] | 0.92 [0.32, 1.51] | Same numerical result as Duff exclusion because the active non-exercise control study was removed; indicates control-node structural sensitivity |
| Excluding potentially consequential baseline imbalance | 0.35 [−0.02, 0.72] | 0.65 [0.19, 1.10] | 0.69 [0.37, 1.00] | 0.70 [0.14, 1.25] | Similar to primary estimates; does not constitute baseline-adjusted NMA |
| Clinical/older-rehabilitation population restriction | 0.34 [−0.05, 0.73] | 0.56 [0.05, 1.08] | 0.66 [0.34, 0.98] | 0.91 [0.15, 1.67] | Estimates remained generally favourable, but population heterogeneity remained across clinical groups |
| Clinical/field-based balance-performance outcome restriction | 0.21 [−0.28, 0.70] | 0.48 [−0.10, 1.05] | 0.63 [0.22, 1.04] | 0.38 [−0.53, 1.29] | Estimates attenuated for conventional exercise, Pilates, and technology-delivered Pilates; only technology-assisted exercise retained a CI excluding null |
| AR/VR-only feasibility analysis | 0.39 [−0.29, 1.08] | Not estimable in restricted network | 0.86 [0.17, 1.54] | Not estimable in restricted network | Feasibility check only; restricted network omitted Pilates and technology-delivered Pilates nodes |
| Common-effect model | 0.33 [−0.04, 0.69] | 0.64 [0.18, 1.09] | 0.69 [0.38, 1.01] | 0.69 [0.14, 1.25] | Same estimates as random-effects model in this sparse network; not interpreted as proof of clinical homogeneity |

**Supplementary Table S17D. Key sensitivity-analysis implications**

| **Sensitivity domain** | **Main finding** | **Interpretation for revised manuscript** |
| --- | --- | --- |
| Baseline imbalance | Exclusion of Lee_and_Yoo_2017 produced only small changes in active-versus-control estimates | Supports that the primary direction was not wholly dependent on the flagged baseline-imbalance study, but does not remove baseline concerns generally |
| Control-node heterogeneity | Removing the active non-exercise control study increased estimates and altered evidence structure | Control-node composition matters; estimates versus control should be interpreted as comparisons against heterogeneous non-exercise/minimally active reference conditions |
| Direct Pilates-control evidence | Removing Duff_2017_Pilates_MS removed the direct Pilates-control link and increased Pilates-related estimates | Pilates-control evidence is structurally fragile and should be interpreted cautiously |
| Athletic low-back-pain study | Excluding Abdelraouf_2020 did not materially change point-estimate direction | The athlete study did not dominate direction, but clinical heterogeneity across other populations remained |
| Clinical/older-rehabilitation restriction | Estimates remained broadly favourable but the network remained sparse and clinically mixed | Does not fully resolve transitivity concerns |
| Outcome-domain restriction | Several estimates attenuated and CIs crossed null after restricting to clinical/field-based balance-performance outcomes | Outcome-domain heterogeneity is important and should inform indirectness/certainty judgments |
| Pilates node merging | Merged Pilates-related node produced similar estimates | Separating face-to-face and digitally delivered Pilates remains conceptually appropriate because delivery mode and implementation differ |
| Common-effect model | Estimates matched random-effects results because between-study variance was estimated near zero | Similar estimates must not be interpreted as evidence of true clinical homogeneity |
| AR/VR-only feasibility | Restricted network was estimable only for control, conventional exercise, and technology-assisted exercise | AR/VR-only evidence was too limited to support the full five-node network |

Supplementary Table S17E. Sensitivity-analysis link to certainty assessment

| Sensitivity finding | Primary certainty domain | Interpretation rule |
| --- | --- | --- |
| Control-node sensitivity changed estimates or evidence structure | Indirectness | Comparator variability was counted under indirectness, not again as heterogeneity or applicability. |
| Pilates-control direct evidence was sparse and discordant with indirect evidence | Incoherence | The local direct-indirect signal was counted as incoherence; sparse evidence was handled under imprecision. |
| Outcome-restricted analysis attenuated several node estimates | Indirectness | Outcome-domain dependence was counted under indirectness. |
| Population-restricted analysis changed network geometry or widened intervals | Indirectness or imprecision | Clinical population differences were indirectness; wider intervals from sparser data were imprecision. |
| Baseline-imbalance exclusion produced similar estimates | Within-study bias | Supported sensitivity interpretation but did not remove within-study/baseline concerns. |
| Common-effect and random-effects estimates were similar | Heterogeneity | No additional heterogeneity downgrade was applied when no residual statistical signal was present. |
| AR/VR-only restricted evidence was incomplete | Indirectness | Protocol-scope and intervention-node breadth were treated as indirectness. |

Note. CI, confidence interval; NMA, network meta-analysis; RCT, randomized controlled trial. Values are Hedges’ g with 95% confidence intervals from sensitivity analyses based on the source-verified randomized-trial network unless otherwise stated. Positive values favour the intervention node over control. Sensitivity analyses were exploratory and were interpreted according to point estimates, confidence intervals, network geometry, direct-evidence structure, and certainty implications rather than binary significance testing. Similar point-estimate directions were not interpreted as proof of robustness, homogeneity, consistency, or treatment superiority.

**Supplementary Table S18. Direct–indirect evidence, inconsistency diagnostics, and contribution-matrix summary**

**Supplementary Table S18A. Diagnostic framework used in the revised randomized-trial networks**

| **Diagnostic component** | **Purpose** | Interpretation rule used in the final review |
| --- | --- | --- |
| Global heterogeneity statistics | To describe whether a statistical between-study heterogeneity signal was detected in the fitted network | Absence of a statistical signal was not interpreted as evidence of clinical homogeneity |
| Q decomposition and design-by-treatment inconsistency | To assess global design-level inconsistency where estimable | Non-significant results were interpreted cautiously because the network was small and sparse |
| Node-splitting/direct–indirect comparison | To compare direct and indirect evidence for estimable comparisons | Direct–indirect disagreement was interpreted as a potential local incoherence signal when detected, not as definitive proof of inconsistency |
| Direct-evidence availability | To identify which network estimates were informed by direct evidence and which were indirect-only | Indirect-only estimates were interpreted as more dependent on the transitivity assumption |
| Random-effects contribution matrix | To identify which direct comparisons contributed most to network estimates | Used to support RoB, indirectness, and certainty judgments; not used for treatment ranking |
| Comparison-adjusted funnel plot | Exploratory visual assessment of small-study/reporting-bias signals | Not treated as a formal publication-bias test because the network contained few direct effects and several sparse comparisons |

**Supplementary Table S18B. Global heterogeneity and design-level inconsistency diagnostics**

| **Network or sensitivity analysis** | **Studies** | **Analytic arms** | **Participants** | **Pairwise rows** | **Treatments** | **tau²** | **I²** | **Q total** | **df** | **Q P value** | **Design-by-treatment P value** | **Interpretation** |
| --- | --- | --- | --- | --- | --- | --- | --- | --- | --- | --- | --- | --- |
| Primary randomized-trial NMA | 12 | 25 | 491 | 14 | 5 | <0.001 | 0.0% | 8.65 | 9 | 0.470 | 0.281 | No statistically clear global signal was detected, but this does not prove clinical homogeneity or consistency |
| Broader randomized-trial NMA | 14 | 29 | 606 | 16 | 5 | <0.001 | 0.0% | 9.51 | 11 | 0.575 | 0.779 | No statistically clear global signal was detected; broader evidence added outcome-domain and population heterogeneity |
| Excluding potentially consequential baseline imbalance | 11 | 23 | 461 | 13 | 5 | <0.001 | 3.9% | 8.32 | 8 | 0.403 | Not used as primary inconsistency inference | Used as an additional baseline-imbalance sensitivity analysis |
| Clinical/older-rehabilitation population restriction | 10 | 21 | 403 | 12 | 5 | 0.001 | 3.2% | 7.23 | 7 | 0.405 | Not used as primary inconsistency inference | Population-restricted analysis remained sparse and clinically heterogeneous |
| Clinical/field-based balance-performance outcome restriction | 9 | 18 | 389 | 9 | 5 | 0.023 | 19.4% | 6.20 | 5 | 0.287 | Not used as primary inconsistency inference | Outcome-restricted analysis showed attenuation for several nodes and non-zero heterogeneity signal |
| Non-exercise/minimal-intervention control sensitivity | 11 | 23 | 461 | 13 | 5 | <0.001 | 0.0% | 4.62 | 8 | 0.798 | Not used as primary inconsistency inference | Control-node sensitivity changed evidence structure and was interpreted as structural sensitivity |
| AR/VR-only feasibility analysis | 4 | 9 | 161 | 6 | 3 | <0.001 | 0.0% | 2.17 | 3 | 0.538 | Not used as primary inconsistency inference | Feasibility analysis only; full five-node network was not retained |
| Pilates-related node-merging sensitivity | 11 | 23 | 453 | 13 | 4 | <0.001 | 0.0% | 8.61 | 9 | 0.474 | Not used as primary inconsistency inference | Node-merging did not establish equivalence between face-to-face and digitally delivered Pilates |

**Supplementary Table S18C. Direct-evidence availability in the primary randomized-trial network**

| **Comparison** | **Direct evidence available?** | **Number of direct studies/contrasts** | **Main direct evidence source(s)** | **Evidence-structure interpretation** |
| --- | --- | --- | --- | --- |
| Conventional exercise vs control | Yes | 1 | Yen_2011_VR_PD | Sparse direct evidence from one neurological rehabilitation trial |
| Pilates vs control | Yes | 1 | Duff_2017_Pilates_MS | Sparse direct evidence from one RCT; potential direct–indirect disagreement identified |
| Technology-assisted exercise vs control | Yes | 3 | Lee_2023_RingFit; Sato_2015; Yen_2011_VR_PD | Direct evidence available, but control subtype and population context varied |
| Technology-delivered Pilates vs control | Yes | 1 | Eldemir_2024 | Sparse direct evidence from one RCT |
| Conventional exercise vs Pilates | Yes | 1 | Kalron_2016 | Sparse direct evidence from one MS trial |
| Conventional exercise vs technology-assisted exercise | Yes | 6 | Abdelraouf_2020; Bacha_2018_Kinect; Junata_2021; Lee_and_Yoo_2017; Molhemi_2021_VR_MS; Yen_2011_VR_PD | Most populated direct comparison, but clinically heterogeneous across populations, technologies, outcomes, and comparator intensity |
| Conventional exercise vs technology-delivered Pilates | No | 0 | Indirect only | Entirely indirect comparison; strongly dependent on transitivity |
| Pilates vs technology-assisted exercise | No | 0 | Indirect only | Entirely indirect comparison; strongly dependent on transitivity |
| Pilates vs technology-delivered Pilates | Yes | 1 | Hadas_2024 | Sparse direct evidence from one RCT comparing digital and face-to-face Pilates delivery |
| Technology-assisted exercise vs technology-delivered Pilates | No | 0 | Indirect only | Entirely indirect comparison; dependent on sparse technology-delivered Pilates node |

**Supplementary Table S18D. Direct–indirect decomposition in the primary randomized-trial network**

| **Comparison** | **Direct studies** | **Direct evidence contribution** | **Network estimate** | **Direct estimate** | **Indirect estimate** | **Direct–indirect difference** | **P value** | **Evidence-structure interpretation** |
| --- | --- | --- | --- | --- | --- | --- | --- | --- |
| Conventional exercise vs control | 1 | 22.9% | 0.33 [−0.04, 0.69] | 0.64 [−0.12, 1.41] | 0.23 [−0.18, 0.65] | 0.41 [−0.46, 1.28] | 0.354 | No statistically clear direct–indirect disagreement detected |
| Pilates vs control | 1 | 39.9% | 0.64 [0.18, 1.09] | 0.07 [−0.65, 0.78] | 1.01 [0.43, 1.60] | −0.95 [−1.87, −0.02] | 0.045 | Potential local direct–indirect disagreement; interpreted as local incoherence concern |
| Technology-assisted exercise vs control | 3 | 84.4% | 0.69 [0.38, 1.01] | 0.73 [0.39, 1.08] | 0.48 [−0.32, 1.28] | 0.25 [−0.62, 1.12] | 0.579 | No statistically clear direct–indirect disagreement detected |
| Technology-delivered Pilates vs control | 1 | 53.7% | 0.69 [0.14, 1.25] | 0.91 [0.15, 1.67] | 0.44 [−0.37, 1.26] | 0.47 [−0.64, 1.58] | 0.408 | No statistically clear direct–indirect disagreement detected; direct evidence sparse |
| Conventional exercise vs Pilates | 1 | 58.4% | −0.31 [−0.77, 0.15] | −0.57 [−1.17, 0.03] | 0.06 [−0.65, 0.76] | −0.63 [−1.55, 0.30] | 0.185 | No statistically clear direct–indirect disagreement detected |
| Conventional exercise vs technology-assisted exercise | 6 | 91.1% | −0.37 [−0.62, −0.11] | −0.32 [−0.59, −0.05] | −0.80 [−1.66, 0.05] | 0.48 [−0.41, 1.38] | 0.291 | No statistically clear direct–indirect disagreement detected; direct evidence clinically heterogeneous |
| Conventional exercise vs technology-delivered Pilates | 0 | 0.0% | −0.37 [−0.97, 0.24] | Not estimable | −0.37 [−0.97, 0.24] | Not estimable | Not estimable | Indirect-only comparison |
| Pilates vs technology-assisted exercise | 0 | 0.0% | −0.06 [−0.53, 0.42] | Not estimable | −0.06 [−0.53, 0.42] | Not estimable | Not estimable | Indirect-only comparison |
| Pilates vs technology-delivered Pilates | 1 | 67.3% | −0.06 [−0.58, 0.46] | 0.10 [−0.54, 0.73] | −0.37 [−1.28, 0.54] | 0.47 [−0.64, 1.58] | 0.408 | No statistically clear direct–indirect disagreement detected; direct evidence sparse |
| Technology-assisted exercise vs technology-delivered Pilates | 0 | 0.0% | −0.00 [−0.60, 0.60] | Not estimable | −0.00 [−0.60, 0.60] | Not estimable | Not estimable | Indirect-only comparison |

**Supplementary Table S18E. Direct–indirect decomposition in the broader randomized-trial network**

| **Comparison** | **Direct studies** | **Direct evidence contribution** | **Network estimate** | **Direct estimate** | **Indirect estimate** | **Direct–indirect difference** | **P value** | **Evidence-structure interpretation** |
| --- | --- | --- | --- | --- | --- | --- | --- | --- |
| Conventional exercise vs control | 1 | 20.8% | 0.37 [0.02, 0.72] | 0.64 [−0.12, 1.41] | 0.30 [−0.09, 0.69] | 0.34 [−0.51, 1.20] | 0.433 | No statistically clear direct–indirect disagreement detected |
| Pilates vs control | 2 | 73.7% | 0.79 [0.49, 1.09] | 0.71 [0.36, 1.06] | 1.01 [0.43, 1.59] | −0.30 [−0.98, 0.38] | 0.388 | Primary-network Pilates-control signal was not statistically clear in broader network; broader evidence includes fall-related proxy |
| Technology-assisted exercise vs control | 3 | 80.4% | 0.72 [0.42, 1.03] | 0.73 [0.39, 1.08] | 0.70 [0.00, 1.40] | 0.03 [−0.75, 0.81] | 0.937 | No statistically clear direct–indirect disagreement detected |
| Technology-delivered Pilates vs control | 1 | 46.8% | 0.78 [0.27, 1.30] | 0.91 [0.15, 1.67] | 0.67 [−0.04, 1.38] | 0.24 [−0.80, 1.28] | 0.650 | No statistically clear direct–indirect disagreement detected; direct evidence sparse |
| Conventional exercise vs Pilates | 1 | 43.2% | −0.42 [−0.81, −0.03] | −0.57 [−1.17, 0.03] | −0.30 [−0.82, 0.22] | −0.27 [−1.06, 0.53] | 0.509 | No statistically clear direct–indirect disagreement detected |
| Conventional exercise vs technology-assisted exercise | 7 | 89.2% | −0.35 [−0.60, −0.10] | −0.33 [−0.59, −0.07] | −0.55 [−1.31, 0.21] | 0.22 [−0.58, 1.02] | 0.593 | No statistically clear direct–indirect disagreement detected; direct evidence clinically heterogeneous |
| Conventional exercise vs technology-delivered Pilates | 0 | 0.0% | −0.41 [−1.01, 0.18] | Not estimable | −0.41 [−1.01, 0.18] | Not estimable | Not estimable | Indirect-only comparison |
| Pilates vs technology-assisted exercise | 0 | 0.0% | 0.07 [−0.32, 0.45] | Not estimable | 0.07 [−0.32, 0.45] | Not estimable | Not estimable | Indirect-only comparison |
| Pilates vs technology-delivered Pilates | 1 | 62.4% | 0.01 [−0.50, 0.51] | 0.10 [−0.54, 0.73] | −0.14 [−0.96, 0.68] | 0.24 [−0.80, 1.28] | 0.650 | No statistically clear direct–indirect disagreement detected; direct evidence sparse |
| Technology-assisted exercise vs technology-delivered Pilates | 0 | 0.0% | −0.06 [−0.64, 0.53] | Not estimable | −0.06 [−0.64, 0.53] | Not estimable | Not estimable | Indirect-only comparison |

**Supplementary Table S18F. Random-effects contribution-matrix summary for key primary-network estimates**

| **Network estimate** | **Largest contribution sources** | **Contribution pattern and interpretation** |
| --- | --- | --- |
| Technology-assisted exercise vs control | Technology-assisted exercise vs control direct evidence contributed 78.4%; technology-assisted exercise vs conventional exercise contributed 8.4%; control vs conventional exercise contributed 4.7% | Estimate was primarily informed by direct evidence, but direct evidence came from heterogeneous technology platforms, populations, and control subtypes |
| Pilates vs control | Pilates vs control direct evidence contributed 39.9%; conventional exercise vs Pilates contributed 14.3%; technology-assisted exercise vs control contributed 10.6%; technology-assisted exercise vs conventional exercise contributed 10.6%; technology-delivered Pilates vs control contributed 10.5% | Estimate depended substantially on both sparse direct evidence and heterogeneous indirect pathways; supports cautious incoherence and indirectness judgment |
| Conventional exercise vs control | Technology-assisted exercise vs control contributed 32.9%; technology-assisted exercise vs conventional exercise contributed 32.9%; direct conventional exercise vs control contributed 15.0%; conventional exercise vs Pilates contributed 8.5% | Estimate relied heavily on indirect paths through technology-assisted exercise and conventional-comparator evidence |
| Technology-delivered Pilates vs control | Technology-delivered Pilates vs control direct evidence contributed 53.7%; Pilates vs technology-delivered Pilates contributed 17.8%; Pilates vs control contributed 11.7% | Estimate was informed by a sparse direct link and indirect Pilates-related pathways |
| Conventional exercise vs technology-assisted exercise | Direct conventional exercise vs technology-assisted exercise evidence contributed 87.4% | Most directly supported active comparison, but direct evidence was clinically heterogeneous |
| Pilates vs technology-delivered Pilates | Direct Pilates vs technology-delivered Pilates evidence contributed 67.3%; indirect pathways contributed the remaining evidence | Sparse direct evidence; no equivalence conclusion was drawn |

Supplementary Table S18G. Certainty implications of direct–indirect and diagnostic findings

| Diagnostic finding | Primary certainty domain | Certainty implication |
| --- | --- | --- |
| Sparse direct evidence for several comparisons | Imprecision | Supported downgrading when CIs were wide, crossed the null, or overlapped the analytical reference range. |
| Indirect-only active comparisons | Indirectness | Reflected dependence on clinical exchangeability assumptions. |
| Potential Pilates-control direct-indirect disagreement | Incoherence | Pilates-control certainty was downgraded for a local inconsistency signal. |
| Heterogeneous technology-assisted contribution sources | Indirectness | Technology-assisted exercise was interpreted as a broad node rather than a platform-specific effect. |
| Heterogeneous control subtypes | Indirectness | Control-node variability was counted under indirectness. |
| Global tests without clear signal | Heterogeneity/incoherence | Absence of a statistically clear global signal was not treated as proof of homogeneity or consistency. |
| Small number of direct effects for funnel-plot assessment | Reporting/data availability | Publication-bias and small-study-effect assessment was descriptive only. |

**Note.** CI, confidence interval; NMA, network meta-analysis; RCT, randomized controlled trial; RoB, risk of bias. Direct–indirect estimates are Hedges’ g values with 95% confidence intervals after outcome-direction harmonization. Comparisons are displayed as treatment 1 versus treatment 2; positive values favour treatment 1 and negative values favour treatment 2. The primary randomized-trial network identified a potential local direct–indirect disagreement for Pilates versus control. Global heterogeneity and design-level inconsistency diagnostics did not detect statistically clear signals, but the network was small and sparse; therefore, absence of a signal was not interpreted as proof of clinical homogeneity or consistency. The random-effects contribution matrix was used to support risk-of-bias, indirectness, incoherence, and certainty judgments, not to rank interventions.

Supplementary Table S19. RoB2 assessment across all source-verified randomized trials and study-design compatibility

Supplementary Table S19A. Risk-of-bias framework used in the final review

| **Evidence type** | **Bias-assessment approach** | **Included in primary randomized-trial certainty?** | **Rationale** |
| --- | --- | --- | --- |
| Source-verified randomized trials in the primary randomized-trial NMA | RoB2 | Yes | Compatible with randomized-trial inference and primary NMA estimates |
| Source-verified randomized trials included only in the broader randomized-trial NMA | RoB2 | Used only for broader/sensitivity interpretation | These studies informed the broader randomized-trial network but did not replace the primary inference |
| Nonrandomized controlled evidence | Not assessed using RoB2 for randomized-trial inference | No | Nonrandom allocation is not compatible with RoB2-based randomized-trial certainty judgments |
| Contextual reports not entering NMA effect-size estimation | Not applicable to NMA risk-of-bias contribution | No | Used only for contextual synthesis or evidence-landscape description |
| Reports without compatible quantitative data | Not applicable to direct effect-size bias contribution | No direct contribution to NMA estimates | Considered under data-availability/reporting-bias concerns rather than study-level RoB contribution |

**Supplementary Table S19B. RoB2 domains and interpretation**

| **RoB2 domain** | **Domain label** | **What the domain assesses** | **How the judgment was used** |
| --- | --- | --- | --- |
| D1 | Bias arising from the randomization process | Random sequence generation, allocation concealment, and baseline imbalances suggesting randomization problems | Informed within-study bias and, where relevant, baseline-imbalance interpretation |
| D2 | Bias due to deviations from intended interventions | Deviations from assigned intervention, adherence, co-intervention, contamination, and differential non-intervention activity | Interpreted alongside concurrent activity and co-intervention audit |
| D3 | Bias due to missing outcome data | Completeness of outcome data and whether missingness could affect intervention comparisons | Informed within-study bias and imprecision/contextual certainty |
| D4 | Bias in measurement of the outcome | Blinding/assessor masking, outcome measurement objectivity, and differential outcome assessment | Considered particularly relevant for performance-based and assessor-dependent outcomes |
| D5 | Bias in selection of the reported result | Selective reporting of outcomes, time points, or analyses | Interpreted together with protocol/reporting transparency and data-availability concerns |
| Overall RoB2 | Overall study-level risk-of-bias judgment | Integrated domain-level judgments according to RoB2 guidance | Used in comparison-level certainty assessment through the random-effects contribution matrix |

Supplementary Table S19C. Study-level RoB2 judgments for all 14 source-verified randomized trials contributing to either randomized-trial evidence layer

| Study ID | Evidence layer | D1 | D2 | D3 | D4 | D5 | Overall |
| --- | --- | --- | --- | --- | --- | --- | --- |
| Abdelraouf_2020 | Primary | Low risk | Some concerns | Low risk | Some concerns | Some concerns | Some concerns |
| Bacha_2018_Kinect | Primary | Some concerns | Some concerns | Low risk | Some concerns | Some concerns | Some concerns |
| Duff_2017_Pilates_MS | Primary | Some concerns | Some concerns | Low risk | Some concerns | Some concerns | Some concerns |
| Eldemir_2024 | Primary | Some concerns | Some concerns | Low risk | Some concerns | Some concerns | Some concerns |
| Hadas_2024 | Primary | Some concerns | Some concerns | Low risk | Some concerns | Some concerns | Some concerns |
| Junata_2021 | Primary | Some concerns | Some concerns | Low risk | Some concerns | Some concerns | Some concerns |
| Kalron_2016 | Primary | Some concerns | Some concerns | Low risk | Some concerns | Some concerns | Some concerns |
| LeeYoo_2017_AR_Otago | Primary | Some concerns | Some concerns | Low risk | Some concerns | Some concerns | Some concerns |
| Lee_2023_RingFit | Primary | Some concerns | Some concerns | Low risk | Some concerns | Some concerns | Some concerns |
| Molhemi_2021_VR_MS | Primary | Low risk | Some concerns | Low risk | Low risk | Low risk | Some concerns |
| Sato_2015 | Primary | Some concerns | Some concerns | Low risk | Some concerns | Some concerns | Some concerns |
| Yen_2011_VR_PD | Primary | Some concerns | Some concerns | Low risk | Some concerns | Some concerns | Some concerns |
| AibarAlmazan_2019 | Broader-only | Low risk | Some concerns | Low risk | Low risk | Low risk | Some concerns |
| Prasertsakul_2018_VR_Pilot | Broader-only | Some concerns | Some concerns | Low risk | Some concerns | Some concerns | Some concerns |

Supplementary Table S19D. Domain-level RoB2 summary corresponding to Figure 5A and Supplementary Figure S5

| RoB2 domain | Low risk | Some concerns | High risk | Total trials |
| --- | --- | --- | --- | --- |
| D1 Randomization process | 3 | 11 | 0 | 14 |
| D2 Deviations from intended interventions | 0 | 14 | 0 | 14 |
| D3 Missing outcome data | 14 | 0 | 0 | 14 |
| D4 Measurement of the outcome | 2 | 12 | 0 | 14 |
| D5 Selection of the reported result | 2 | 12 | 0 | 14 |
| Overall | 0 | 14 | 0 | 14 |

**Supplementary Table S19E. Comparison-level implications of RoB2 judgments**

| Evidence component | Final handling | Implication for interpretation |
| --- | --- | --- |
| Figure 5A and Supplementary Figure S5 | Summarize all 14 source-verified randomized trials | RoB2 evidence is reported across the full randomized evidence layer. |
| Figure 5B primary certainty estimates | Use the 12-study primary randomized-trial network | The two broader-only randomized trials do not contribute to the Figure 5B primary certainty estimates. |
| Overall RoB2 pattern | All 14 trials were judged as having some concerns overall | Within-study limitations contributed to conservative certainty ratings. |
| Nonrandomized controlled evidence | Excluded from randomized-trial RoB2 summaries and primary certainty | No RoB2-based randomized-trial inference was drawn from this study. |

**Supplementary Table S19F. Workflow for risk-of-bias assessment and verification**

| Step | Procedure used in the final review |
| --- | --- |
| Initial assessment | One reviewer completed initial RoB2 domain-level judgments using source reports and the extraction dataset. |
| Verification | A second reviewer checked domain-level and overall judgments against source reports and prespecified RoB2 criteria. |
| Consensus | Unclear or discrepant judgments were resolved through consensus discussion before certainty assessment. |
| Scope alignment | RoB2 summaries cover 14 source-verified randomized trials; primary certainty estimates cover the 12-study primary network. |

**Note.** NMA, network meta-analysis; RCT, randomized controlled trial; RoB, risk of bias; RoB2, revised Cochrane risk-of-bias tool for randomized trials. Overall RoB2 judgments for source-verified randomized trials were “some concerns” in the revised randomized-trial evidence base. The controlled study with nonrandom allocation was retained outside the randomized-trial networks and was not used to determine the primary randomized-trial conclusions. Detailed domain-level RoB2 judgments are presented in the updated risk-of-bias traffic-light figure and retained in the risk-of-bias assessment archive. RoB2 judgments informed comparison-level certainty through the random-effects contribution matrix.

**Supplementary Table S20. GRADE/CINeMA-informed certainty assessment**

Supplementary Table S20A. Certainty-assessment framework used in the final review

| Assessment component | Operationalization in the final review | Role in final certainty judgment |
| --- | --- | --- |
| Starting certainty | Randomized-trial evidence started as high certainty before domain-level concerns were considered. | Downgraded according to comparison-level concerns. |
| Within-study bias | Based on RoB2 judgments for source-verified randomized trials and random-effects contribution patterns. | Rated 0, -1, or -2 levels. |
| Reporting/data availability | Based on unavailable compatible arm-level numerical data and non-contributing eligible reports. | Rated 0, -1, or -2 levels without duplicating imprecision. |
| Indirectness | Based on population/pathology differences, intervention-node breadth, technology-platform heterogeneity, control-node heterogeneity, outcome-domain dependence, and transitivity concerns. | Rated 0, -1, or -2 levels; overlapping clinical concerns were counted here once. |
| Imprecision | Based on 95% CI width, sparse evidence, null crossing, and overlap with the prespecified analytical reference range. | Rated 0, -1, or -2 levels. |
| Heterogeneity | Based on residual statistical heterogeneity not already captured as clinical indirectness. | Rated 0, -1, or -2 levels. |
| Incoherence | Based on direct-indirect evidence where estimable, including node-splitting results. | Rated 0, -1, or -2 levels. |
| Avoiding double counting | Overlapping concerns were assigned to the most relevant single domain. | The same concern was not downgraded in multiple domains. |
| Final certainty | Total downgrades were mapped to high, moderate, low, or very low certainty. | Favourable point estimates were not used to upgrade certainty. |

Supplementary Table S20B. Analytical reference range and imprecision rule

| Item | Final rule |
| --- | --- |
| Analytical reference range | The range from -0.30 to +0.30 Hedges' g was used solely as an analytical reference for imprecision assessment. |
| Clinical interpretation | The range was not treated as a validated minimal clinically important difference, a clinical-equivalence margin, or a threshold for clinical importance. |
| Downgrade scale | Each certainty domain was rated as 0, -1, or -2 levels according to the severity of concern. |
| Double counting | Overlapping concerns were assigned to the most relevant domain and were not counted again in another domain. |

Supplementary Table S20C. Certainty assessment corresponding to Figure 5B for the principal active-versus-control comparisons in the 12-study primary randomized-trial network

| Comparison | Hedges' g [95% CI] | Within-study bias | Reporting/data availability | Indirectness | Imprecision | Heterogeneity | Incoherence | Total downgrade | Final certainty |
| --- | --- | --- | --- | --- | --- | --- | --- | --- | --- |
| Conventional exercise vs control | 0.33 [-0.04, 0.69] | -1 | 0 | -1 | -1 | 0 | 0 | -3 | Very low |
| Pilates vs control | 0.64 [0.18, 1.09] | -1 | 0 | -1 | -1 | 0 | -1 | -4 | Very low |
| Technology-assisted exercise vs control | 0.69 [0.38, 1.01] | -1 | 0 | -1 | 0 | 0 | 0 | -2 | Low |
| Technology-delivered Pilates vs control | 0.69 [0.14, 1.25] | -1 | 0 | -1 | -1 | 0 | 0 | -3 | Very low |

Supplementary Table S20D. Domain-level certainty assessment for additional active-intervention comparisons

| Comparison | Hedges' g [95% CI] | Within-study bias | Reporting/data availability | Indirectness | Imprecision | Heterogeneity | Incoherence | Total downgrade | Final certainty | Interpretation |
| --- | --- | --- | --- | --- | --- | --- | --- | --- | --- | --- |
| Technology-assisted exercise vs conventional exercise | 0.37 [0.11, 0.62] | -1 | 0 | -1 | -1 | 0 | 0 | -3 | Very low | Favourable point estimate, but certainty was very low because of broad technology and comparator nodes and CI overlap with the analytical reference range. |
| Pilates vs conventional exercise | 0.31 [-0.15, 0.77] | -1 | 0 | -1 | -1 | 0 | 0 | -3 | Very low | Insufficient to establish Pilates superiority over conventional exercise. |
| Technology-delivered Pilates vs Pilates | 0.06 [-0.46, 0.58] | -1 | 0 | -1 | -1 | 0 | 0 | -3 | Very low | Does not establish comparable effectiveness or superiority between remote/digital and face-to-face Pilates. |
| Technology-assisted exercise vs Pilates | 0.06 [-0.42, 0.53] | -1 | 0 | -2 | -1 | 0 | 0 | -4 | Very low | Exploratory indirect comparison only; no active-intervention hierarchy can be inferred. |
| Technology-assisted exercise vs technology-delivered Pilates | 0.00 [-0.60, 0.60] | -1 | 0 | -2 | -1 | 0 | 0 | -4 | Very low | Exploratory indirect comparison only with sparse technology-delivered Pilates evidence. |

**Supplementary Table S20E. Domain-level rationale for technology-assisted exercise versus control**

| Domain | Downgrade | Rationale |
| --- | --- | --- |
| Within-study bias | -1 | The contributing randomized-trial evidence was judged as having some concerns overall under RoB2. |
| Reporting/data availability | 0 | No additional downgrade was applied beyond the broader data-availability assessment. |
| Indirectness | -1 | The node combined heterogeneous technology-assisted exercise formats and populations. |
| Imprecision | 0 | The 95% CI was relatively narrower than other principal comparisons and did not cross the null. |
| Heterogeneity | 0 | No additional statistical heterogeneity downgrade was applied beyond indirectness. |
| Incoherence | 0 | No comparison-specific direct-indirect downgrade was applied. |
| Final certainty | Low | Total downgrade of two levels from high-certainty randomized evidence. |

Supplementary Table S20F. Certainty implications of sensitivity analyses

| Sensitivity analysis | **Finding** | **Certainty implication** |
| --- | --- | --- |
| Baseline-imbalance exclusion | Excluding the study with potentially consequential selected-outcome baseline difference produced only small changes in estimates | Supports sensitivity interpretation but does not remove baseline concerns generally |
| Non-exercise/minimal-intervention control sensitivity | Excluding active non-exercise control evidence changed estimates and evidence structure | Control-node heterogeneity contributed to indirectness/applicability concerns |
| Clinical/older-rehabilitation population restriction | Estimates remained broadly favourable but evidence remained clinically heterogeneous | Population heterogeneity continued to limit transitivity |
| Clinical/field-based balance-performance outcome restriction | Estimates attenuated for conventional exercise, Pilates, and technology-delivered Pilates; only technology-assisted exercise retained a CI excluding the null | Outcome-domain heterogeneity contributed to indirectness and imprecision concerns |
| Direct–indirect diagnostics | Potential local disagreement was detected for Pilates versus control in the primary network | Pilates-control certainty downgraded for incoherence |
| Common-effect model | Similar estimates were observed under common-effect modelling | Did not remove clinical heterogeneity concerns |
| AR/VR-only feasibility analysis | Restricted network did not retain the full five-node evidence structure | Protocol-scope and platform-specific conclusions remain limited |

Note. CI, confidence interval; NMA, network meta-analysis; RCT, randomized controlled trial; RoB, risk of bias; SMD, standardized mean difference; VR, virtual reality; AR, augmented reality. Certainty was assessed at the comparison level using a conservative GRADE/CINeMA-informed framework. Judgments incorporated RoB2 findings, random-effects contribution information, clinical indirectness, data-availability concerns, imprecision, heterogeneity, and direct–indirect evidence. Certainty ratings were not based on P-score ordering and were not used to support definitive treatment ranking. The analytical reference range for Hedges’ g was used only for imprecision assessment and was not treated as a disease-specific minimal clinically important difference.

Supplementary Table S21. Reproducibility package inventory, clean-run verification, and software-session information

**Supplementary Table S21A. Final reproducibility package and reporting hierarchy**

| Package component | Submitted filename or folder | Purpose |
| --- | --- | --- |
| Entry script | run_all.R | Single entry point that regenerates final statistical outputs, figure PDFs, verification files, and the file manifest from package-relative paths. |
| Main analysis script | code/Final_NMA_Analysis_Script.R | Final statistical script used to generate estimates, diagnostics, sensitivity analyses, output workbook, and CSV tables. |
| Analysis-ready input workbook | data/Analysis_Ready_Input_Workbook.xlsx | De-identified input data used by the final NMA script. |
| Certainty data | data/Certainty_Assessment_Final.csv | Final domain downgrades and certainty judgements used for Figure 5 and certainty tables. |
| Output workbook | outputs/Final_NMA_Output_Workbook.xlsx | Final output workbook regenerated by run_all.R. |
| Output CSV tables | outputs/csv_tables/ (53 CSV files) | One CSV per final output table included in the regenerated workbook. |
| Figure source data | figure_source_data/ (23 files) | CSV source data for figures, final R scripts for Figures 2-5 and Supplementary Figures S1-S5, and editable Figure 1 Word/PDF/JPG source files with PRISMA count audit retained for audit. |
| Figure scripts | code/figures/ (9 figure scripts plus 1 helper) | R scripts used to regenerate Figures 2-5 and Supplementary Figures S1-S5. |
| Figure PDFs | outputs/figures/ (10 PDFs) | Figure 1 assembled from supplied Word/PDF source; Figures 2-5 and Supplementary Figures S1-S5 regenerated by R from package source data. |
| Verification files | verification/ | Clean-run log, session information, package versions, expected-result checks, verification summary, and SHA256 file manifest. |

**Supplementary Table S21B. Final analysis-layer map**

| Evidence layer | Studies | Analytic arms | Participants | Manuscript use |
| --- | --- | --- | --- | --- |
| Primary randomized-trial network | 12 | 25 | 491 | Main inferential NMA layer. |
| Broader randomized-trial sensitivity network | 14 | 29 | 606 | Directional sensitivity layer adding broader randomized evidence. |
| Figure 5A RoB2 set | 14 source-verified randomized trials | Not applicable | Not applicable | Risk-of-bias summary across primary plus broader-only randomized trials. |
| Figure 5B certainty set | 12-study primary network | 25 | 491 | Four principal active-versus-control certainty ratings. |

**Supplementary Table S21C. Reproducible workflow from extracted arm-level data to network estimates**

| Step | Reproducibility action | Output or verification file |
| --- | --- | --- |
| 1. Run entry script | Run run_all.R from the package root. The script sets package-relative final input and output paths and calls code/Final_NMA_Analysis_Script.R. | run_all.R |
| 2. Read analysis-ready workbook | Read arm-level, study-level, RoB2, full-text decision, intervention-node, and outcome-source sheets. | data/Analysis_Ready_Input_Workbook.xlsx |
| 3. Construct Hedges' g contrasts | Use exact small-sample correction after outcome-direction harmonization. | outputs/csv_tables/15a_source_RCT_primary_Hedges_g.csv |
| 4. Fit NMA models | Fit source-verified primary and broader randomized-trial networks and sensitivity models. | outputs/Final_NMA_Output_Workbook.xlsx |
| 5. Generate diagnostics | Export heterogeneity, inconsistency, direct-indirect, contribution, and sensitivity outputs. | outputs/csv_tables/26_direct_indirect_network.csv; outputs/csv_tables/32_diagnostics.csv |
| 6. Regenerate figure set | Assemble Figure 1 from the supplied Word/PDF source and regenerate Figures 2-5 plus Supplementary Figures S1-S5 from package source data. | outputs/figures/ (10 PDFs) |
| 7. Verify clean run | The clean run regenerated the final output workbook, all 53 final CSV output tables, Figures 2-5 and Supplementary Figures S1-S5 as PDFs from the supplied R scripts, and assembled Figure 1 from the supplied final Figure 1 PDF source, together with final JPG files for document-level figure matching, the clean-run log, software-session information, package-version records, verification summary, and SHA256 file manifest. | verification/verification_summary.txt; verification/FILE_MANIFEST_SHA256.csv |

**Supplementary Table S21D. Output workbook and CSV sheet map**

| Output group | Representative files | Purpose |
| --- | --- | --- |
| Run and count reconciliation | 00_run_status; 00b_count_reconciliation | Documents evidence-layer counts and run status. |
| Study and data audits | 02c_source_verified_study_audit; 08_baseline_audit; 09_concurrent_activity_audit; 13_missing_data_summary | Documents study-design, baseline, activity, and data-availability audits. |
| Effect-size and NMA outputs | 15a_source_RCT_primary_Hedges_g; 17a_source_RCT_primary_vs_ctrl; 21a_formal_RCT_sens_vs_ctrl; 23a_source_RCT_primary_league | Documents Hedges' g inputs, estimates versus control, sensitivity estimates, and league-table estimates. |
| Diagnostics | 26_direct_indirect_network; 27_decomp_design; 28_contribution_matrix_long; 32_diagnostics | Documents direct-indirect, design inconsistency, contribution, and global diagnostic outputs. |
| Certainty and notes | 29_certainty_primary_final; 29b_certainty_all_comparisons_final; 29d_CINeMA_scope_note | Documents final certainty judgements, active-comparison certainty assessments, and scope notes. |
| Figure source data | Figure1_PRISMA_counts; Figure5A_RoB2_all14; Figure5B_primary_certainty; SupplementaryFigureS5_RoB2_all14 | Documents figure-specific data sources, including Figure 1 provenance and Figure 5 RoB2/certainty sources. |

**Supplementary Table S21E. Statistical methods and R functions requiring reproducibility verification**

| Analysis step | Final implementation |
| --- | --- |
| Continuous-outcome effect size | Hedges' g with exact small-sample correction after direction harmonization. |
| Network estimation | Random-effects network meta-analysis for primary and broader randomized-trial evidence layers. |
| Sensitivity analyses | Baseline-imbalance exclusion, minimal-control restriction, clinical/older-rehabilitation restriction, field-balance outcomes, and common-effect model. |
| Diagnostics | Global diagnostics, direct-indirect decomposition, design inconsistency, and random-effects contribution matrix. |
| Ranking summaries | P-scores retained only as exploratory supplementary summaries in fixed conceptual order. |
| Certainty | Conservative GRADE/CINeMA-informed comparison-level assessment using 0/-1/-2 domain downgrades. |

**Supplementary Table S21F. Software-session information**

| Item | Recorded information |
| --- | --- |
| Session file | verification/session_info.txt |
| Clean-run outputs | outputs/; verification/ |
| Clean-run CSV count | 53 |
| Figure script count | 9 figure scripts plus 1 shared helper |
| Figure PDF count | 10 |
| Package manifest | verification/FILE_MANIFEST_SHA256.csv |

Note. NMA, network meta-analysis; RCT, randomized controlled trial; REML, restricted maximum likelihood; SD, standard deviation; SMD, standardized mean difference. The reproducibility package supports the source-verified RCT-compatible primary and broader randomized-trial analyses, associated diagnostics, sensitivity analyses, tables, and figure-source files. The primary manuscript conclusions are based on the source-verified randomized-trial evidence layers.

Supplementary Table S22. Protocol concordance and analytical deviations

**Supplementary Table S22A. Protocol registration and final review framework**

| **Item** | **Registered or original framework** | **Final revised framework** | **Concordance judgment** |
| --- | --- | --- | --- |
| Registration platform | PROSPERO | PROSPERO | Concordant |
| Registration number | CRD420261383909 | CRD420261383909 | Concordant |
| Original registered title | Pilates and AR/VR-Based Technology-Assisted Exercise for Improving Balance and Postural Control: A Systematic Review and Exploratory Network Meta-Analysis | Retained as the registered-title basis, with final reporting clarifying a broader technology-assisted exercise node | Partly concordant; intervention-scope broadening explicitly acknowledged |
| Review design | Systematic review and exploratory network meta-analysis | Systematic review and exploratory network meta-analysis | Concordant |
| Broad clinical target | Balance and postural-control outcomes | Balance-, postural-control-, and fall-risk-related outcomes, with fall-related proxy evidence retained only in the broader/sensitivity layer | Largely concordant; fall-related proxy evidence separated from primary inference |
| Main comparative framework | Pilates, AR/VR-based technology-assisted exercise, conventional exercise, and control | Pilates, technology-assisted exercise, technology-delivered Pilates, conventional exercise, and control | Partly concordant; technology-assisted scope broadened and technology-delivered Pilates coded separately |
| Final inferential layer | Controlled intervention evidence eligible for NMA | Source-verified randomized-trial primary NMA | Conservative final restriction |
| Main evidence status | Exploratory | Exploratory and hypothesis-generating | Concordant |

Supplementary Table S22B. Concordant protocol elements retained in the final review

| **Protocol or planned-review element** | **Final implementation** | **Status** |
| --- | --- | --- |
| Systematic literature search | Eight bibliographic/register sources searched through 2 May 2026 | Retained |
| Adult/older-adult/rehabilitation balance-related scope | Adults, older adults, athletic, neurological, and rehabilitation-related populations with eligible balance/postural-control/fall-related outcomes were considered | Retained, with transitivity limitations explicitly assessed |
| Comparative intervention design | Studies had to include at least two eligible intervention/comparator nodes | Retained |
| Balance and postural-control focus | Primary network restricted to balance or postural-control endpoints | Retained |
| Network meta-analysis framework | Frequentist network meta-analysis used to compare multiple eligible nodes | Retained |
| Standardized effect metric | Standardized mean difference used because outcomes were measured on different scales | Retained, specified as Hedges’ g with exact small-sample correction |
| Outcome-direction harmonization | Outcomes harmonized so that positive effects consistently indicated better performance | Retained and documented |
| Risk-of-bias assessment | Randomized trials assessed using RoB2 | Retained, restricted to source-verified randomized trials |
| Certainty assessment | Certainty assessed using a GRADE/CINeMA-informed approach | Retained and made more conservative |
| Sensitivity/robustness checks | Sensitivity analyses used to assess analytical fragility | Retained as final analytical-fragility checks |
| Exploratory ranking output | Ranking-related output treated as exploratory only | Retained, with P-scores displayed only in fixed conceptual order |

Supplementary Table S22C. Protocol deviations and implementation clarifications

| **Deviation or clarification** | **Deviation level** | **What changed** | **Reason for change** | How it was handled in the final review |
| --- | --- | --- | --- | --- |
| Technology-assisted exercise node | Major operational protocol deviation | AR/VR-centred registered wording was operationalized as a broader digitally supported technology-assisted exercise node. | The eligible evidence base included exergaming, motion-sensing and interactive digital exercise beyond AR/VR. | Handled transparently in Methods, limitations, and sensitivity interpretation. |
| Technology-delivered Pilates | Methodological clarification | Remote or digital Pilates delivery was coded separately from non-digital Pilates. | Delivery mode may affect supervision, adherence and implementation context. | Reported as a distinct sparse node with very low certainty. |
| Primary randomized-trial inference | Conservative restriction | Primary inference was restricted to source-verified randomized trials. | Source verification identified one nonrandomized controlled study. | Nonrandomized evidence was excluded from randomized-trial NMA. |
| Broader proxy and pilot evidence | Analytical clarification | Fall-related proxy and pilot/postural-sway evidence was retained only in the broader randomized-trial sensitivity layer. | These outcomes were not fully exchangeable with primary balance/postural-control outcomes. | Broader layer interpreted directionally and not confirmatorily. |
| Lee and Yoo same-node arms | Analytical implementation | Yoga and Self-Otago source arms were combined as one conventional-exercise analytic arm. | Both source arms belonged to the same randomization, outcome, time point and intervention node. | Combined before contrast construction to avoid double-counting. |
| Effect-size metric | Analytical clarification | Post-intervention Hedges' g was used. | Change-score SDs and adjusted estimates were not consistently available. | Baseline comparability audit and sensitivity analysis were added. |
| Screening workflow | Workflow clarification | One reviewer performed initial screening and uncertain records were verified. | This reflected the implemented review workflow. | Reported as a protocol-transparency item without changing eligibility criteria. |
| Data extraction workflow | Workflow clarification | Initial extraction was verified using source and arm-level checks. | This reflected the implemented extraction workflow. | Reported transparently with final source-verification outputs. |
| Author contact | Protocol deviation/reporting limitation | No author contact occurred for missing numerical data. | The final analyses used only extractable or derivable public report data. | No unpublished arm-level numerical data were obtained or incorporated. |
| ROBINS-I | Tool-application clarification | ROBINS-I was not applied to randomized-trial NMA evidence. | The nonrandomized controlled study was excluded from randomized-trial NMA rather than pooled. | RoB 2 and CINeMA/GRADE judgments were restricted to randomized-trial evidence. |
| Supplementary search activities | Protocol-transparency clarification | Google Scholar, citation chasing, trial-registration and unpublished-evidence intentions are reported as implemented or not implemented from the final audit record. | The final evidence base used public reports with compatible numerical data. | Unimplemented or noncontributory steps are acknowledged in limitations and S22. |
| Ranking outputs | Reporting clarification | P-scores were retained only as exploratory fixed-order summaries. | Sparse and heterogeneous networks do not support a definitive treatment hierarchy. | No superiority hierarchy or clinical recommendation is inferred from P-scores. |

Supplementary Table S22D. Rationale for the intervention-scope clarification

| Issue | Final explanation |
| --- | --- |
| Protocol-relevant clarification | The registered title emphasized AR/VR-based technology-assisted exercise, whereas the final technology-assisted exercise node also included exergaming, motion-sensing, interactive digital exercise, and related technology-supported exercise formats. |
| Reason for broader node | The retrieved comparative evidence did not support a fully connected AR/VR-only network. A broader digitally supported exercise node preserved analyzable randomized evidence while increasing indirectness. |
| What this decision did not do | It did not introduce a new population, outcome domain, comparator class, or post hoc node based on favourable results. |
| What this decision changed | It broadened the intervention definition and therefore increased clinical and technological indirectness. |
| How the risk was assessed | Technology-modality audit, AR/VR-only feasibility analysis, transitivity assessment, contribution-matrix interpretation, and certainty downgrading were used to contextualize this scope clarification. |
| Implication for interpretation | Technology-assisted exercise should be interpreted as a broad digital/technology-supported exercise category, not as platform-specific AR/VR efficacy. |
| Reporting boundary | Conclusions should avoid definitive treatment ranking, platform-specific claims, or AI/algorithmic efficacy claims. |

**Supplementary Table S22E. Protocol concordance and deviation impact on each main result**

| **Result or inference** | **Relevant concordance/deviation issue** | **Impact on interpretation** |
| --- | --- | --- |
| Conventional exercise vs control | RCT restriction; control-node heterogeneity; sparse direct evidence | Favourable point estimate but very low certainty |
| Pilates vs control | Sparse direct evidence; active non-exercise control issue; potential direct–indirect disagreement | Favourable estimate but very low certainty and incoherence concern |
| Technology-assisted exercise vs control | Major intervention-scope broadening; broad technology node; varied control subtypes | Favourable estimate but low certainty and no platform-specific inference |
| Technology-delivered Pilates vs control | Separate node added for digital/remote Pilates delivery; sparse evidence | Favourable estimate but very low certainty |
| Active-intervention comparisons | Several comparisons indirect-only or single-study direct evidence | No definitive hierarchy among active interventions |
| P-score summaries | Ranking output retained only as exploratory | No treatment-ranking conclusion |
| Broader randomized-trial network | Added fall-related proxy and pilot/broader evidence | Sensitivity layer only; did not replace primary inference |
| Certainty assessment | Conservative final reassessment | Low or very low certainty; no moderate-certainty claim retained |

Note. AR, augmented reality; CI, confidence interval; NMA, network meta-analysis; PROSPERO, International Prospective Register of Systematic Reviews; RCT, randomized controlled trial; RoB2, revised Cochrane risk-of-bias tool for randomized trials; SMD, standardized mean difference; VR, virtual reality. This table summarizes protocol concordance, scope clarifications, and analytical deviations relevant to interpreting the final review.
